# Detection and location of EEG events using deep learning visual inspection

**DOI:** 10.1371/journal.pone.0312763

**Published:** 2024-12-23

**Authors:** Mohammad Amin Fraiwan

**Affiliations:** Department of Computer Engineering, Jordan University of Science and Technology, Irbid, Jordan; Bayer Crop Science United States: Bayer CropScience LP, UNITED STATES OF AMERICA

## Abstract

The electroencephalogram (EEG) is a major diagnostic tool that provides detailed insight into the electrical activity of the brain. This signal contains a number of distinctive waveform patterns that reflect the subject’s health state in relation to sleep, neurological disorders, memory functions, and more. In this regard, sleep spindles and K-complexes are two major waveform patterns of interest to specialists, who visually inspect the recordings to identify these events. The literature typically follows a traditional approach that examines the time-varying signal to identify features representing the events of interest. Even though most of these methods target individual event types, their reported performance results leave significant room for improvement. The research presented here adopts a novel approach to visually inspect the waveform, similar to how specialists work, to develop a single model that can detect and determine the location of both sleep spindles and K-complexes. The model then produces bounding boxes that accurately delineate the location of these events within the image. Several object detection algorithms (i.e., Faster R-CNN, YOLOv4, and YOLOX) and multiple backbone CNN architectures were evaluated under a wide range of conditions, revealing their true representative performance. The results show exceptional precision (>95% mAP@50) in detecting sleep spindles and K-complexes, albeit with less consistency across backbones and thresholds for the latter.

## Introduction

**Fig 1:**
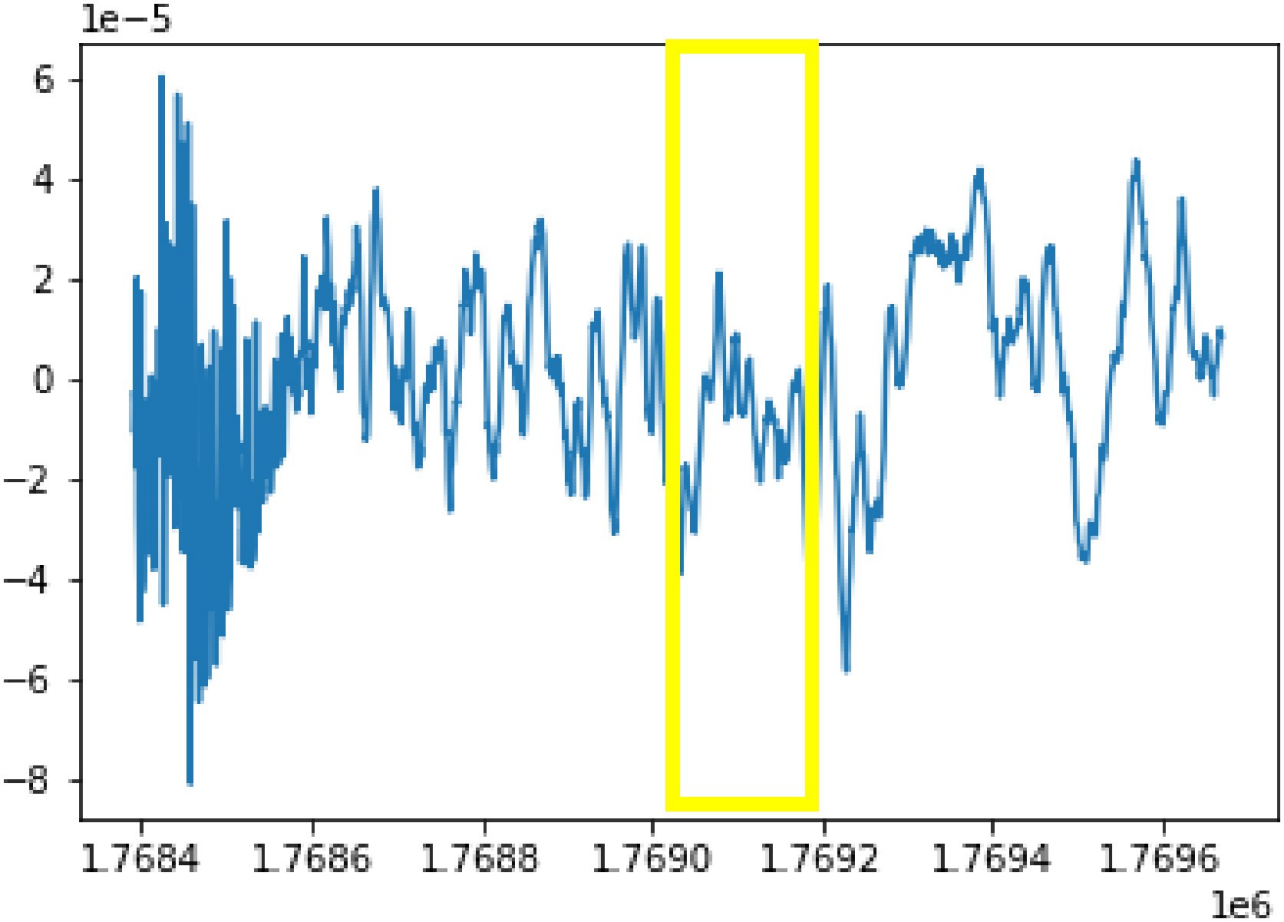
The time-varying waveform of the sleep spindles in an EEG recording.

**Fig 2:**
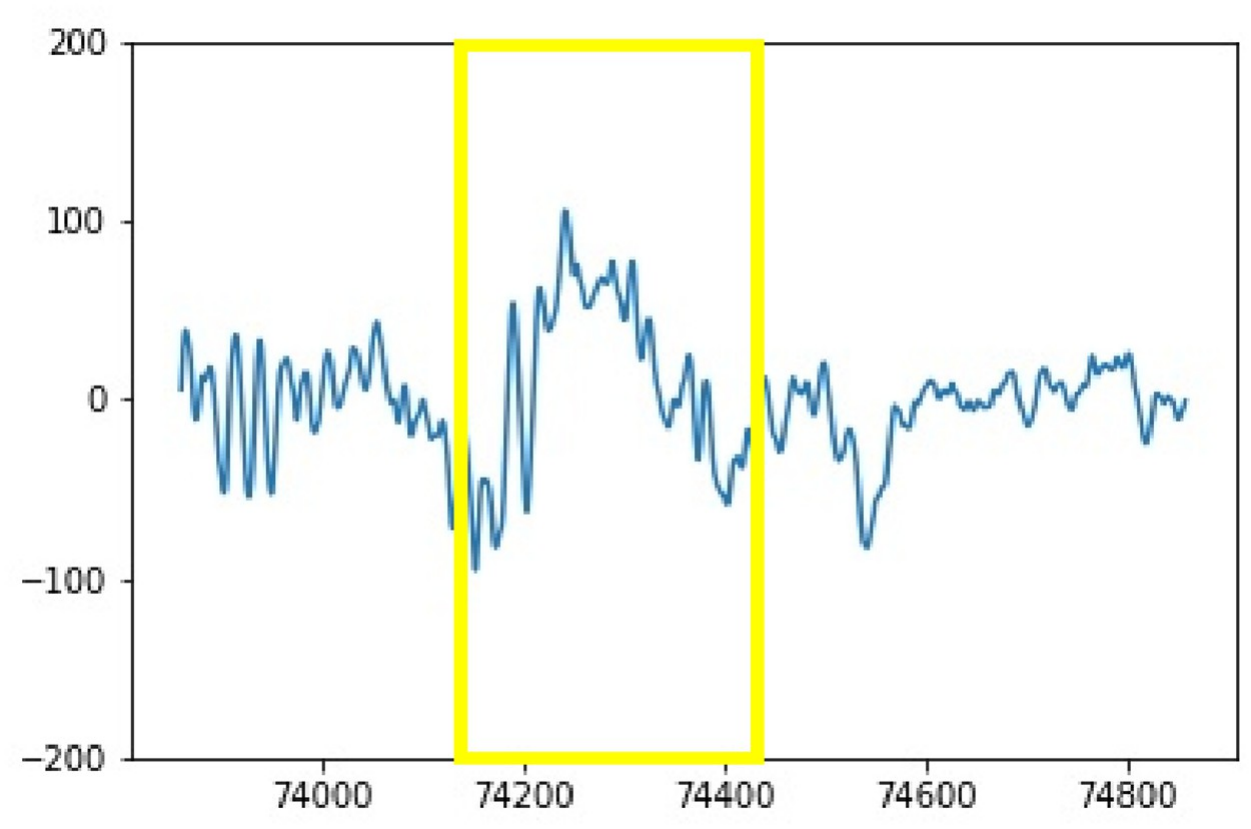
The time-varying waveform of the K-complexes in an EEG recording.

The Electroencephalogram (EEG) is a diagnostic tool that involves placing electrodes on the scalp to measure brain cell activity and is widely used in neurology and sleep studies. Special events and patterns in EEG recordings provide significant clues about the function and health of the brain. Among these waveforms, sleep spindles and K-complexes, typically occurring during non-rapid eye movement (NREM) sleep stage N2, are significant. Sleep spindles are characterized by a frequency of 11–16 Hz, an amplitude below 50*μ*V, and a duration of at least 0.5 seconds [1], see Figs 1 and 2. They signify neural synchronization, particularly within thalamocortical circuits, promoting sleep stability and resilience against disturbances. Additionally, they are implicated in brain plasticity, reflecting synaptic changes and cortical reorganization. On the other hand, K-complexes exhibit a biphasic morphology, featuring 200-millisecond waves characterized by a positive ascent followed by a negative descent spanning 550 milliseconds, culminating in a prolonged positive peak lasting 900 milliseconds [2]. Changes in the spindle and K-complex patterns have been linked to various brain pathologies such as autism, insomnia, epilepsy, obstructive sleep apnea, and schizophrenia, highlighting their diagnostic importance [3]. Determining the location and count of sleep spindles and K-complexes have typically been performed by trained specialists, which is a time-consuming and tedious process fraught with mistakes.

The majority of the literature have addressed the EEG inspection problem by devising separate methods to detect each type of waveform patterns. This is because these methods worked for one type but not the other, or the extracted features are indicative of one pattern but not the other. Furthermore, combining the features of more than one pattern of interest may not generate the desired performance especially in traditional artificial intelligence (AI) methods were overfitting and lack of generalization maybe a problem. However, if a combined model can achieve performance comparable to individual models, it offers significant advantages. Specifically, a single model would require less memory during runtime and reduce computational demands during training.

Typically, signal processing techniques were used to preprocess the EEG signal (e.g., segmentation) and extract distinct features of K-complexes or sleep spindles for the classification algorithms. For instance, Schönwald et al. [4] used Gabor transform to extract useful parameters for spindles detection. Lacourse et al. [5] proposed four statistical features of the EEG signals to design a spindle detector with a reported precision of 74%. In another study, Zhuang et al. [6] used probability estimation, which works by performing the CWT with Mexican hat wavelet function on a sliding window of the EEG signal, to detect spindles with corresponding probabilities (i.e., numbers representing a sort of confidence score in the specific detection). Ventouras et al. [7] used band pass filtration of EEG signals to feed an artificial neural network (ANN) but without feature extraction. They reported a sensitivity range of 79.2% − 87.5%, but did not report the precision values for the detector. Similarly, Yücelbas et al. [8] fed the ANN with features extracted using four frequency domain methods (i.e., fast Fourier transform, Welch, auto regressive, and multiple signal classification). They reported an accuracy range of 80.8% to 84.8%. Furthermore, the results were improved to an accuracy range of 82% to 94.8% by applying principal component analysis (PCA) to the extracted features. In another study, Li et al. [9] used the decision tree classification algorithm to develop a model that detects sleep spindles with features extracted using the complex demodulation method (CDM). They reported an accuracy range of 63%-81%. In a similar methodology, studies for K-complex classification used multi-domain feature extraction and fractal dimension analysis of time-frequency images, coupled with different classification algorithms like K-means, least square support vector machine, and Naïve Bayes [10–12]. Other studies used chaotic features combined with modified extreme learning machine-generalized radial basis function were used to design a K-compelex classification algorithm [13]. Furthermore, the application of time-frequency analysis methods on EEG recordings was explored for K-complex detection, including singular value decomposition, discrete wavelet transform (DWT), and variational mode decomposition [2, 14].

The problem of combined detection of EEG events was largely overlooked in the literature. Lajnaf et al. [15, 16] used a modified version of the wavelet transform (i.e., tunable Q-factor) in conjunction with morphological analysis to develop a tool called Spinky (spindle and K-complex detector). Such signal processing and explicit feature extraction have been extensively reported in the literature with small to limited success as demonstrated by the reported low F1 scores (i.e., 63% − 70%). However, the past decade has witnessed an exponential growth in the number and type of applications benefiting from AI and deep learning [17, 18]. In the context of this problem, several systems have recently been introduced. Tapia-Rivas et al. [19] proposed the sleep EEG event detector (SEED), which combines feature extraction using convolutional neural networks (CNNs) and time series processing of recurrent neural networks (RNN). The model processes each sample to conclude if it is part of the event under consideration. They detected spindles with and F1 score of 80.5%, but the K-complexes detection was slightly higher at 83.7%. Chambon et al. [20] went further and considered adding a third type of EEG events (i.e., arousal) to the spindles and K-complex combined detector. They proposed “Dreem One Shot Event Detector (DOSED)”, which is based on a custom-built deep learning architecture involving spatial filtration, convolution, and temporal features, albeit the reported precision was low. Combining unsupervised pretraining and supervised finetuning, such as in object detection with transformers (e.g., DEtection TRansformer (DETR) [21] and Vision Transformer (ViT) [22]), has been shown to perform comparably well to models like Faster R-CNN or YOLO. However, these methods often require large-scale training data, similar to other unsupervised pre-training approaches. In EEG applications, the amount of annotated data is limited. Despite this, the method of unsupervised pre-training described in Dai et al. [21] (i.e., using cropped patches of images for pre-training) and others present an intriguing avenue for exploration.

This work aims at designing a system capable of detecting both sleep spindles and K-complexes in EEG signals. The methodology employed here is novel as it involves visual inspection of the EEG waveform, akin to how specialists visually analyze the waveform, but with the efficiency and precision of computers. This unique approach yields superior performance, facilitating seamless integration into smart devices for automatic detection of pertinent EEG events. To accomplish this, we leverage recent advancements in object detection techniques utilizing deep learning. EEG waveform images are fed into a deep learning model, which automatically extracts key features indicative of the targeted events. The model then produces bounding boxes that accurately delineate the location of the spindles within the image. Several object detection algorithms (i.e., Faster R-CNN, YOLOv4, and YOLOX) and multiple backbone CNN architectures were evaluated under a wide range of conditions that reveal their true representative performance. The paper follows the outlined structure: The materials and methods section commences with a discussion on the dataset and the creation of EEG waveform images, followed by an exploration of the object detection models and their architecture. Performance metrics and the experimental setup are also covered in this section. The results section presents and discusses the performance evaluation results. The concluding remarks are provided in the conclusion section.

## Materials and methods

### EEG data

The data used in this work was obtained from two sources: the “Dreams K-complexes database” [23] and the “Montreal Archive of Sleep Studies (MASS)” [24]. The former consists of ten 30-minute EEG recordings of the central EEG channel extracted from polysomnographic (PSG) sleep recordings, sampled at a rate of 200 Hz. K-complexes in recordings 1 to 6 were annotated by two experts, while recordings 7 to 10 were annotated by one expert only. On the other hand, the MASS dataset comprises 19 full-night EEG recordings from PSG sleep recordings involving nineteen subjects (eight males, eleven females), with a sample rate of 256 Hz. Sleep spindles were identified by an expert in the field. For further technical details, see O’Reilly et al. [25].

**Fig 3:**
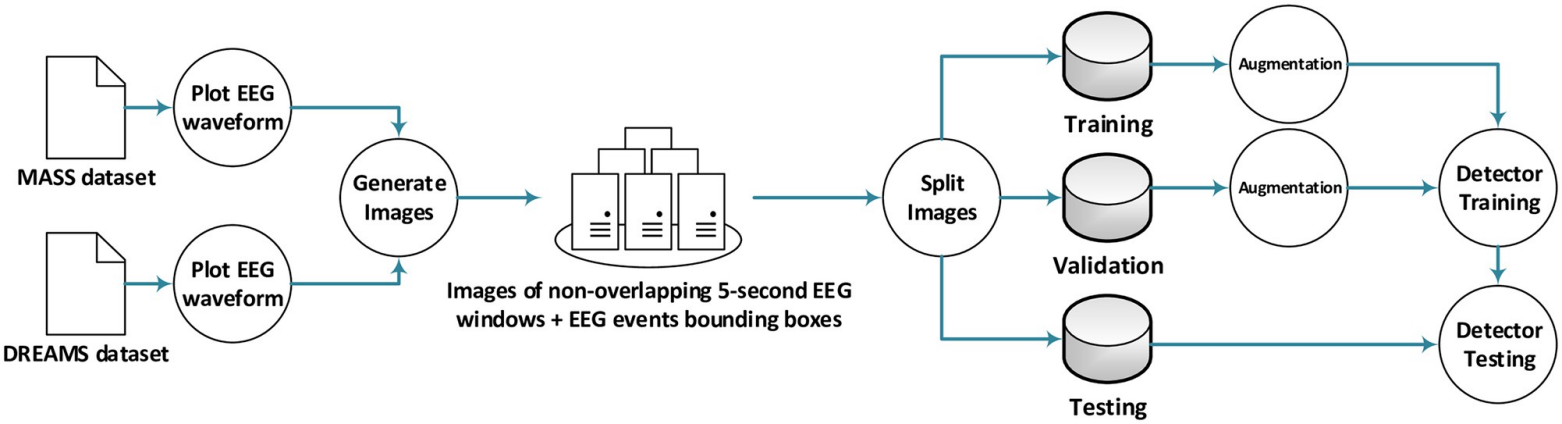
The general dataflow process used to build and test the detectors. The data split is performed according to the specific strategy used (e.g., random, cross-validation, leave-subject-out, etc.).

The data flow process is shown in Fig 3. The data points provided by the two datasets were plotted according to their respective sampling frequencies (i.e., a 200 Hz sampling rate used by DREAMS dataset means a data point is plotted every 5 ms, while a 256 Hz sampling rate used by MASS dataset means a data point is plotted every 3.90625 ms). No resampling was done. EEG signal waveforms were segmented into images containing five second sliding non-overlapping views. This resulted in 271 K-complex images, and 1044 sleep spindle images. The location of the EEG events (i.e., spindles or K-complexes) is provided by the datasets. For example, the DREAMS database provides the location of the spindles in terms of two numerical values representing the starting time and duration of the event. This information is converted into a bounding box containing the spindle and appropriate for object detection algorithms.

Only image augmentation was performed; the signals were not manipulated in any way. The image augmentation included: (1) Random x-scaling and y-scaling in the range [0.9,1.1]; (2) Horizontal and vertical translation using a range of [-30,30] pixels. The bounding boxes were adjusted accordingly; if the augmentation resulted in an invalid image (i.e., out of bounds event), it was redone. (3) Random alteration of colors by adjusting hue, saturation, and brightness within the range of [-0.2,0.2].

### Visual inspection models

**Fig 4:**
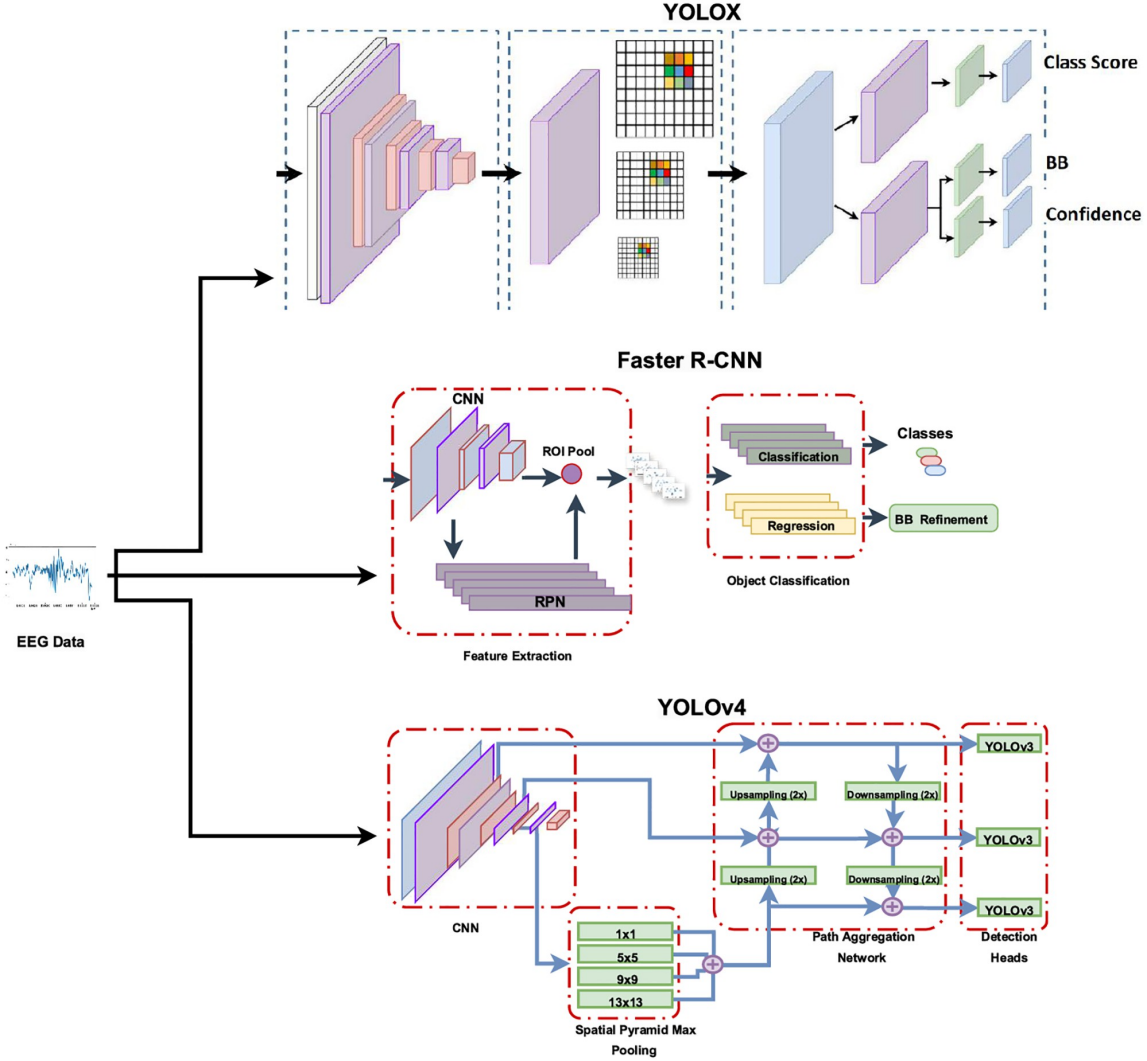
A block diagram of the structure of the three object detection methods.

Three object detection models were utilized independently for detecting EEG spindles and K-complexes, see Fig 4: Faster R-CNN, YOLOv4, and YOLOX. In the following few paragraphs, we go through some details of these three architectures and important implementation parameters.

- Faster R-CNN. This method generates and refines multiple bounding box suggestions to achieve its objective. The algorithm involves three key steps: (1) Proposing regions in the image likely to contain objects of interest, represented as bounding boxes. (2) Extracting indicative features of objects by accessing specific layers of a CNN backbone model. For this step, eight CNN models were tested: The ‘relu5’ layer form AlexNet [26], the ‘res4b_relu’ layer from ResNet-18 [27], the ‘activation_40_relu’ layer from ResNet-50, the ‘res4b22_relu’ from ResNet-101, the ‘relu5_4’ layer from VGG19 [28], the ‘mixed7’ layer from Inceptionv3 [29], the ‘inception_4d-output’ layer from GoogleNet [30], and the ‘fire5-concat’ layer from SqueezeNet [31]. (3) Classifying the detected objects by assigning a class, based on the extracted features, to each object within a suggested bounding box.
  The Faster R-CNN model utilizes a region proposal network (RPN) for object detection, distinguishing it from its predecessors R-CNN and Fast R-CNN. When implementing Faster R-CNN, various components such as an RPN, region of interest (ROI) max pooling layer, and specialized regression and classification layers are integrated into the network. The RoI pooling layer ensures consistent input sizes for fully connected layers, accommodating variations in region sizes. For Squeezenet, GoogleNet, and ResNet, an ROI pooling layer of size [14 14] is inserted after the feature extraction layer and of size [17 17] for Inceptionv3, while for Alexnet and VGG19, the last max pooling layer is replaced with new ROI pooling layers of sizes [6 6] and [7 7], respectively.
- YOLOv4: The YOLOv4 detector works by dividing the image into a grid, based on which a set of predefined bounding boxes are refined. If the center of a bounding box falls within a certain grid, the grid’s parameters are used to update the bounding box. The initial bounding boxes are determined based on the training data. The structure of the YOLOv4 detector is comprised of three main components: (1) Feature extraction backbone: In a similar fashion to Faster R-CNN, a CNN is used to extract indicative features of the objects of interest. For this part, six CNN models were tested: VGG-19 with detection from layer ‘relu5_4’, Tiny Coco (i.e., tiny YOLOv4 coco) [32] with detection from layers ‘conv_31 and ‘conv_38, ResNet-18 with detection head at layer ‘res5a_relu’, Small coco (i.e., csp darknet53 coco) with detection from layers ‘conv_140’, ‘conv_151’, and ‘conv_162’, ResNet-50 with detection from layers ‘activation_22_relu’ and ‘activation_40_relu’, and ResNet-101 with detection from layer ‘res4b22_relu’. (2) Neck: This component serves as a bridge between the feature extraction network and the detection heads, consisting of two main parts: spatial pyramid pooling (SPP) and path aggregation network (PAN). SPP utilizes max pooling kernels of varying sizes (1 × 1, 5 × 5, 9 × 9, and 13 × 13) with a stride value of 1 to merge low-resolution feature maps, capturing essential features. The inclusion of multiple kernels enhances feature extraction, expanding the receptive field of backbone features and improving the network’s accuracy in detecting smaller objects. The concatenated feature maps from SPP integrate with high-resolution feature maps through PAN. PAN employs both upsampling and downsampling operations to establish pathways from bottom-up and top-down directions, enabling the combination of low-level and high-level features. Utilizing a set of aggregated feature maps, PAN generates predictions. Within the YOLOv4 network, three detection heads exist, each being a YOLOv3 network responsible for computing final predictions. The YOLOv4 network produces feature maps of dimensions 19 × 19, 38 × 38, and 76 × 76 to predict object positions. On the other hand, the smaller tiny YOLOv4 network utilizes only two YOLOv3 detection heads with feature maps sized 13 × 13 and 26 × 26 for prediction computations. (3) Head: This component utilizes the aggregated feature maps generated by the PAN to derive three attributes for each anchor box, representing a proposed bounding box around a specific object, these are: (a) The objectness score, measured by the intersection over union (IoU), which computes the intersection area divided by the union area of the proposed bounding box and the ground truth. (b) The adjusted location of the bounding box in terms of coordinate offsets. (c) The class label of the object enclosed within the bounding box, expressed as the probability of belonging to a certain class.
- YOLOX: This model is one of the many later releases of the YOLO family of object detection models, which appeared in the work of the Ge etal. [33]. However, in contrast to YOLOv4, it is an anchor-free single stage model. Rather than using the memory-intensive predefined anchor boxes, YOLOX directly detects objects by locating their centers. It predicts bounding boxes in terms of dimensions by dividing the input image into a grid of three different scales and utilizing grid points as the top-left offsets of the bounding boxes. As grids can be recalculated according to image size, YOLOX facilitates tile-based training, allowing the network to be trained on patches and subsequently utilized for inference on full-size images. The YOLOX architecture is composed of three structural components: backbone for feature extraction, neck, and head. Two backbone CNN models were tested: Tiny Coco and Small Coco. The neck consists of a feature pyramid network (FPN), which calculates feature maps and corresponding grids across different scales, alongside a PAN that merges low-level and high-level features. This component combines feature maps from backbone layers and feeds them into the head at three distinct scales (i.e., 2^8^,2^9^, and 2^10^). The decoupled detection head then converts the combined features into three separate feature channels, including: (1) An IoU objectness score indicating the confidence level that the corresponding bounding box contains an object of interest. (2) Classification scores for each class, representing the likelihood of the object belonging to a specific class. (3) Coordinates and dimensions of each bounding box.

### Models evaluation metrics

The visual inspection models were evaluated, and their performance was assessed for effectiveness in detecting sleep spindles and K-complexes within EEG waveforms. To this end, the average precision (AP) and mean average precision (mAP) were used. Average precision measures the ability of the model in identifying relevant objects within a single image taking into account both precision and recall, see Eqs 1 and 2. It calculates the area under the precision-recall curve, see Eq 3, providing a comprehensive assessment of the model’s performance across different bounding box overlap thresholds. Mean average precision, on the other hand, extends this evaluation to multiple images and objects by computing the average of the individual average precisions, see Eq 4. This metric offers a holistic view of the model’s performance across an entire dataset, accounting for variations in detection accuracy. Such evaluation follows the primary challenge metric used in the COCO 2017 competition.

$$\begin{array}{c} \text{recall}=100\%\times \frac{\text{true}\;\text{positives}}{\text{positives}} \end{array}$$ (1)

$$\begin{array}{c} \text{precision}=100\%\times \frac{\text{true}\;\text{positives}}{\text{predicted}\;\text{positive}} \end{array}$$ (2)

$$\begin{array}{c} \text{AP}=100\%\times \int_{r=0}^{1}p\left(r\right) \end{array}$$ (3)

$$\begin{array}{c} \text{mAP}=100\%\times \frac{1}{6}\sum_{j\in \{0.5,0.6,0.7\}}\sum_{i\in \{\text{SS},\text{KC}\}}{\text{AP}}_{i}^{j} \end{array}$$ (4)

### Models evaluation setup

All models underwent assessment using identical hyperparameters and conditions outlined as follows: The stochastic gradient descent with momentum (SGDM) algorithm was utilized for network updates during training [34]. No layers from the model were frozen. The models were re-trained starting with the weights of the pre-trained models. The maximum number of epochs was capped at 30, a choice made to balance training duration and performance stability. The initial learning rate, minimum batch size, L2 regularization factor, and momentum were set respectively to 0.001, 2, 0.0001, and 0.9. No fine-tuning was performed. It is widely recognized that deep learning models can benefit from a larger number of training samples. Therefore, three different options for dataset training/validation/testing splits were implemented (i.e., 50/10/40, 60/10/30, and 80/10/10). Another aspect of evaluation concerns the quality of generated bounding boxes, measured in terms of intersection over union (IoU) with respect to the ground truth. The IoU is calculated as the intersection area divided by the union area of the proposed bounding box and the ground truth. Only bounding boxes exceeding a certain IoU threshold were considered correct. Three thresholds were evaluated (i.e., 50%, 60%, and 70%). Notably, the standard acceptable IoU overlap threshold in literature stands at 50% [35]. The evaluation was performed using the unbalanced dataset. However, further evaluation was performed by balancing the dataset using two methods: (1) Increasing the number of K-complex images via augmentation, which we show to be a misleading way to improve performance. (2) Reducing the number of sleep spindle images to match that of the K-complex.

## Results

In the following paragraphs, we present the results from evaluating the proposed EEG events visual inspection. A total of 144 experimental runs were conducted over two months, encompassing various combinations of object detectors, CNNs, IoU thresholds, and data splits. These results provide a clear picture of performance trends and relative strengths. Additionally, 5-fold cross-validation and leave-subject-out strategies were used for further evaluations in selected scenarios. The experiments aimed at answering the following questions:

1. Is it possible to develop a high-performing combined model for EEG event detection and location, instead of individual models for each event type?
2. Does this combined model produce better performance than individual models for each target? Which object detector-CNN combination produces the best performing EEG visual inspection model?
3. How does increasing the IoU overlap threshold affect the precision of the models?
4. How does increasing the percentage of the dataset used for training the models affect performance?
5. Does balancing the dataset improve detection precision? How do various balancing methods affect precision?

**Fig 5:**
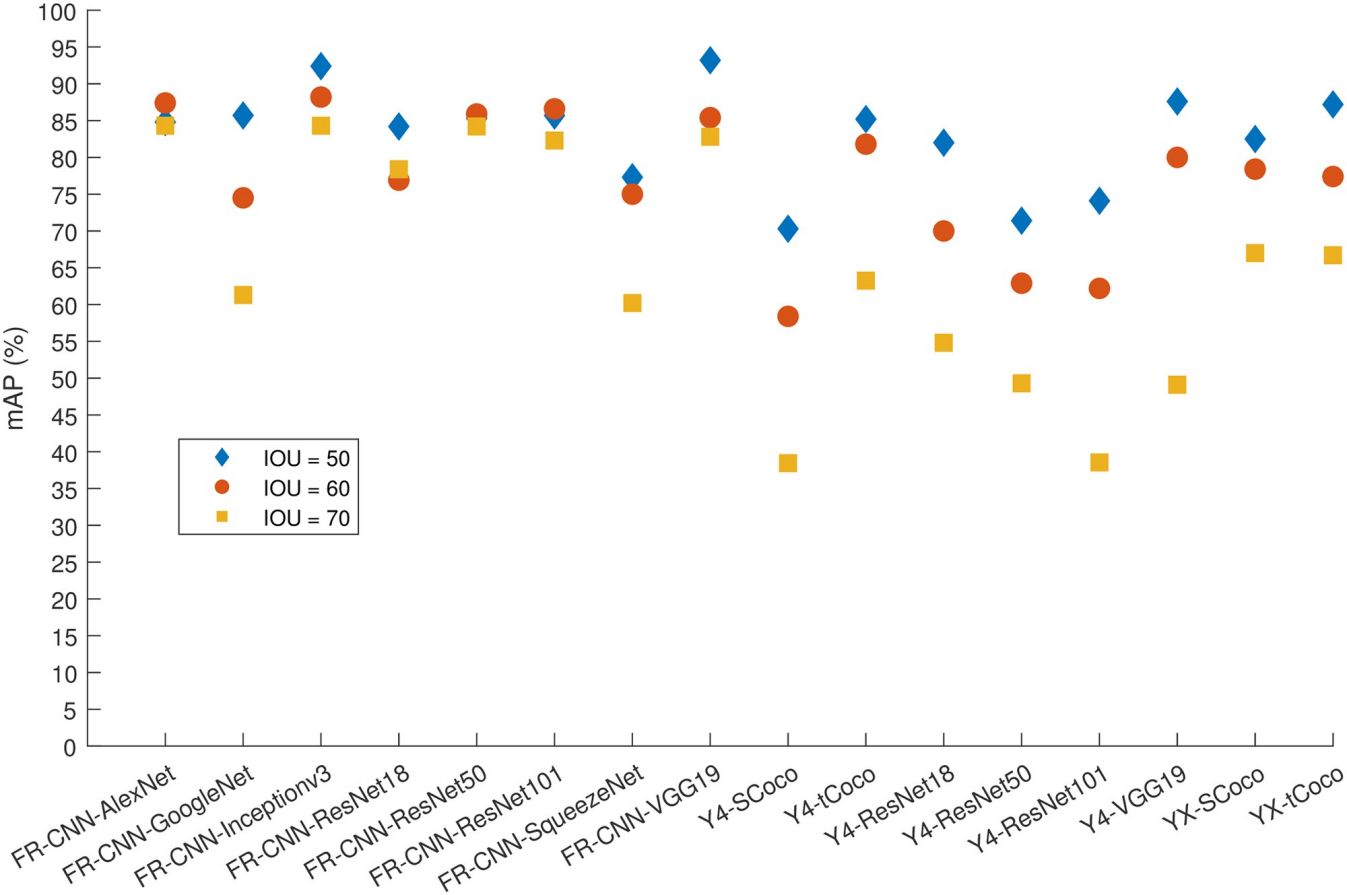
The mAP for all algorithms and 50% of the data used for training.

Fig 5 presents the mAP for the three detectors and two classes of EEG events (i.e., sleep spindles and K-complexes) using 50% of the data for training with the full results available in the supporting tables and figures. The 50% proportion of data is small and serves to establish baseline results. Furthermore, the figure and supporting tables illustrate the effect of increasing the IoU threshold. It is immediately evident that exceptional precision (i.e., > 95%) is achievable for identifying and locating sleep spindles when using most detector-CNN combinations. Equally apparent is the disparity in detection performance between sleep spindles and K-complexes, with the latter achieving a maximum AP of 88.2% using Faster R-CNN and VGG19. The precision of detecting sleep spindles using Faster R-CNN and YOLOX was not significantly affected by increasing the IoU threshold to 70%, and most combinations using these two methods maintained a high value of $AP_{SS}^{70}>90\%$. The sole exception was the Faster R-CNN SqueezeNet model, which does not appear to be well-suited for this problem and experimental setup in comparison to the other CNN backbones for Faster R-CNN. It consistently produced the lowest AP values for the two classes and did not benefit greatly from additional training data. Similarly, the AP of the GoogleNet model dropped drastically for K-complex detection with a 70% threshold. In contrast, the ResNet models exhibited the most consistency, albeit at around 70% precision. Nonetheless, the 50% threshold remains a viable and good option for this type of detection problems.

It should be noted that some entries in the tables may seem in error or inconsistent (e.g., for Faster R-CNN using ResNet18, the rise of *AP*_*SS*_ @ 60% in comparison to *AP*_*SS*_ 50% in S1 Table). However, the analysis or precision needs to carefully consider Eq 2, which indicates that maybe with higher IoU threshold, less bounding boxes were predicted positive and hence a smaller denominator will yield a higher value. In addition, the randomness of the augmentation process, the choice of training/testing images, and the drop out operation during model training may cause small differences in precision.

**Fig 6:**
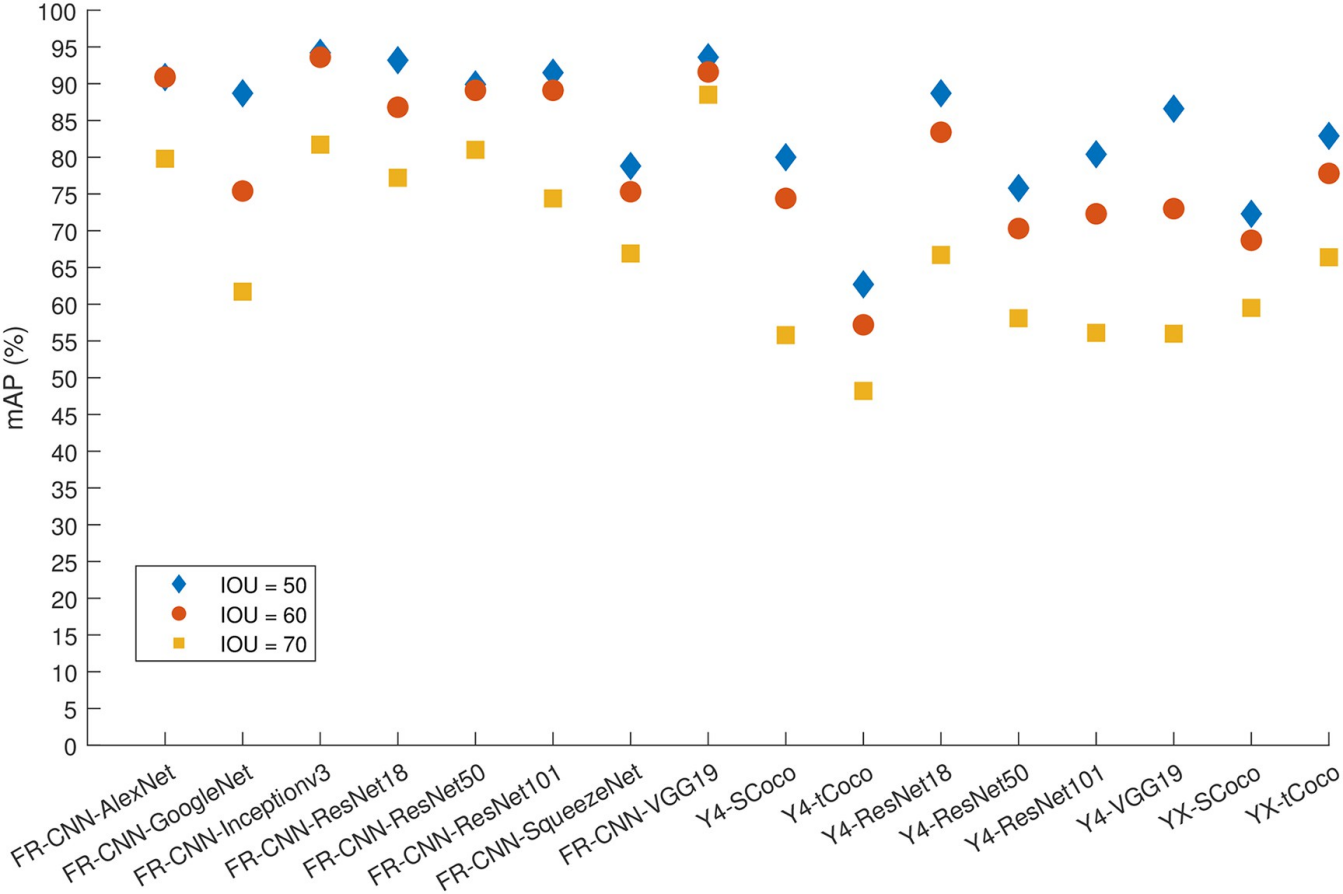
The mAP for all algorithms and 60% of the data used for training.

**Fig 7:**
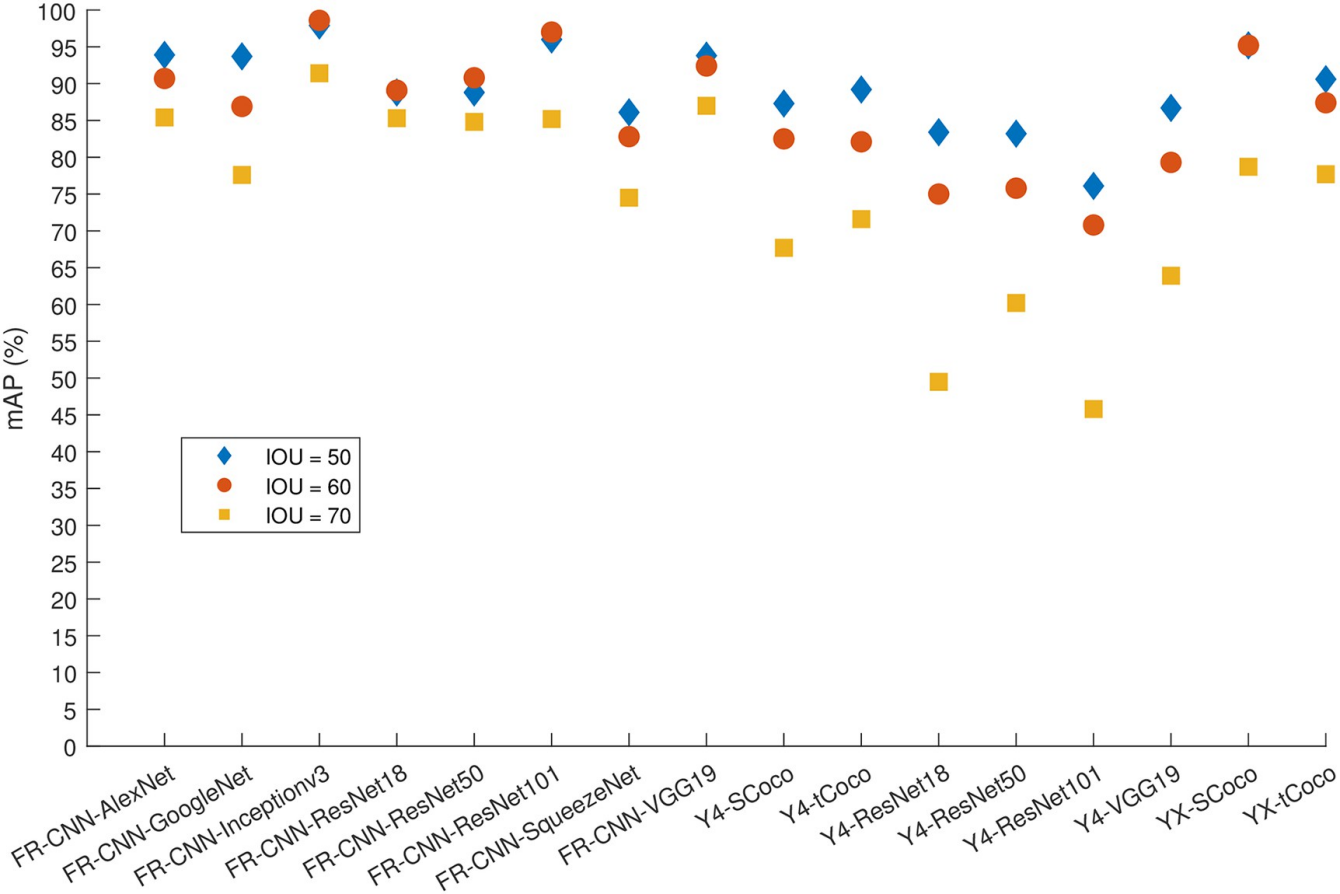
The mAP for all algorithms and 80% of the data used for training.

Further insight into the models is provided by Figs 6 and 7 and S1 and S2 Tables, which display the AP and mAP for three detectors and the two classes of EEG events using 60% and 80% of the data for training, respectively. A 10% increase in the training data has improved the precision of K-complex detection for Faster R-CNN by about 5%-16%, with the sole exception being the SqueezeNet model, which exhibited the least improvement in precision.

**Fig 8:**
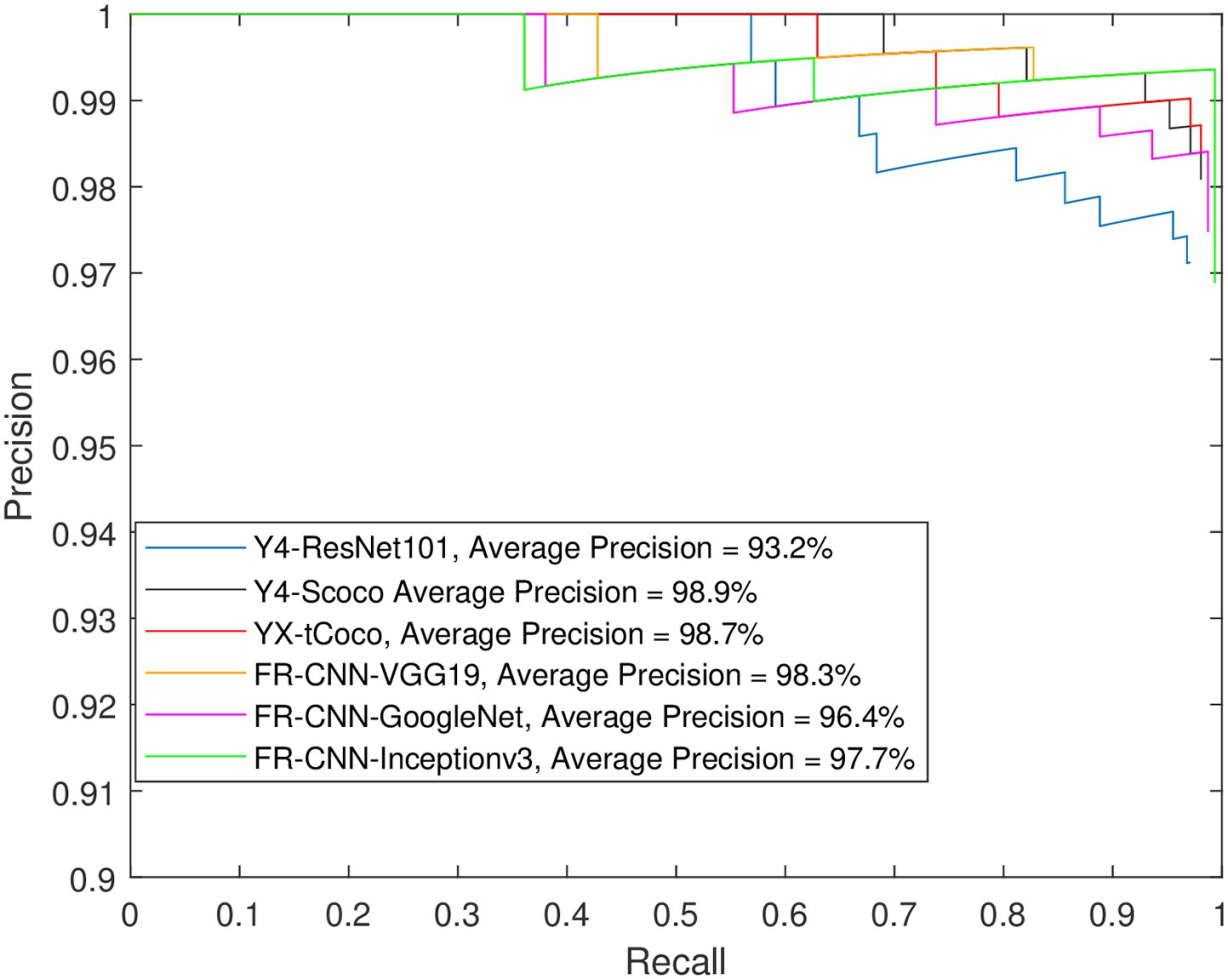
Testing object detection models built for sleep spindles only. The training data was 60% of each dataset and the IoU = 50.

**Fig 9:**
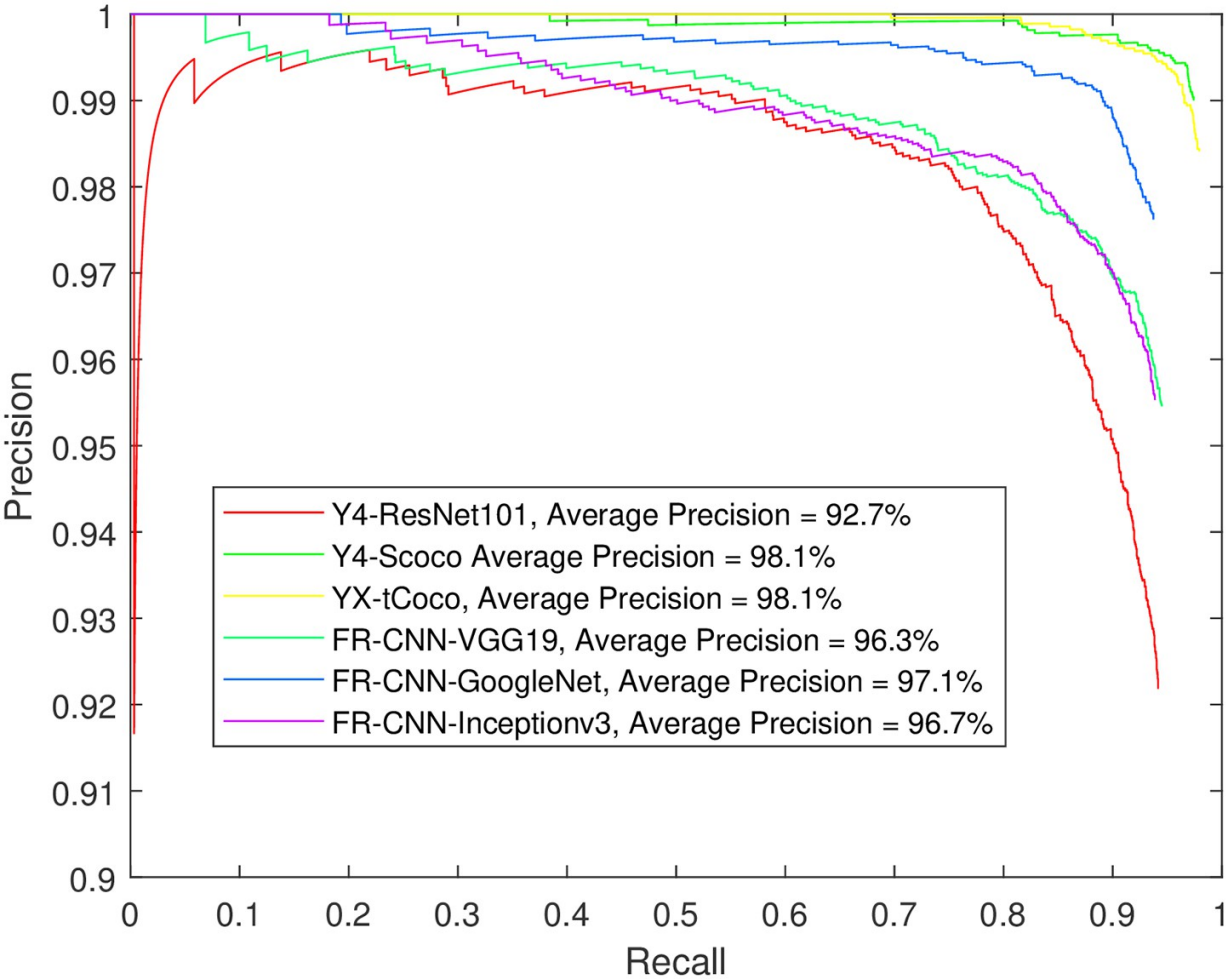
Testing object detection models built for K-complexes only. The training data was 60% of each dataset and the IoU = 50.

Furthermore, the extremely high precision for spindle detection brings the models closer to a plateau. Increasing the percentage of training images to 80% of the dataset seemed to significantly benefit many models in terms of K-complex detection. Notably, Faster R-CNN with Inceptionv3, GoogleNet or ResNet101 backbone, and YOLOX with a small Coco backbone were able to achieve an AP of more than 90% for IoU values of 50% and 60%. Achieving a 70% IoU appears to be challenging for most models, except for those using Inceptionv3 or VGG19, which were able to achieve an AP around 80%. On the other hand, YOLOv4 produced much lower precision regardless of the setup. In contrast, YOLOX improved greatly when 80% of the data used for training. The aforementioned precision numbers are further corroborated by S1 Fig through S48 through, which show the precision-recall curves for the Faster R-CNN, YOLOV4, and YOLOX detectors, respectively, for the sleep spindle and K-complex EEG events using an increasing training data proportion (from 50% to 80% of the dataset) and an increasing overlap threshold (50% to 70% IoU). Such performance figures are very close to and comparable with the results obtained from using individual models for each event type (see Figs 8 and 9).

The robustness of the models and core results are further demonstrated by the 5-fold cross-validation results shown in Table 1. Throughout the folds, IoU thresholds, and EEG events, the event detector using Faster R-CNN performed exceptionally well. It was only in one fold (i.e., fold 2 for SS and fold 3 for KC) and using a high IoU of 70% that the AP for spindles and K-complex detection dropped significantly, which led to an increase in the standard deviation in that specific case. The other entries showed much less variation or degradation in performance, although the K-complex detection was more affected by higher IoU values.

**Table 1:** The average precision (AP) and mean average precision (mAP) for the two classes of waveform patterns using 5-fold cross validation. KC: K-complex, SS: sleep spindle, SD: standard deviation.

		Fold 1	Fold 2	Fold 3	Fold 4	Fold 5	Folds Average	SD
**IOU = 50**	**SS**	98.7%	96.2%	99.0%	96.1%	99.4%	97.9%	1.4%
	**KC**	96.3%	100.0%	98.1%	100.0%	94.2%	97.7%	2.2%
**IOU = 60**	**SS**	97.8%	98.0%	99.5%	93.4%	99.2%	97.6%	2.2%
	**KC**	91.0%	99.2%	94.8%	99.4%	93.7%	95.6%	3.3%
**IOU = 70**	**SS**	98.0%	66.9%	98.3%	96.0%	95.0%	90.8%	12.0%
	**KC**	87.5%	92.8%	66.0%	98.7%	87.3%	86.5%	11.0%
	**mAP**	94.9%	92.2%	92.6%	97.3%	94.8%	94.3%	

**Fig 10:**
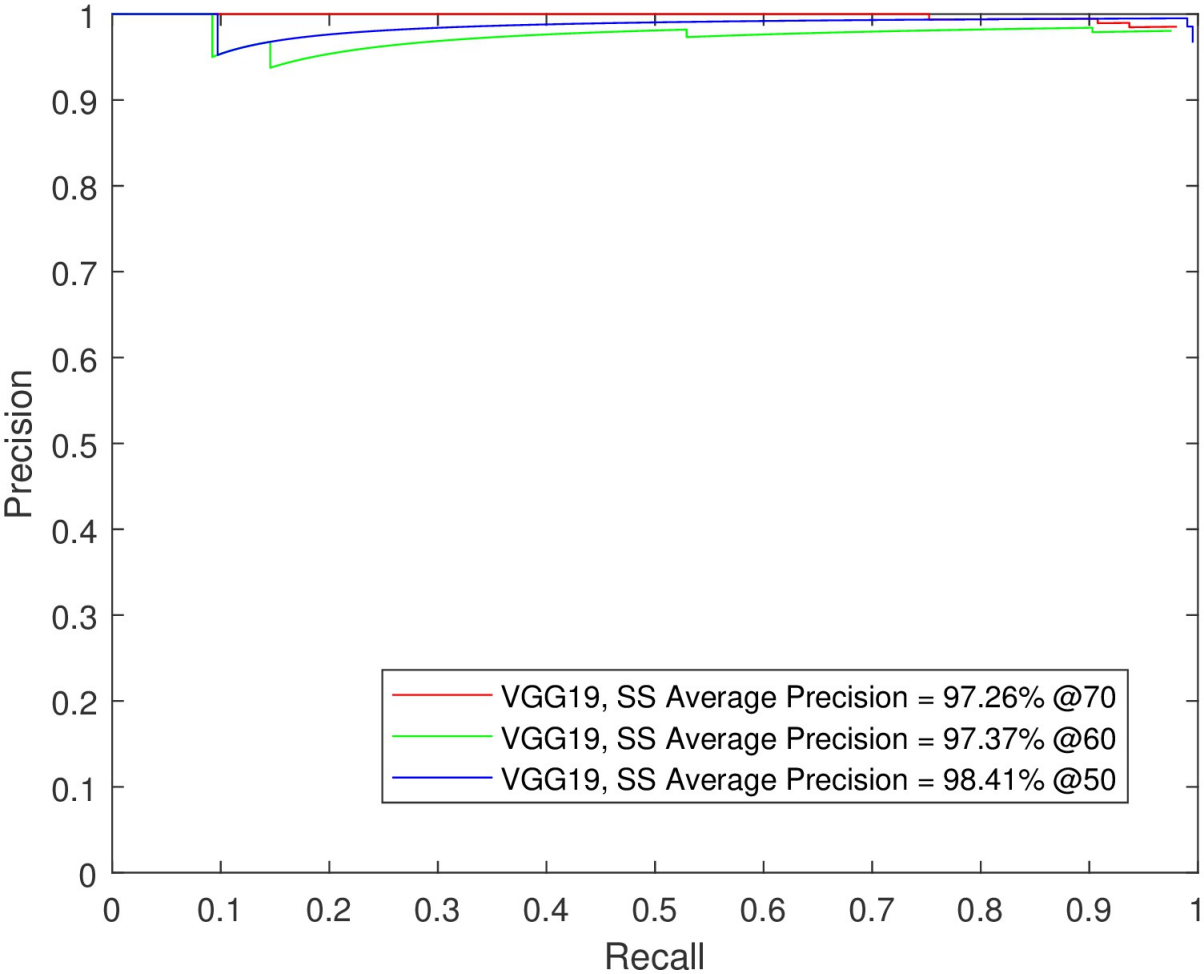
Testing Faster R-CNN using VGG19 for sleep spindles detection on data from independent subjects not seen during training. The number of testing images was 20% of the dataset.

**Fig 11:**
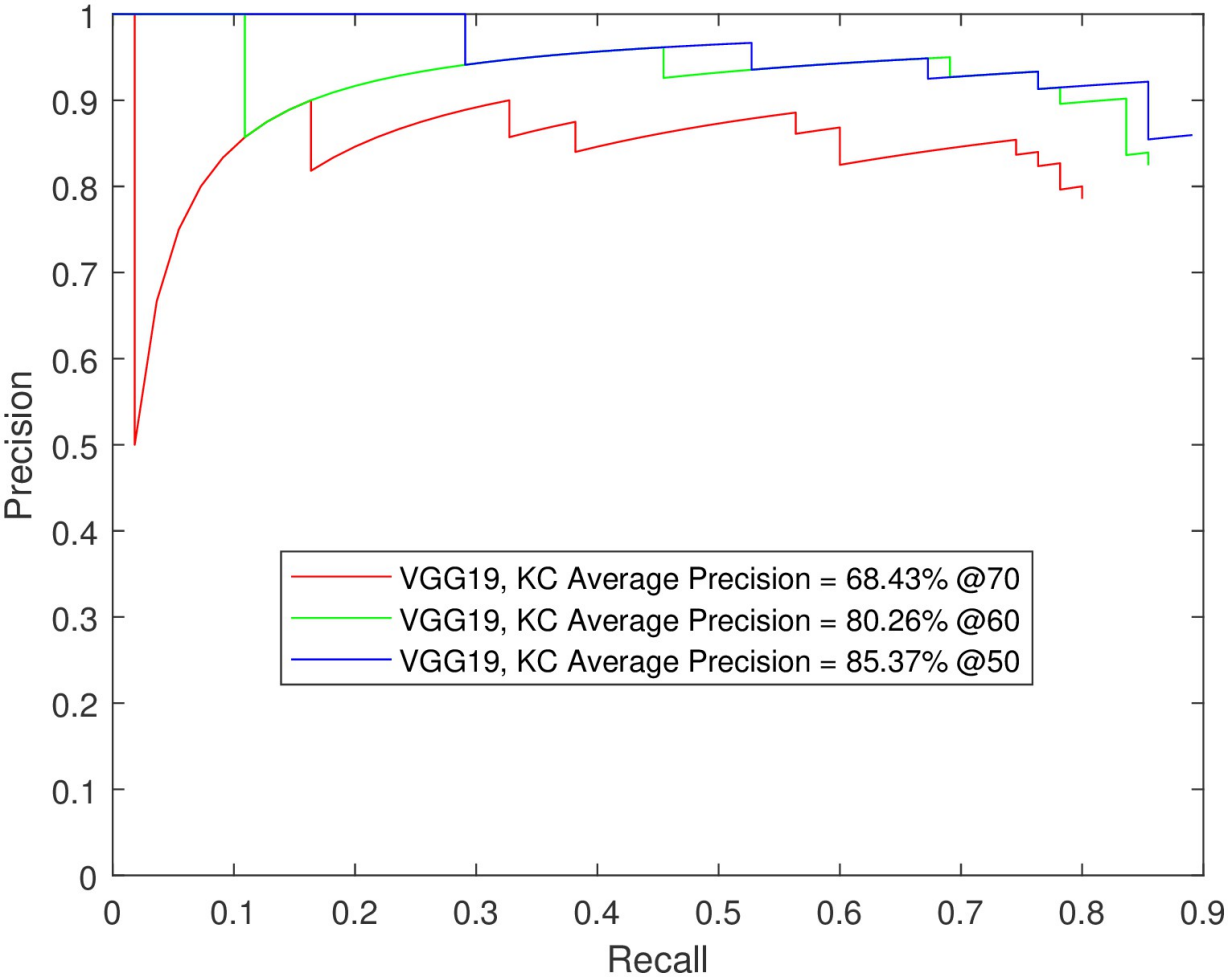
Testing Faster R-CNN using VGG19 K-complexes detection on data from independent subjects not seen during training. The number of testing images was 20% of the dataset.

In another demonstration of the detectors’ prowess, Faster R-CNN using VGG-19 was tested using data from independent subjects not seen during training. The precision-recall curves for the K-complexes and sleep spindles are shown in Figs 10 and 11. The figures follow the trend in the supporting tables, showing higher spindle detection performance compared to K-complexes (mAP of 85.4% vs. 98.4% @50).

One could attribute the difference in performance between spindles and K-complex detection precision to the imbalance caused by having more spindle images, which could result in ‘titling’ the model toward spindle detection. To investigate this, further analysis was conducted in connection to the imbalance in the number of images amongst the two classes in the dataset using some representative samples of the detector-CNN combinations. The models investigated were: Faster R-CNN using AlexNet, ResNet18, or VGG19, YOLOX using Tiny Coco, and YOLOv4 using Small Coco. Two approaches were followed for data balancing: First, the number of sleep spindle images was randomly reduced to match that of the K-complex images. Second, the number of K-complex images was increased via augmentation to match that of the sleep spindle images.

**Fig 12:**
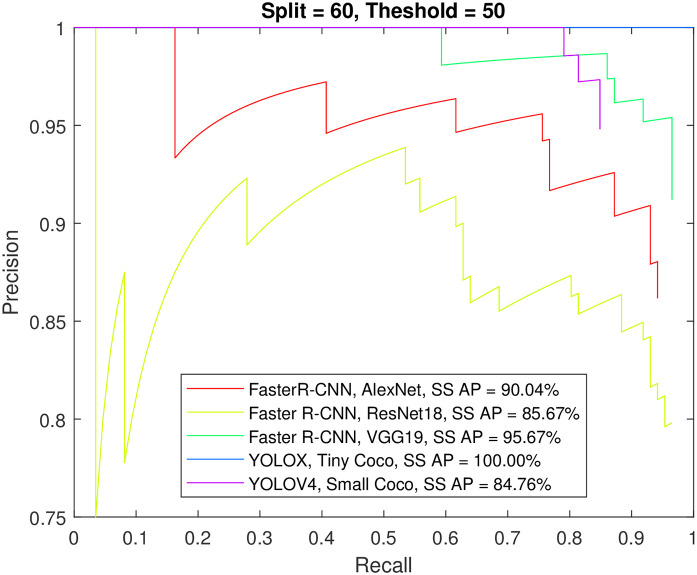
The precision-recall curve for sleep spindles detection when the detectors trained on a balanced dataset with 271 images for each class, and 60% of the data used for training and 50% overlap threshold.

**Fig 13:**
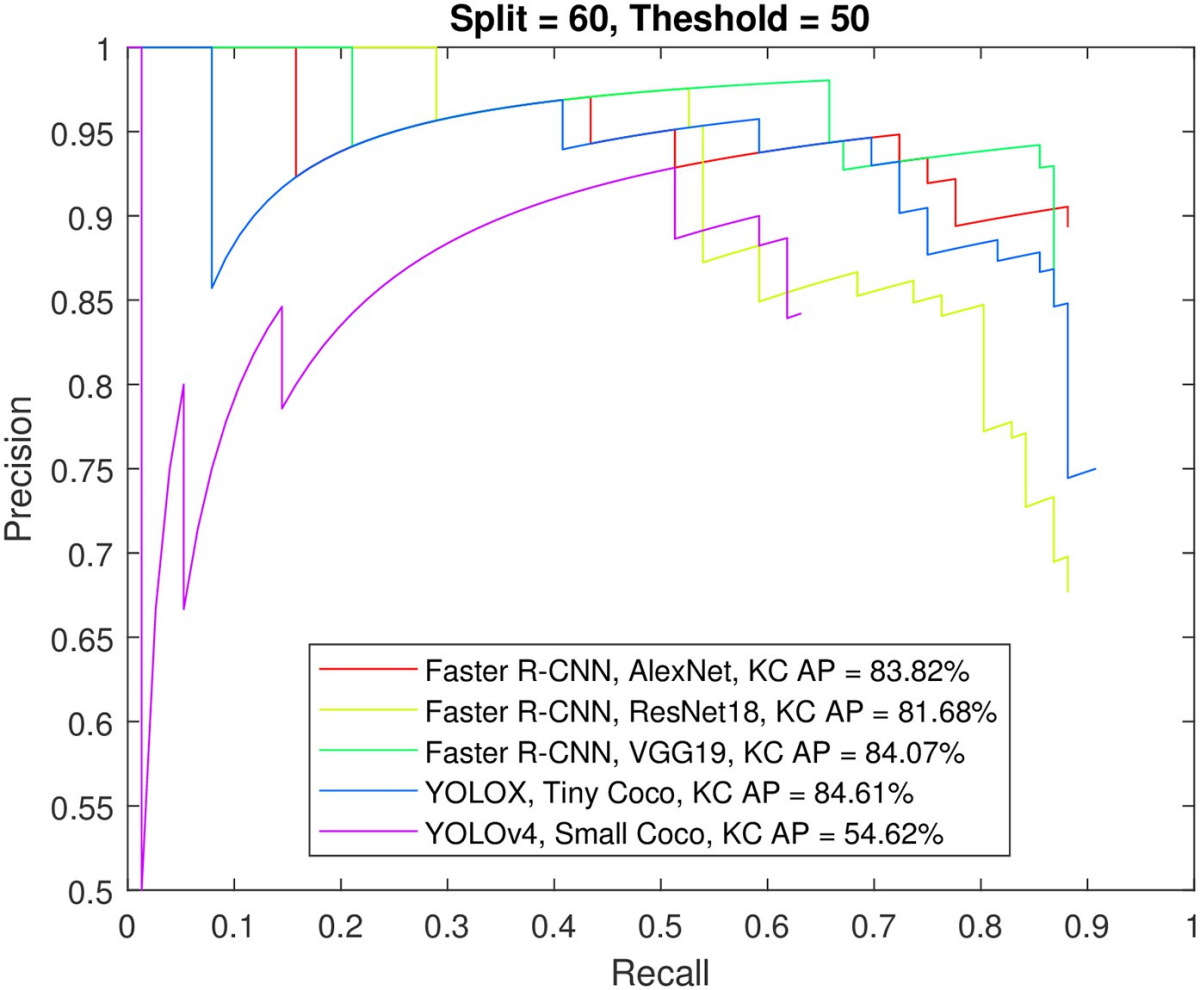
The precision-recall curve for K-complexes detection when the detectors trained on a balanced dataset with 271 images for each class, and 60% of the data used for training and 50% overlap threshold.

**Fig 14:**
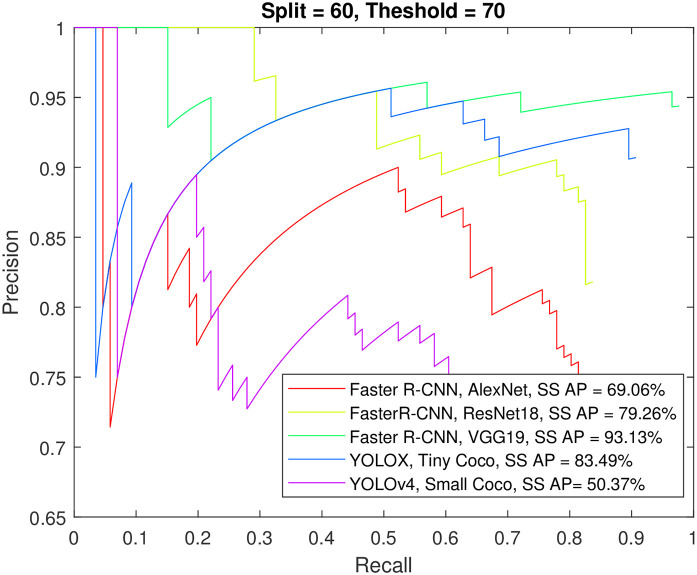
The precision-recall curve for sleep spindles detection when the detectors trained on a balanced dataset with 271 images for each class, and 60% of the data used for training and 70% overlap threshold.

**Fig 15:**
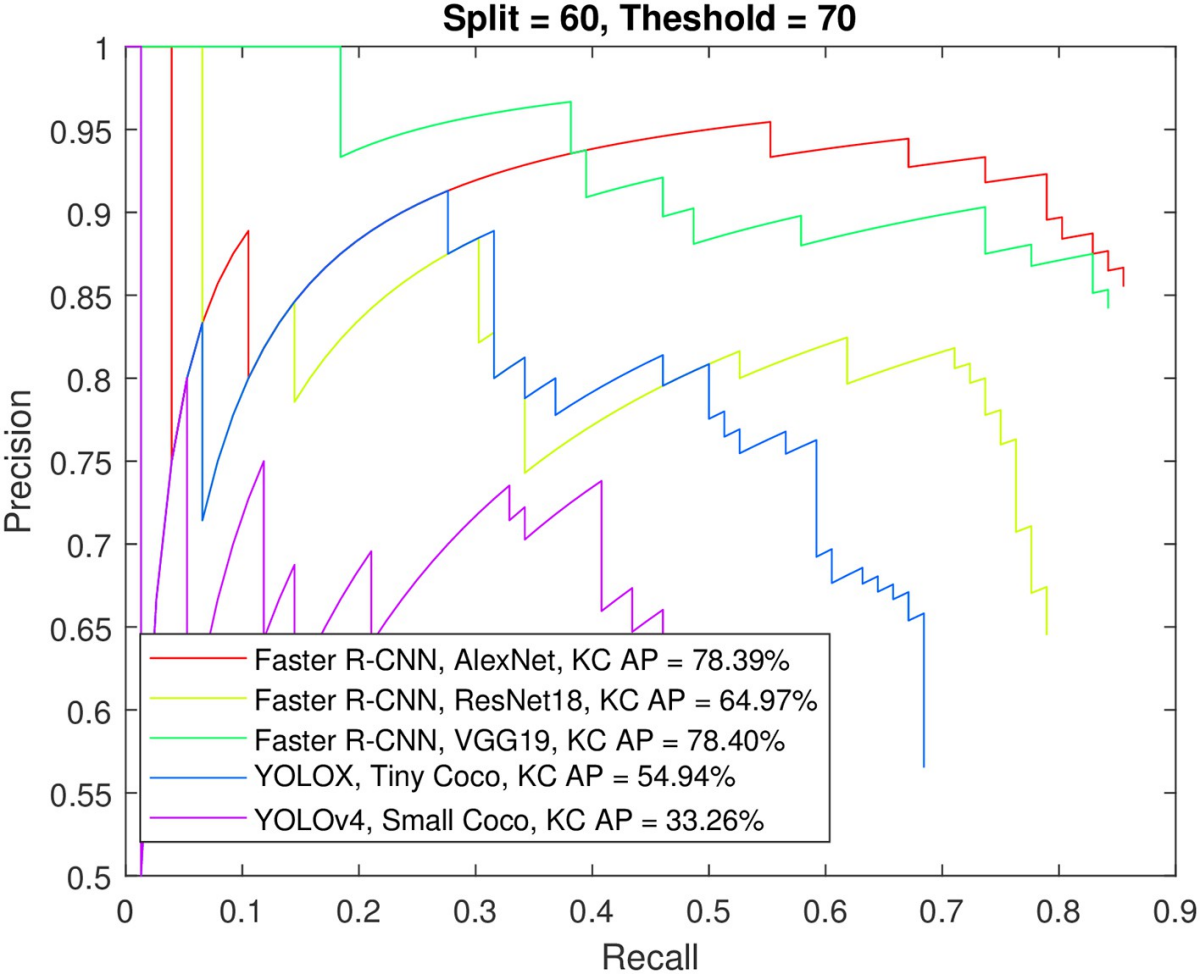
The precision-recall curve for K-complexes detection when the detectors trained on a balanced dataset with 271 images for each class, and 60% of the data used for training and 70% overlap threshold.

**Fig 16:**
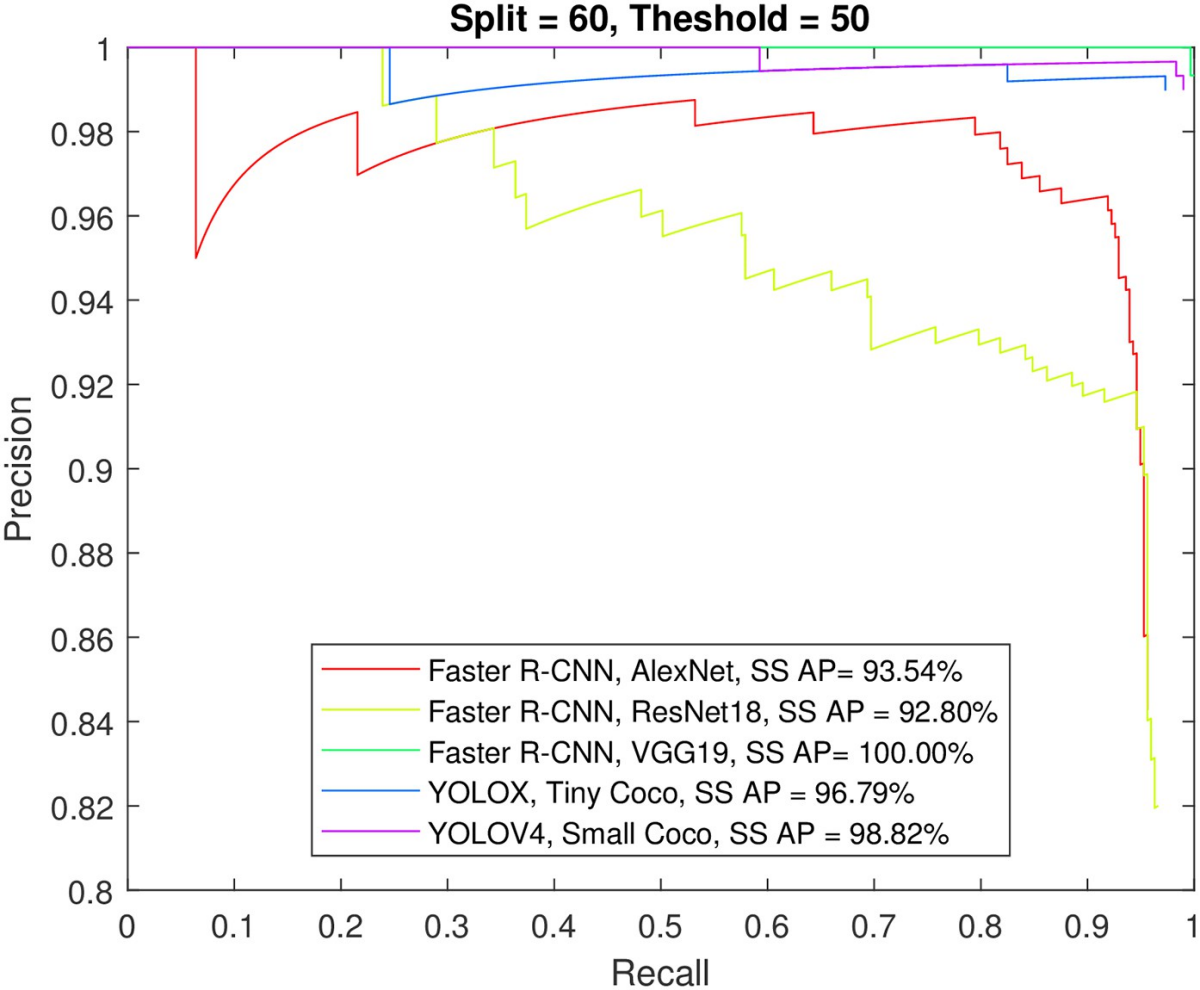
The precision-recall curve for sleep spindles detection when the detectors trained on a balanced dataset with 1044 images for each class, and 60% of the data used for training and 50% overlap threshold.

**Fig 17:**
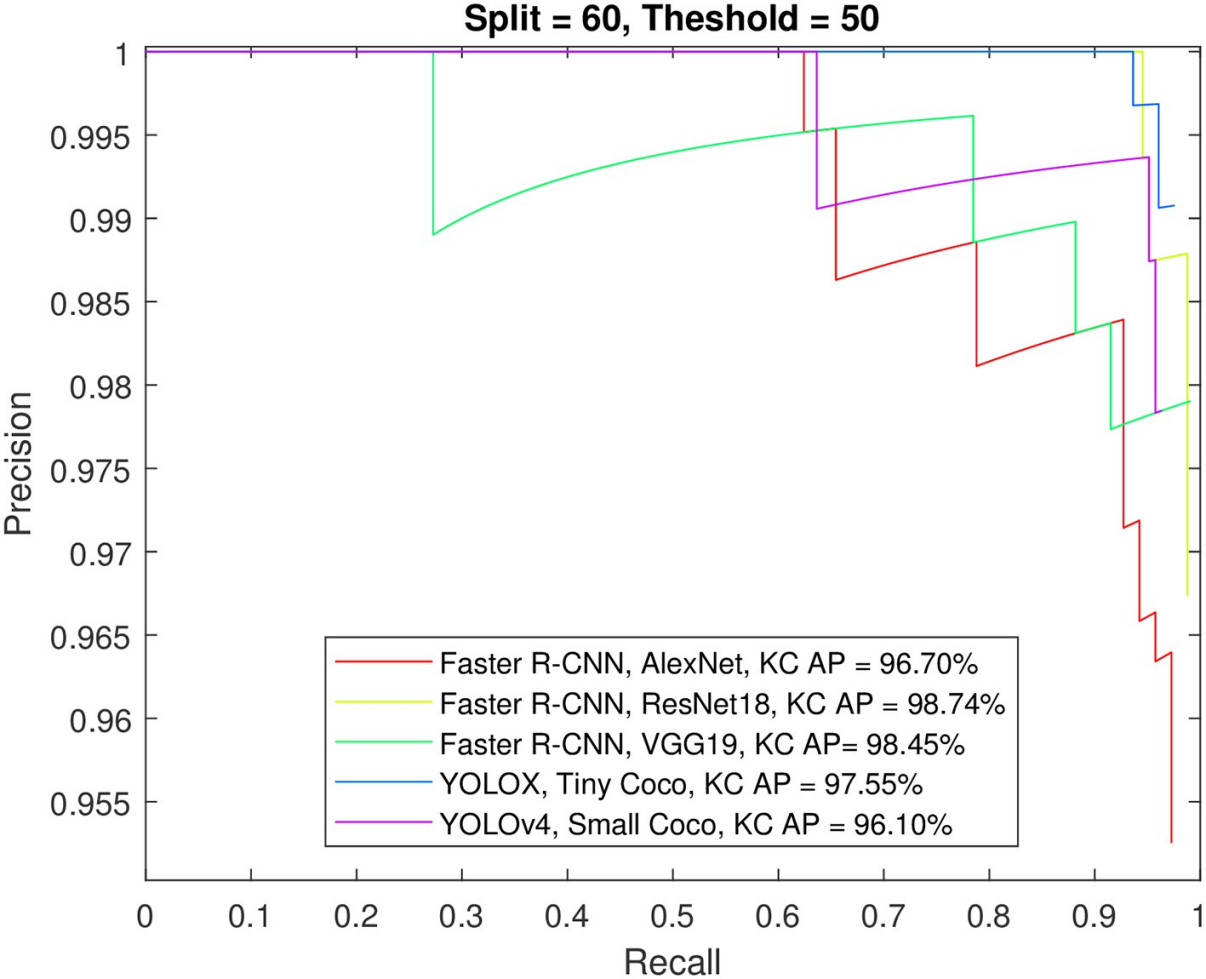
The precision-recall curve for K-complexes detection when the detectors trained on a balanced dataset with 1044 images for each class, and 60% of the data used for training and 50% overlap threshold.

**Fig 18:**
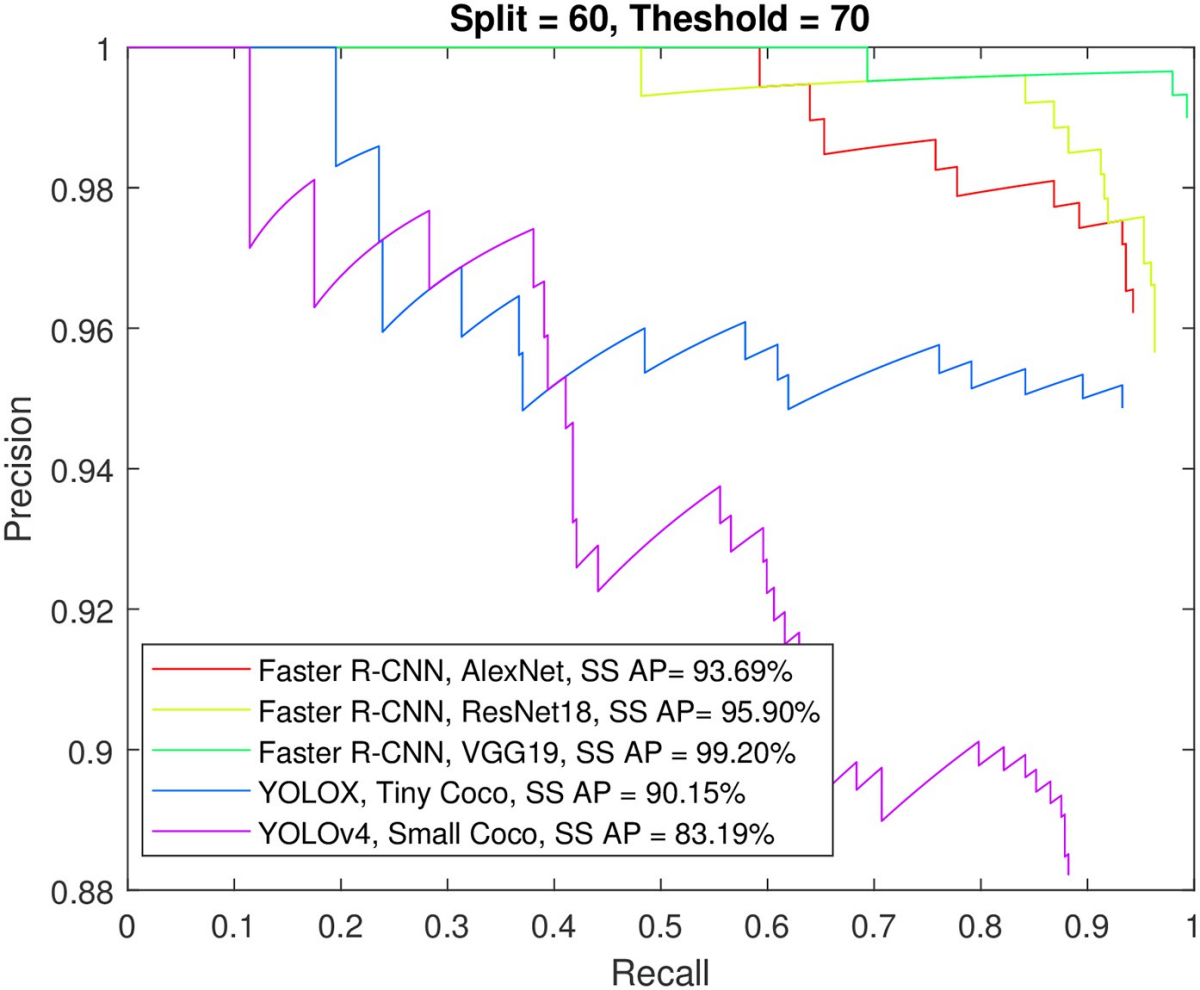
The precision-recall curve for sleep spindles detection when the detectors trained on a balanced dataset with 1044 images for each class, and 60% of the data used for training and 70% overlap threshold.

**Fig 19:**
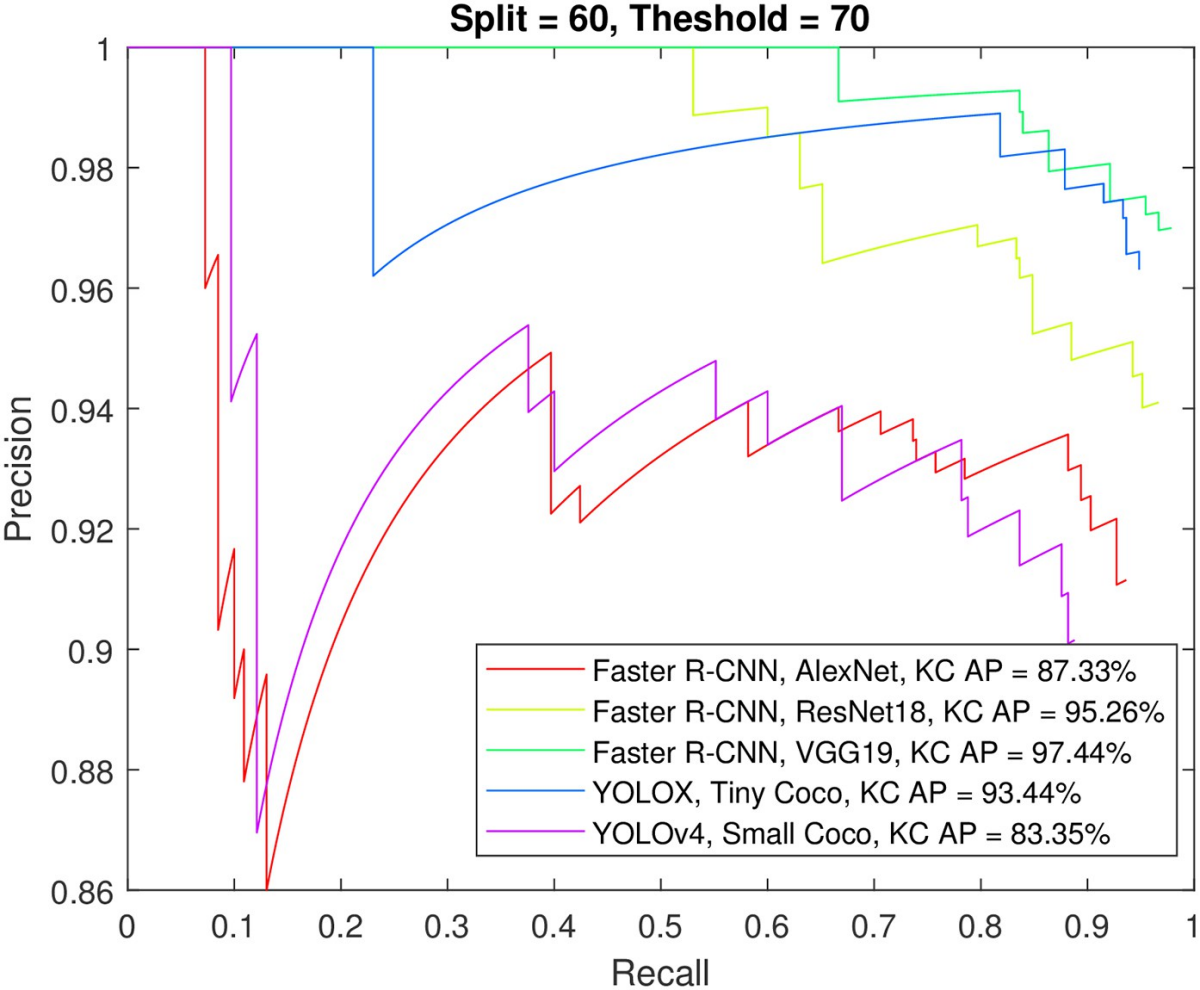
The precision-recall curve for K-complexes detection when the detectors trained on a balanced dataset with 1044 images for each class, and 60% of the data used for training and 70% overlap threshold.

Figs 12 to 15 show the precision-recall curve and AP for all detectors when trained on a balanced dataset with 271 images for each class, 60% of the data used for training, and 50%/70% overlap thresholds. The results in the figures indicate that balancing the dataset in this way has a more detrimental effect on spindle detection compared to its benefit on K-complex detection. On the other hand, Figs 16 to 19 show the precision-recall curve and AP for all detectors when trained on a balanced dataset with 1044 images for each class. The precision is exceptional for both classes. However, we do not believe that such results reflect the true precision. This is because deep learning algorithms are competent in detecting the small differences injected into the copies via augmentation, which in essence means that the models were tested on data seen before (i.e., during training). Such results maybe be taken as a form of upper limit on what can be achieved rather than true performance.

Table 2 presents the training times in minutes using 50% of the unbalanced dataset and 30 epochs. Overall, training with Faster R-CNN was the slowest. Compared to YOLOv4 with the same backbones, Faster R-CNN typically required almost five times more time for training. Another notable observation from the table is that when using YOLOX, the training times with Small Coco or Tiny Coco datasets were nearly identical. Additionally, YOLOX demonstrated significantly faster training speeds compared to YOLOv4 using the Small Coco backbone. As for inference times (not displayed), all models exhibited comparable speed, processing images in just a few milliseconds each (<15*ms*).

**Table 2:** The training times using 50% of the unbalanced dataset (in minutes) and 30 epochs.

Detector	Backbone	Training Time (min)
**Faster R-CNN**	AlexNet	17.1
	GoogleNet	199.1
	Inceptionv3	384.4
	ResNet18	71.4
	ResNet50	181.6
	ResNet101	201.8
	SqueezeNet	285.2
	VGG19	25.9
**YOLOv4**	Small Coco	483.9
	Tiny Coco	19.4
	ResNet18	18
	ResNet50	37.5
	ResNet101	58.0
	VGG19	11.7
**YOLOX**	Small Coco	81.3
	Tiny Coco	81.5

### Limitations and future work

This paper presents an alternative approach to a typical signal processing and feature extraction problem, an area that, despite extensive research, still offers significant potential for improvement. While the models demonstrated exceptional performance on unseen testing images from the same dataset, several limitations were identified. First, the models struggled considerably and failed to accurately detect spindles and K-complexes when tested on data from other unseen datasets. Second, neither dataset provides information about the reference scheme used in the recordings. This is important because the models are likely to perform well only with the reference schemes they were trained on (e.g., common reference, average reference, bipolar, linked ears) but may not generalize to others. This represents a promising area for future research. Third, the impact of this work could be further enhanced by extending the models or developing detectors for epileptiform events (e.g., spikes, sharps, temporal intermittent rhythmic delta activity (TIRDA) events). There is a substantial research gap in creating high-performing detectors for these events, which poses a significant challenge for advancing epilepsy and sleep-related research. Fourth, the models need to be integrated into a user-friendly application (e.g., a smartphone camera-based app) that can be easily utilized in clinical settings, making the technology more accessible and practical for healthcare professionals.

## Conclusion

The importance of EEG as a diagnostic tool is well-established in the field of medicine. Special events and waveforms in EEG recordings (e.g., sleep spindles and K-complexes) provide significant clues to specialists about the function and health of the brain. Consequently, the detection and localization process of these events needs to be performed accurately and quickly. To this end, the work in this paper targeted the identification and localization of sleep spindles and K-complexes in EEG waveform images. The approach reported here departs from the classical method of signal analysis, feature extraction, and classification. Instead, it follows a computer vision methodology that visually inspects the waveform in search of the events of interest. Three object detection algorithms using various convolutional neural network architectures were built and extensively evaluated. It was shown that developing a combined single model for highly accurate detection and localization is feasible. However, similar to deep learning models, larger sets of training data will produce more robust and highly precise models.

## Supporting information

S1 Table The average precision (AP) and mean average precision (mAP) for the three detectors and the two classes of waveform patterns using 50% of the data for training. KC stands for K-complex and SS stands for sleep spindle. The superscript in the mAP metric is for the IoU threshold.
(PDF)

S2 Table The average precision (AP) and mean average precision (mAP) for the three detectors and the two classes of waveform patterns using 60% of the data for training. KC stands for K-complex and SS stands for sleep spindle. The superscript in the mAP metric is for the IoU threshold.
(PDF)

S3 Table The average precision (AP) and mean average precision (mAP) for the three detectors and the two classes of waveform patterns using 80% of the data for training. KC stands for K-complex and SS stands for sleep spindle. The superscript in the mAP metric is for the IoU threshold.
(PDF)

S1 Fig Precision-recall curve for K-complex detection using Faster R-CNN and training data percentage = 50% and IoU = 50. (PDF)

S2 Fig Precision-recall curve for K-complex detection using Faster R-CNN and training data percentage = 50% and IoU = 70. (PDF)

S3 Fig Precision-recall curve for K-complex detection using Faster R-CNN and training data percentage = 60% and IoU = 50. (PDF)

S4 Fig Precision-recall curve for K-complex detection using Faster R-CNN and training data percentage = 60% and IoU = 70. (PDF)

S5 Fig Precision-recall curve for K-complex detection using Faster R-CNN and training data percentage = 70% and IoU = 50. (PDF)

S6 Fig Precision-recall curve for K-complex detection using Faster R-CNN and training data percentage = 70% and IoU = 70. (PDF)

S7 Fig Precision-recall curve for K-complex detection using Faster R-CNN and training data percentage = 80% and IoU = 50. (PDF)

S8 Fig Precision-recall curve for K-complex detection using Faster R-CNN and training data percentage = 80% and IoU = 70. (PDF)

S9 Fig Precision-recall curve for K-complex detection using YOLO4 and training data percentage = 50% and IoU = 50. (PDF)

S10 Fig Precision-recall curve for K-complex detection using YOLO4 and training data percentage = 50% and IoU = 70. (PDF)

S11 Fig Precision-recall curve for K-complex detection using YOLO4 and training data percentage = 60% and IoU = 50. (PDF)

S12 Fig Precision-recall curve for K-complex detection using YOLO4 and training data percentage = 60% and IoU = 70. (PDF)

S13 Fig Precision-recall curve for K-complex detection using YOLO4 and training data percentage = 70% and IoU = 50. (PDF)

S14 Fig Precision-recall curve for K-complex detection using YOLO4 and training data percentage = 70% and IoU = 70. (PDF)

S15 Fig Precision-recall curve for K-complex detection using YOLO4 and training data percentage = 80% and IoU = 50. (PDF)

S16 Fig Precision-recall curve for K-complex detection using YOLO4 and training data percentage = 80% and IoU = 70. (PDF)

S17 Fig Precision-recall curve for K-complex detection using YOLOX and training data percentage = 50% and IoU = 50. (PDF)

S18 Fig Precision-recall curve for K-complex detection using YOLOX and training data percentage = 50% and IoU = 70. (PDF)

S19 Fig Precision-recall curve for K-complex detection using YOLOX and training data percentage = 60% and IoU = 50. (PDF)

S20 Fig Precision-recall curve for K-complex detection using YOLOX and training data percentage = 60% and IoU = 70. (PDF)

S21 Fig Precision-recall curve for K-complex detection using YOLOX and training data percentage = 70% and IoU = 50. (PDF)

S22 Fig Precision-recall curve for K-complex detection using YOLOX and training data percentage = 70% and IoU = 70. (PDF)

S23 Fig Precision-recall curve for K-complex detection using YOLOX and training data percentage = 80% and IoU = 50. (PDF)

S24 Fig Precision-recall curve for K-complex detection using YOLOX and training data percentage = 80% and IoU = 70. (PDF)

S25 Fig Precision-recall curve for spindles detection using Faster R-CNN and training data percentage = 50% and IoU = 50. (PDF)

S26 Fig Precision-recall curve for spindles detection using Faster R-CNN and training data percentage = 50% and IoU = 70. (PDF)

S27 Fig Precision-recall curve for spindles detection using Faster R-CNN and training data percentage = 60% and IoU = 50. (PDF)

S28 Fig Precision-recall curve for spindles detection using Faster R-CNN and training data percentage = 60% and IoU = 70. (PDF)

S29 Fig Precision-recall curve for spindles detection using Faster R-CNN and training data percentage = 70% and IoU = 50. (PDF)

S30 Fig Precision-recall curve for spindles detection using Faster R-CNN and training data percentage = 70% and IoU = 70. (PDF)

S31 Fig Precision-recall curve for spindles detection using Faster R-CNN and training data percentage = 80% and IoU = 50. (PDF)

S32 Fig Precision-recall curve for spindles detection using Faster R-CNN and training data percentage = 80% and IoU = 70. (PDF)

S33 Fig Precision-recall curve for spindles detection using YOLO4 and training data percentage = 50% and IoU = 50. (PDF)

S34 Fig Precision-recall curve for spindles detection using YOLO4 and training data percentage = 50% and IoU = 70. (PDF)

S35 Fig Precision-recall curve for spindles detection using YOLO4 and training data percentage = 60% and IoU = 50. (PDF)

S36 Fig Precision-recall curve for spindles detection using YOLO4 and training data percentage = 60% and IoU = 70. (PDF)

S37 Fig Precision-recall curve for spindles detection using YOLO4 and training data percentage = 70% and IoU = 50. (PDF)

S38 Fig Precision-recall curve for spindles detection using YOLO4 and training data percentage = 70% and IoU = 70. (PDF)

S39 Fig Precision-recall curve for spindles detection using YOLO4 and training data percentage = 80% and IoU = 50. (PDF)

S40 Fig Precision-recall curve for spindles detection using YOLO4 and training data percentage = 80% and IoU = 70. (PDF)

S41 Fig Precision-recall curve for spindles detection using YOLOX and training data percentage = 50% and IoU = 50. (PDF)

S42 Fig Precision-recall curve for spindles detection using YOLOX and training data percentage = 50% and IoU = 70. (PDF)

S43 Fig Precision-recall curve for spindles detection using YOLOX and training data percentage = 60% and IoU = 50. (PDF)

S44 Fig Precision-recall curve for spindles detection using YOLOX and training data percentage = 60% and IoU = 70. (PDF)

S45 Fig Precision-recall curve for spindles detection using YOLOX and training data percentage = 70% and IoU = 50. (PDF)

S46 Fig Precision-recall curve for spindles detection using YOLOX and training data percentage = 70% and IoU = 70. (PDF)

S47 Fig Precision-recall curve for spindles detection using YOLOX and training data percentage = 80% and IoU = 50. (PDF)

S48 Fig Precision-recall curve for spindles detection using YOLOX and training data percentage = 80% and IoU = 70. (PDF)
