# Supplementary material for: Detection and location of EEG events using deep learning visual inspection
Source: PLoS One. 2024 Dec 23;19(12):e0312763. doi: 10.1371/journal.pone.0312763 (PMC11666049; doi:10.1371/journal.pone.0312763)
Supplement: S1 Table — KC stands for K-complex and SS stands for sleep spindle. The superscript in the mAP metric is for the IoU threshold. (PDF) [file pone.0312763.s001.pdf]

S1 Table. The average precision (AP) and mean average precision (mAP) for the three detectors and the two classes of waveform patterns using 50% of the data for training. KC stands for K-complex and SS stands for sleep spindle. The superscript in the mAP abbreviation is for the IoU threshold.

**Detector**	**Backbone**	AP_{KC}^{50}	AP_{SS}^{50}	mAP^{50}	AP_{KC}^{60}	AP_{SS}^{60}	mAP^{60}	AP_{KC}^{70}	AP_{SS}^{70}	mAP^{70}	**mAP**
**Faster R-CNN**	AlexNet	78.2%	91.3%	84.8%	77.9%	96.8%	87.4%	75.0%	93.5%	84.3%	85.5%
	GoogleNet	74.1%	97.2%	85.7%	52.8%	96.2%	74.5%	27.6%	94.9%	61.3%	73.8%
	Inceptionv3	85.5%	99.2%	92.4%	77.5%	98.8%	88.2%	71.1%	97.5%	84.3%	88.3%
	ResNet18	73.0%	95.3%	84.2%	55.6%	98.2%	76.9%	64.4%	92.4%	78.4%	79.8%
	ResNet50	71.5%	99.0%	85.3%	74.7%	97.0%	85.9%	71.2%	97.2%	84.2%	85.1%
	ResNet101	73.8%	97.6%	85.7%	74.3%	98.9%	86.6%	69.1%	95.5%	82.3%	84.9%
	SqueezeNet	69.0%	85.5%	77.3%	64.9%	85.0%	75.0%	53.5%	66.8%	60.2%	70.8%
	VGG19	88.2%	98.2%	93.2%	79.4%	91.4%	85.4%	67.7%	97.9%	82.8%	87.1%
**YOLOv4**	Small Coco	50.6%	89.9%	70.3%	45.6%	71.2%	58.4%	31.5%	45.4%	38.45%	55.7%
	Tiny Coco	75.3%	95.0%	85.2%	69.4%	94.1%	81.8%	41.2%	85.3%	63.25%	76.7%
	ResNet18	66.0%	98.0%	82.0%	46.3%	93.6%	70.0%	33.9%	75.7%	54.8%	68.9%
	ResNet50	44.9%	97.8%	71.4%	34.1%	91.6%	62.9%	19.4%	79.2%	49.3%	61.2%
	ResNet101	51.3%	96.8%	74.1%	40.1%	84.3%	62.2%	23.3%	53.8%	38.55%	58.3%
	VGG19	76.9%	98.3%	87.6%	67.2%	92.8%	80.0%	36.4%	61.8%	49.1%	72.2%
**YOLOX**	Small Coco	66.9%	98.1%	82.5%	60.6%	96.2%	78.4%	44.0%	90.0%	67.0%	76.0%
	Tiny Coco	76.2%	98.2%	87.2%	57.5%	97.3%	77.4%	40.4%	92.8%	66.7%	77.1%
