# Supplementary material for: Detection and location of EEG events using deep learning visual inspection
Source: PLoS One. 2024 Dec 23;19(12):e0312763. doi: 10.1371/journal.pone.0312763 (PMC11666049; doi:10.1371/journal.pone.0312763)
Supplement: S2 Table — KC stands for K-complex and SS stands for sleep spindle. The superscript in the mAP metric is for the IoU threshold. (PDF) [file pone.0312763.s002.pdf]

S2 Table. The average precision (AP) and mean average precision (mAP) for the three detectors and the two classes of waveform patterns using 60% of the data for training. KC stands for K-complex and SS stands for sleep spindle. The superscript in the mAP metric is for the IoU threshold.

Detector	Backbone	AP^{50}_{KC}	AP^{50}_{SS}	mAP^{50}	AP^{60}_{KC}	AP^{60}_{SS}	mAP^{60}	AP^{70}_{KC}	AP^{70}_{SS}	mAP^{70}	mAP
**Faster R-CNN**	AlexNet	89.4%	92.3%	90.9%	86.7%	95.1%	90.9%	65.1%	94.4%	79.8%	87.2%
	GoogleNet	79.5%	97.9%	88.7%	51.9%	98.8%	75.4%	23.7%	99.7%	61.7%	75.3%
	Inceptionv3	88.7%	99.7%	94.2%	87.1%	100%	93.6%	66.8%	96.6%	81.7%	89.8%
	ResNet18	89.2%	97.2%	93.2%	82.7%	90.9%	86.8%	63.5%	90.9%	77.2%	85.7%
	ResNet50	81.1%	98.6%	89.9%	78.8%	99.4%	89.1%	63.8%	98.2%	81.0%	86.7%
	ResNet101	84.0%	99.0%	91.5%	80.7%	97.4%	89.1%	49.8%	98.9%	74.4%	85.0%
	SqueezeNet	68.0%	89.6%	78.8%	68.6%	82.0%	75.3%	61.8%	72.0%	66.9%	73.7%
	VGG19	89.1%	98.1%	93.6%	88.3%	94.8%	91.6%	84.2%	92.8%	88.5%	91.2%
**YOLOv4**	Small Coco	63.4%	96.6%	80.0%	55.3%	93.4%	74.4%	32.8%	78.8%	55.8%	70.1%
	Tiny Coco	29.5%	95.8%	62.7%	19%	95.4%	57.2%	8.29%	88.1%	48.2%	56.0%
	ResNet18	78.0%	99.4%	88.7%	69.7%	97.1%	83.4%	51.9%	81.4%	66.7%	79.6%
	ResNet50	52.3%	99.2%	75.8%	43.3%	97.3%	70.3%	27.2%	88.9%	58.1%	68.0%
	ResNet101	61.7%	99.0%	80.4%	48.3%	97.2%	72.3%	35.9%	76.2%	56.1%	69.7%
	VGG19	74.2%	99.0%	86.6%	51.8%	94.1%	73.0%	36.8%	75.2%	56.0%	71.9%
**YOLOX**	Small Coco	62.4%	82.2%	72.3%	56.9%	80.4%	68.7%	42.4%	76.6%	59.5%	66.8%
	Tiny Coco	66.1%	99.7%	82.9%	56.8%	98.8%	77.8%	38.9%	93.9%	66.4%	75.7%
