# Supplementary material for: Detection and location of EEG events using deep learning visual inspection
Source: PLoS One. 2024 Dec 23;19(12):e0312763. doi: 10.1371/journal.pone.0312763 (PMC11666049; doi:10.1371/journal.pone.0312763)
Supplement: S3 Table — KC stands for K-complex and SS stands for sleep spindle. The superscript in the mAP metric is for the IoU threshold. (PDF) [file pone.0312763.s003.pdf]

S3 Table. The average precision (AP) and mean average precision (mAP) for the three detectors and the two classes of waveform patterns using 80% of the data for training. KC stands for K-complex and SS stands for sleep spindle. The superscript in the mAP metric is for the IoU threshold.

Detector	Backbone	AP^{50}_{KC}	AP^{50}_{SS}	mAP^{50}	AP^{60}_{KC}	AP^{60}_{SS}	mAP^{60}	AP^{70}_{KC}	AP^{70}_{SS}	mAP^{70}	mAP
**Faster R-CNN**	AlexNet	88.9%	98.9%	93.9%	84.8%	96.5%	90.7%	76.4%	94.3%	85.4%	90.0%
	GoogleNet	90.4%	97.0%	93.7%	77.8%	96.0%	86.9%	59.8%	95.4%	77.6%	86.1%
	Inceptionv3	97.2%	98.6%	97.9%	97.1%	100%	98.6%	82.7%	100%	91.4%	95.9%
	ResNet18	88.3%	89.2%	88.8%	80.3%	97.8%	89.1%	71.7%	98.9%	85.3%	87.7%
	ResNet50	77.5%	100%	88.8%	81.6%	100%	90.8%	71.6%	97.9%	84.8%	88.1%
	ResNet101	95.7%	96.2%	96.0%	94.1%	99.8%	97.0%	73.2%	97.2%	85.2%	92.7%
	SqueezeNet	78.2%	94.0%	86.1%	79.3%	86.3%	82.8%	74.3%	74.7%	74.5%	81.1%
	VGG19	88.8%	98.8%	93.8%	85.9%	98.9%	92.4%	79.5%	94.4%	87.0%	91.1%
**YOLOv4**	Small Coco	74.6%	100%	87.3%	70.7%	94.2%	82.5%	51.9%	83.4%	67.7%	79.1%
	Tiny Coco	78.3%	100%	89.2%	64.3%	99.9%	82.1%	48.1%	95.1%	71.6%	81.0%
	ResNet18	66.8%	100%	83.4%	52.6%	97.3%	75.0%	19.8%	79.1%	49.5%	69.3%
	ResNet50	67.4%	99.0%	83.2%	52.5%	99.0%	75.8%	31.4%	88.9%	60.2%	73.0%
	ResNet101	60.0%	92.2%	76.1%	44.5%	97.0%	70.8%	11.9%	79.6%	45.8%	64.2%
	VGG19	75.4%	98.0%	86.7%	60.6%	97.9%	79.3%	49.6%	78.2%	63.9%	76.6%
**YOLOX**	Small Coco	91.3%	99.0%	95.2%	91.3%	99.0%	95.2%	63.7%	93.7%	78.7%	89.7%
	Tiny Coco	83.3%	97.9%	90.6%	76.9%	97.9%	87.4%	59.7%	95.6%	77.7%	85.2%
