# Supplementary figures and images for: Detection and location of EEG events using deep learning visual inspection
Source: PLoS One. 2024 Dec 23;19(12):e0312763. doi: 10.1371/journal.pone.0312763 (PMC11666049; doi:10.1371/journal.pone.0312763)

Split = 50, Theshold = 50

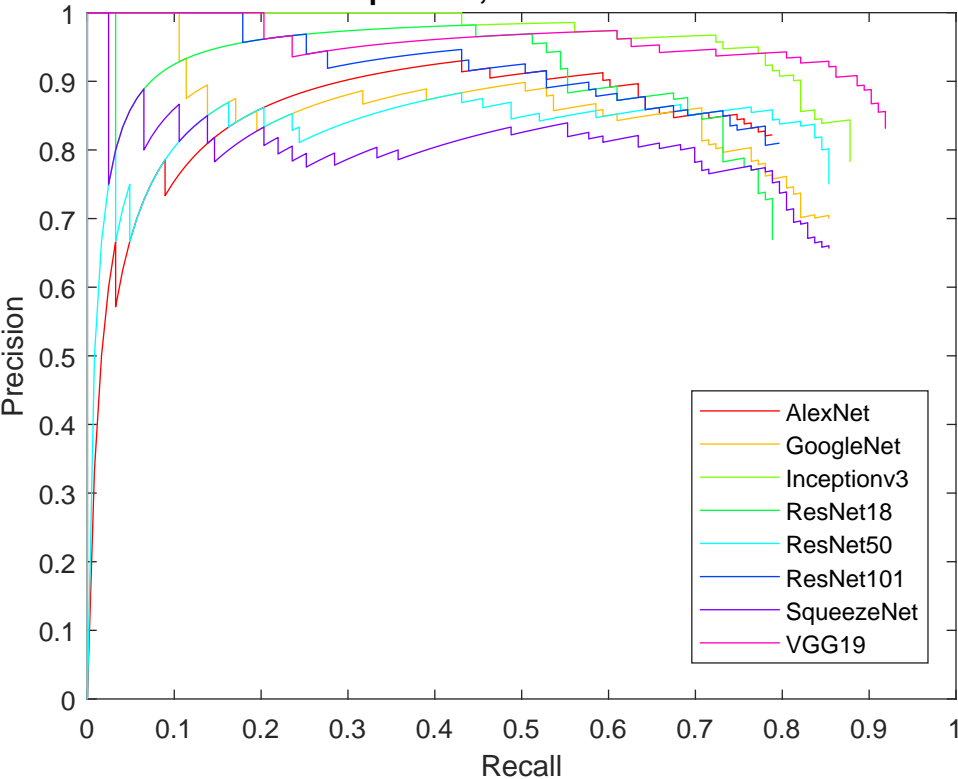

Supplement: S1 Fig — (PDF) [file pone.0312763.s004.pdf]

Split = 50, Theshold = 70

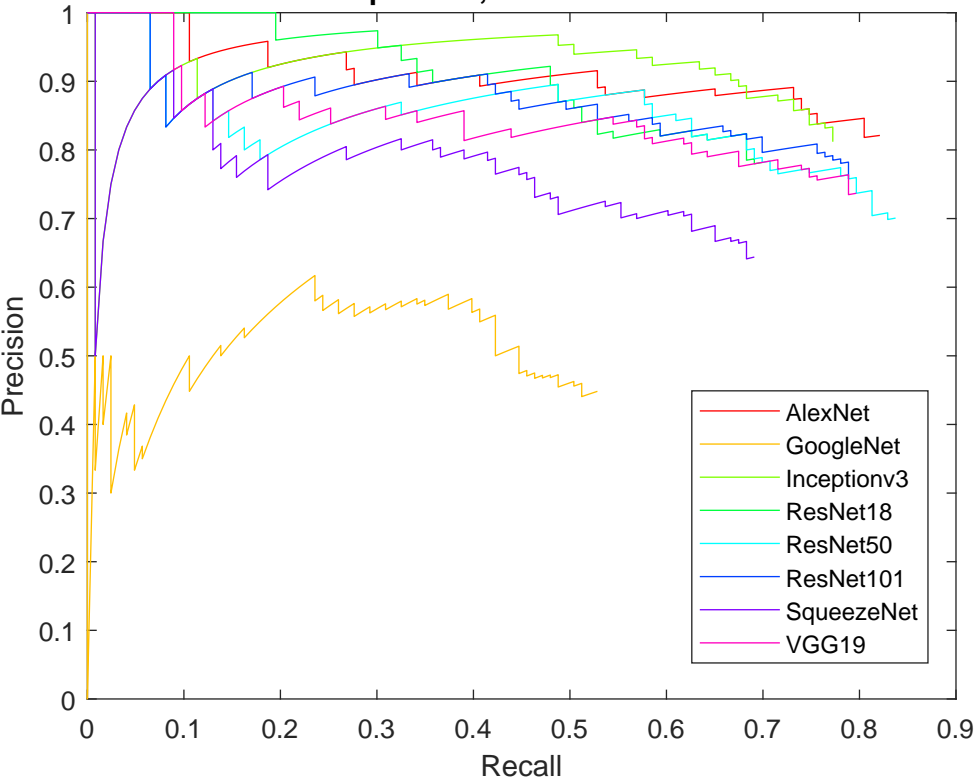

Supplement: S2 Fig — (PDF) [file pone.0312763.s005.pdf]

Split = 60, Theshold = 50

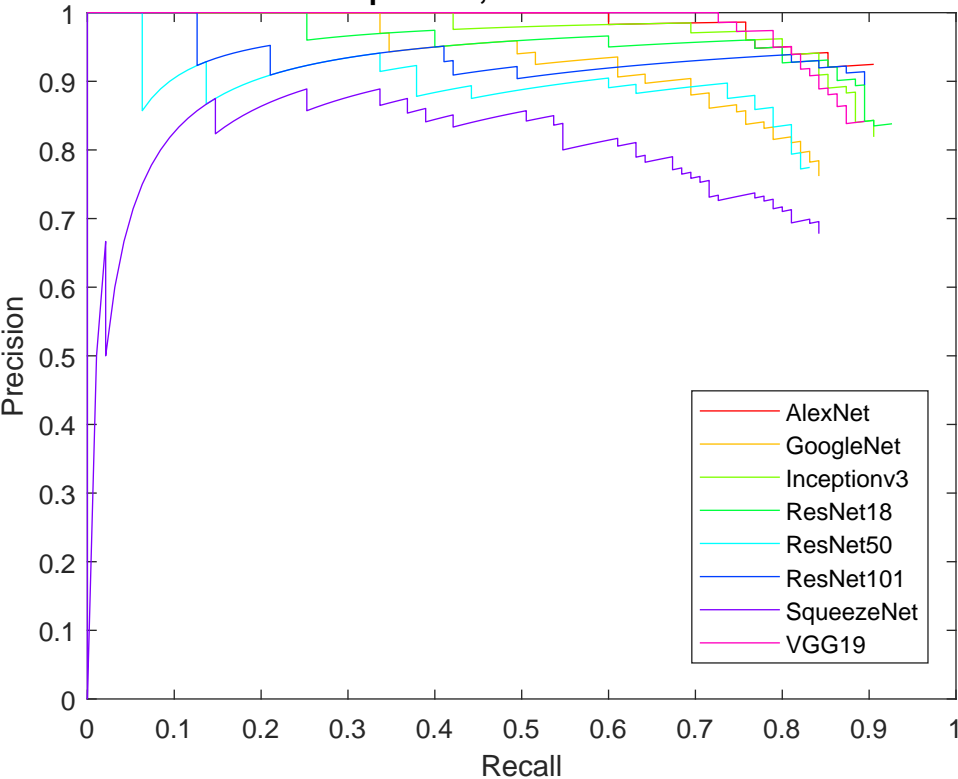

Supplement: S3 Fig — (PDF) [file pone.0312763.s006.pdf]

**Split = 60, Theshold = 70**

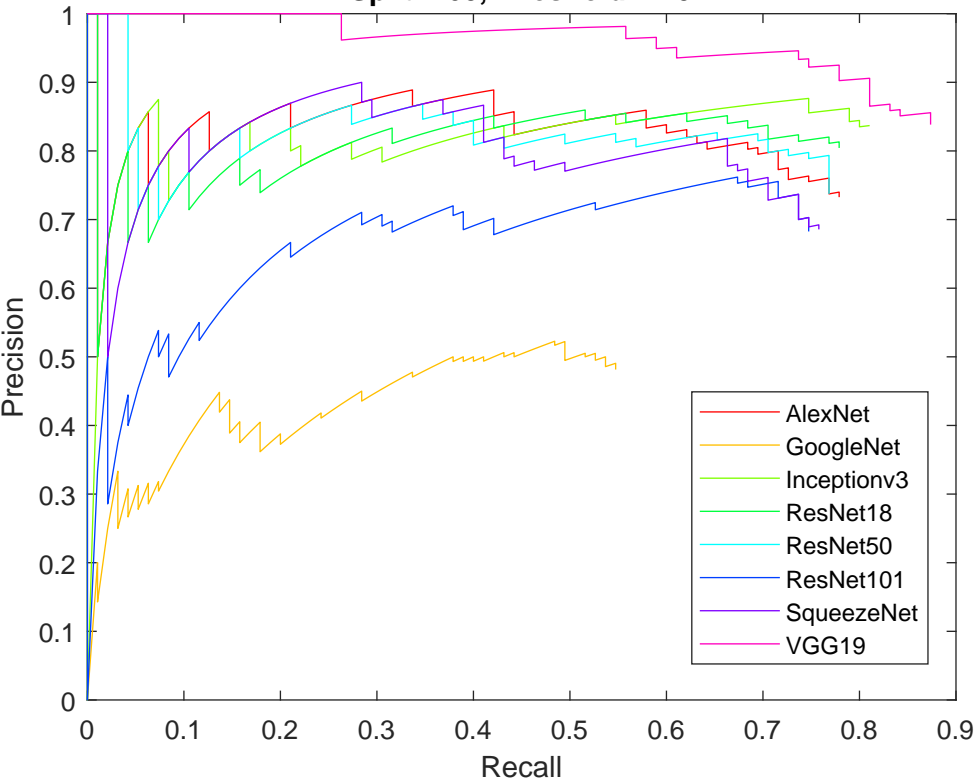

Supplement: S4 Fig — (PDF) [file pone.0312763.s007.pdf]

Split = 70, Theshold = 50

Precision

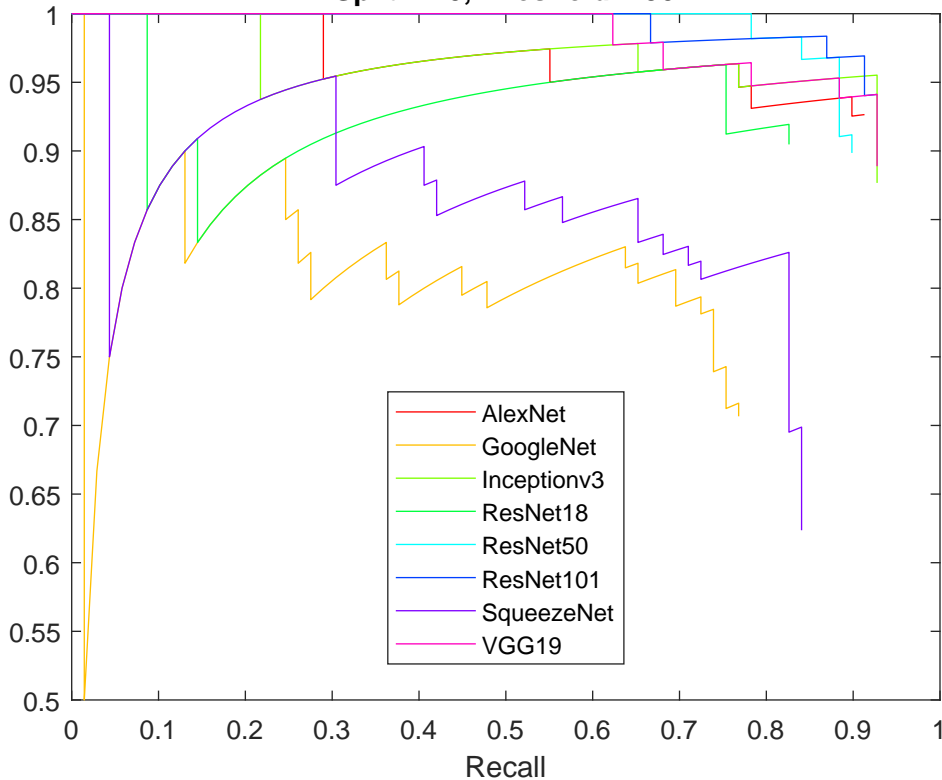

Recall

Supplement: S5 Fig — (PDF) [file pone.0312763.s008.pdf]

Split = 70, Theshold = 70

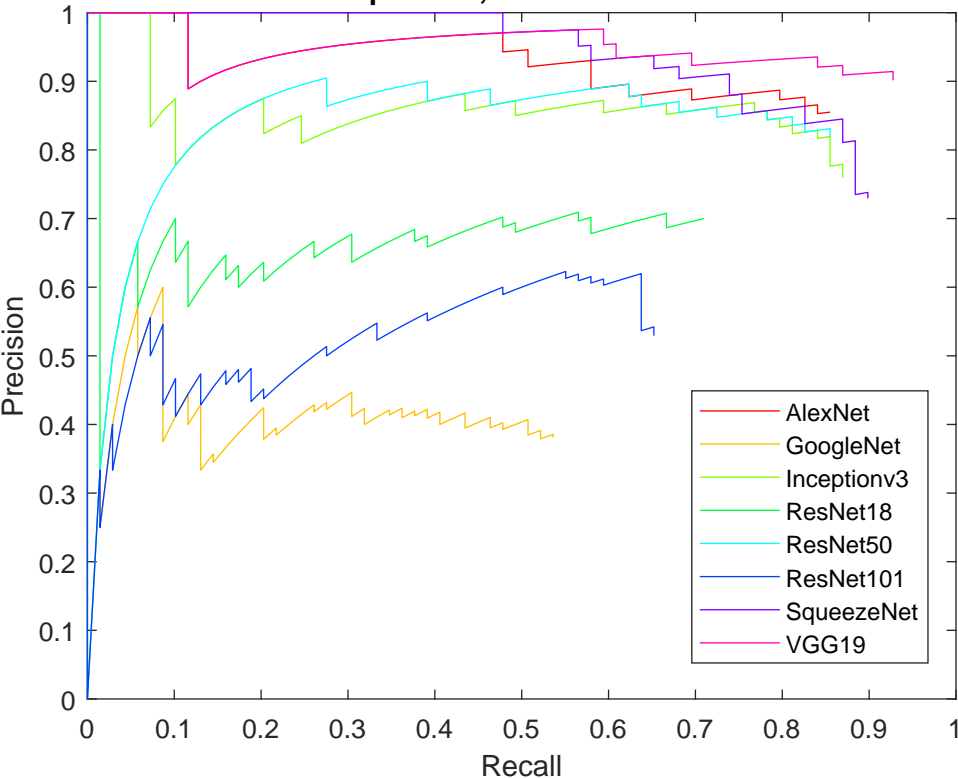

Supplement: S6 Fig — (PDF) [file pone.0312763.s009.pdf]

Split = 80, Theshold = 50

Precision

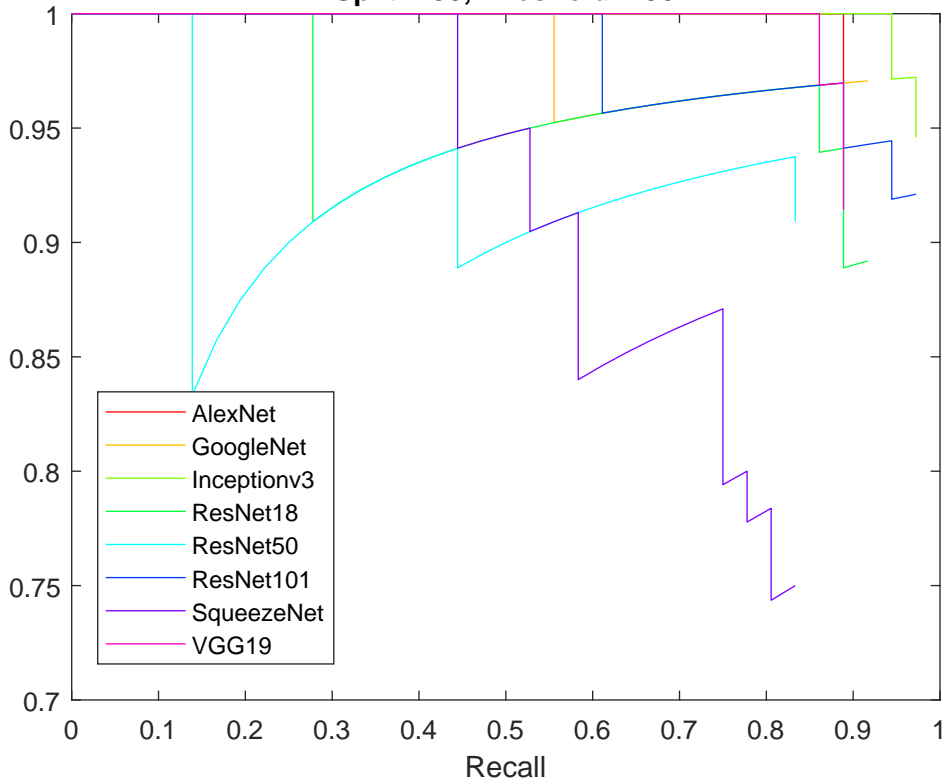

Recall

Supplement: S7 Fig — (PDF) [file pone.0312763.s010.pdf]

Split = 80, Theshold = 70

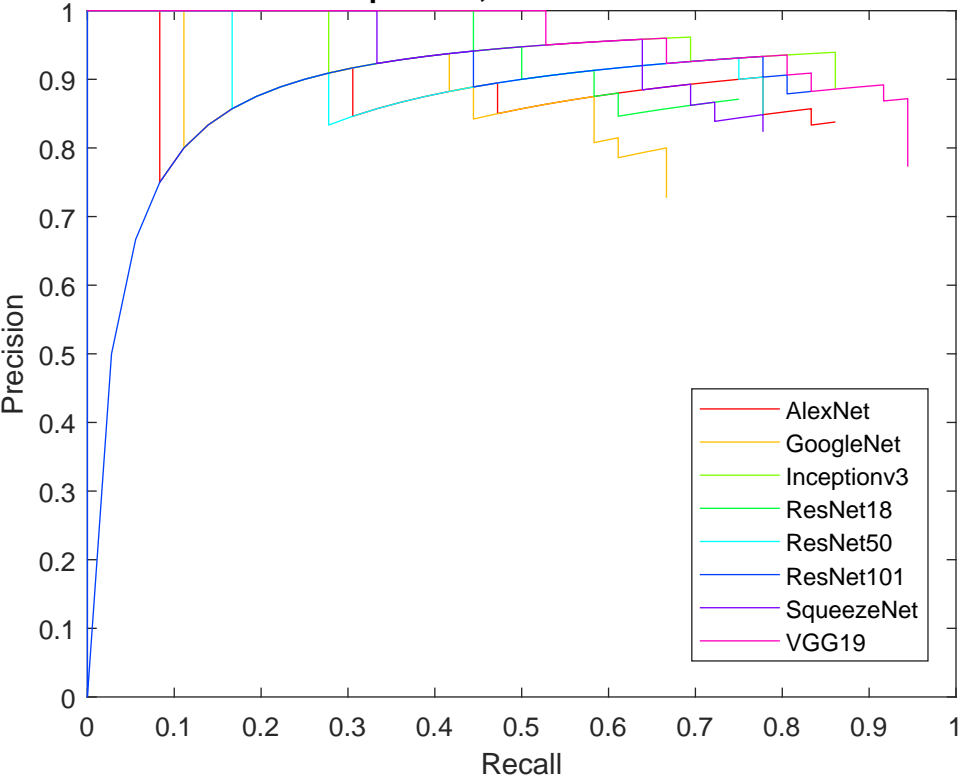

Supplement: S8 Fig — (PDF) [file pone.0312763.s011.pdf]

Split = 50, Theshold = 50

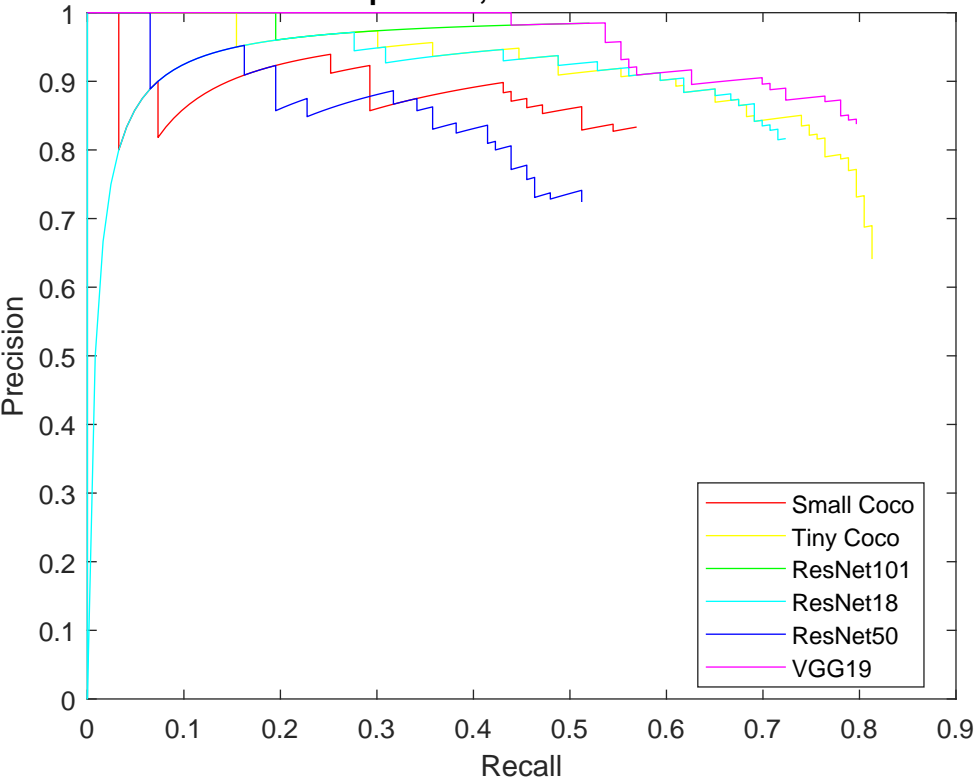

Supplement: S9 Fig — (PDF) [file pone.0312763.s012.pdf]

**Split = 50, Theshold = 70**

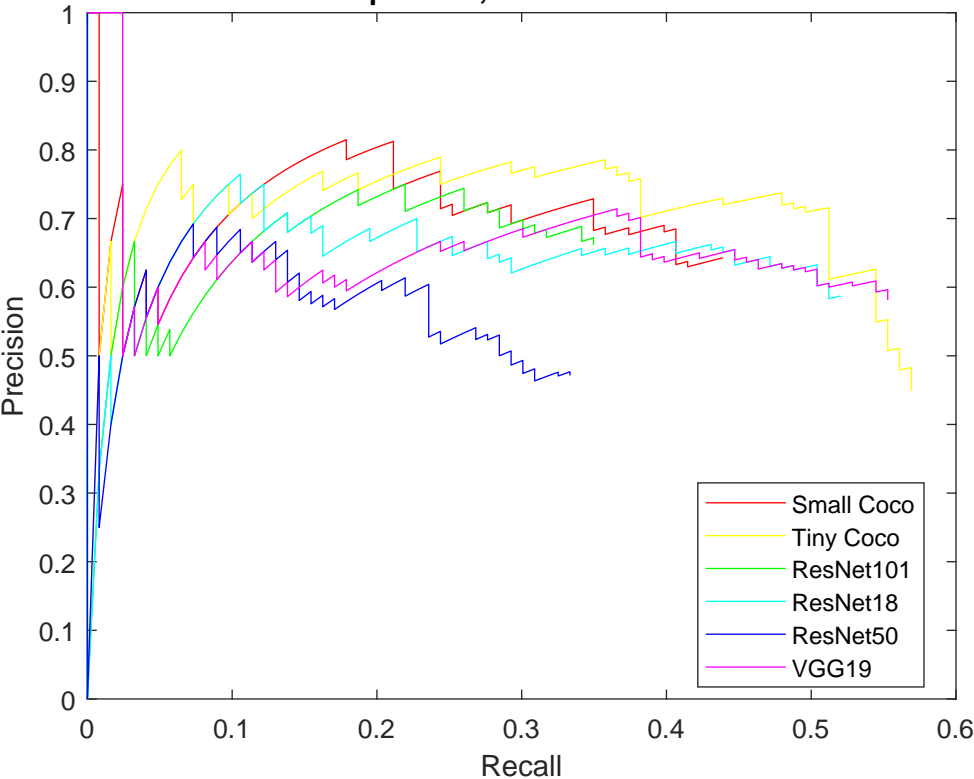

Supplement: S10 Fig — (PDF) [file pone.0312763.s013.pdf]

Split = 60, Theshold = 50

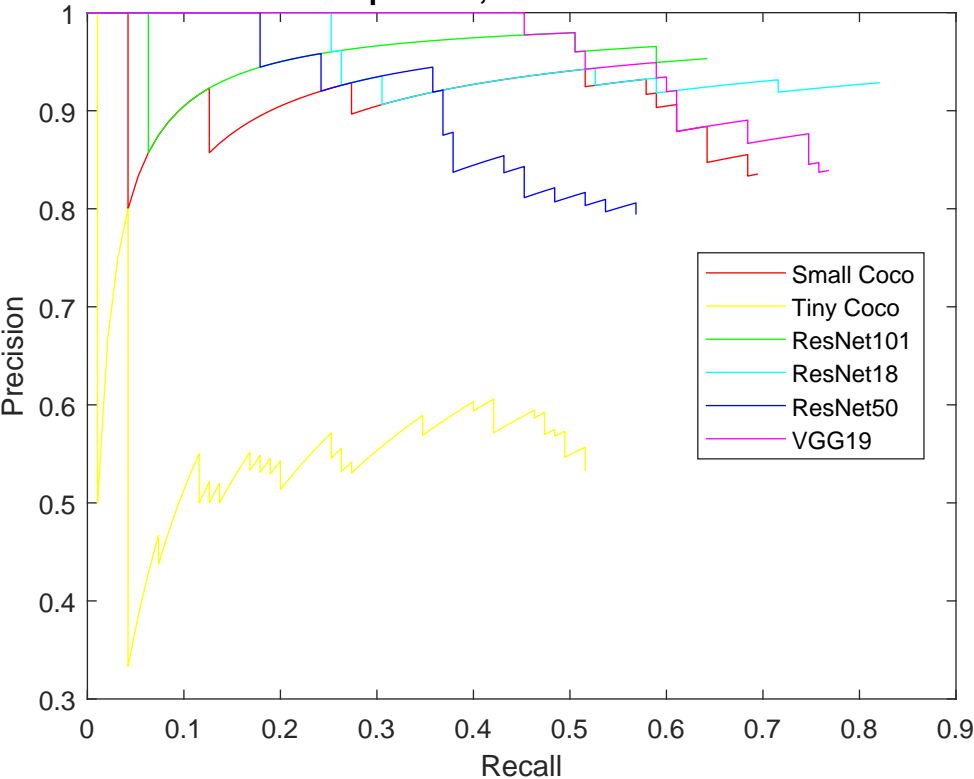

Supplement: S11 Fig — (PDF) [file pone.0312763.s014.pdf]

Split = 60, Theshold = 70

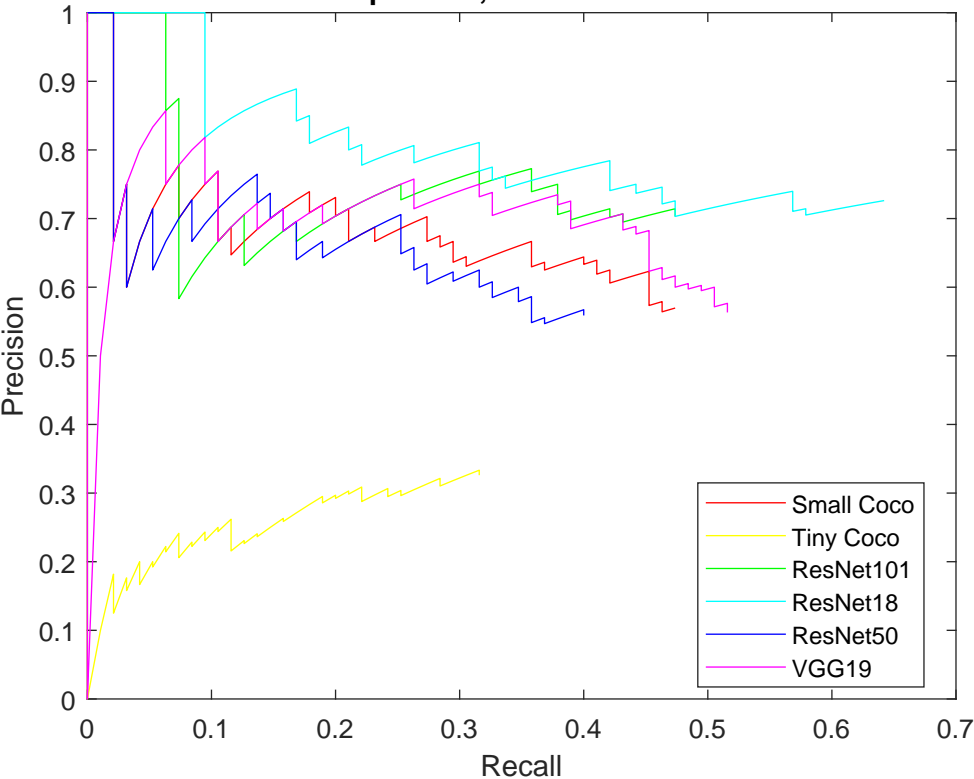

Supplement: S12 Fig — (PDF) [file pone.0312763.s015.pdf]

**Split = 70, Theshold = 50**

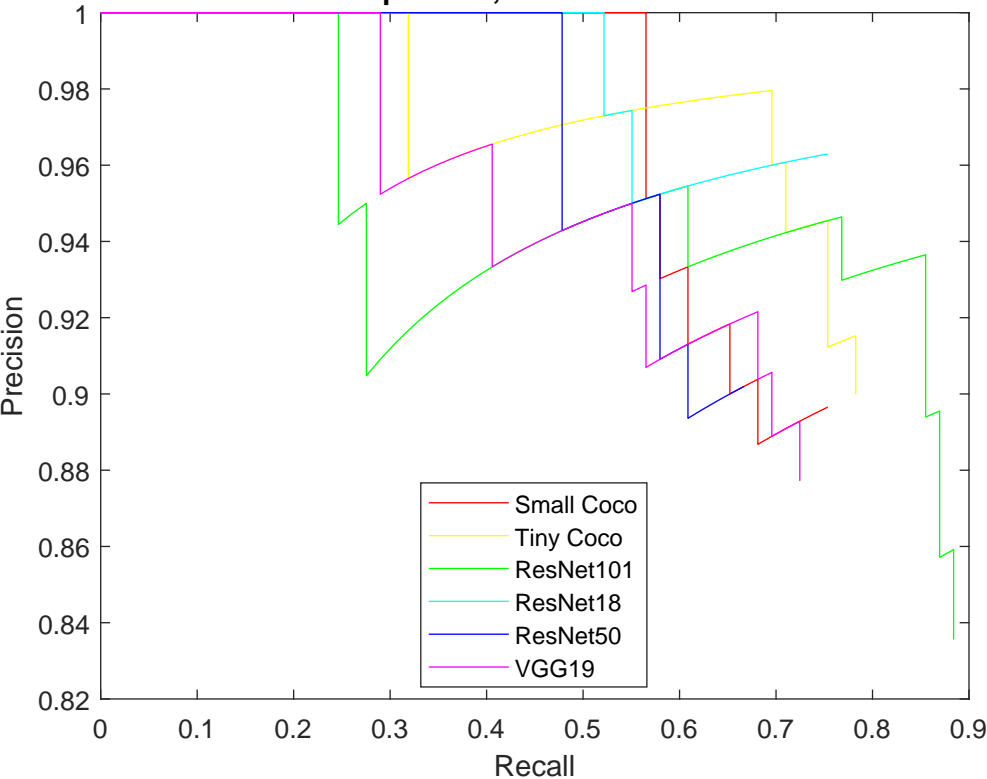

Supplement: S13 Fig — (PDF) [file pone.0312763.s016.pdf]

Split = 70, Theshold = 70

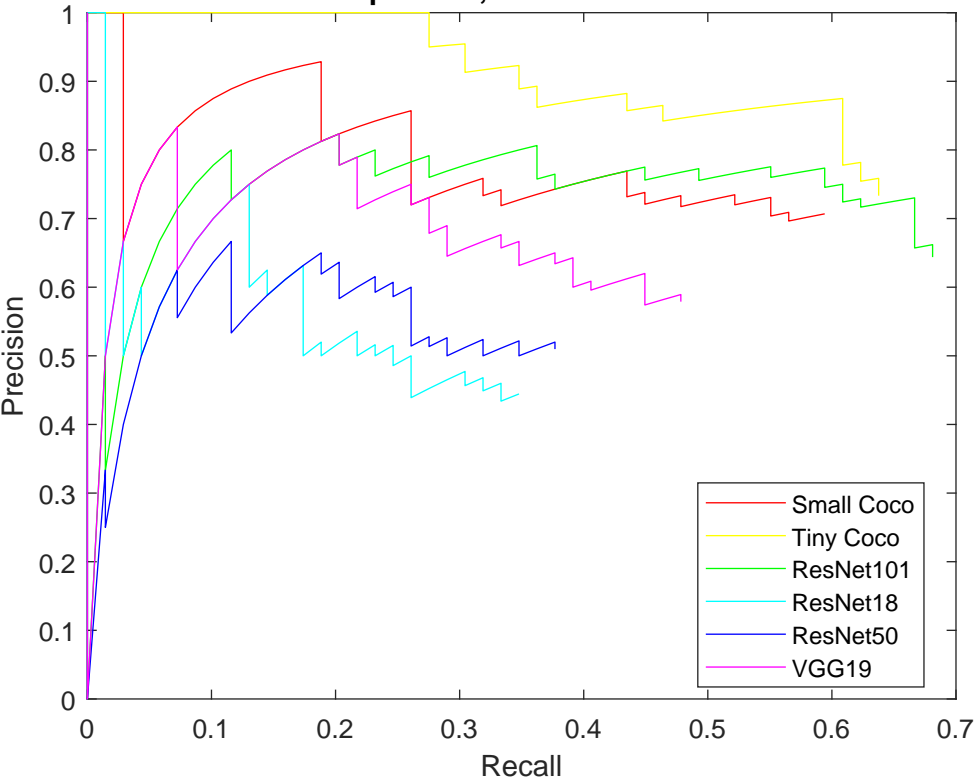

Supplement: S14 Fig — (PDF) [file pone.0312763.s017.pdf]

**Split = 80, Theshold = 50**

Precision

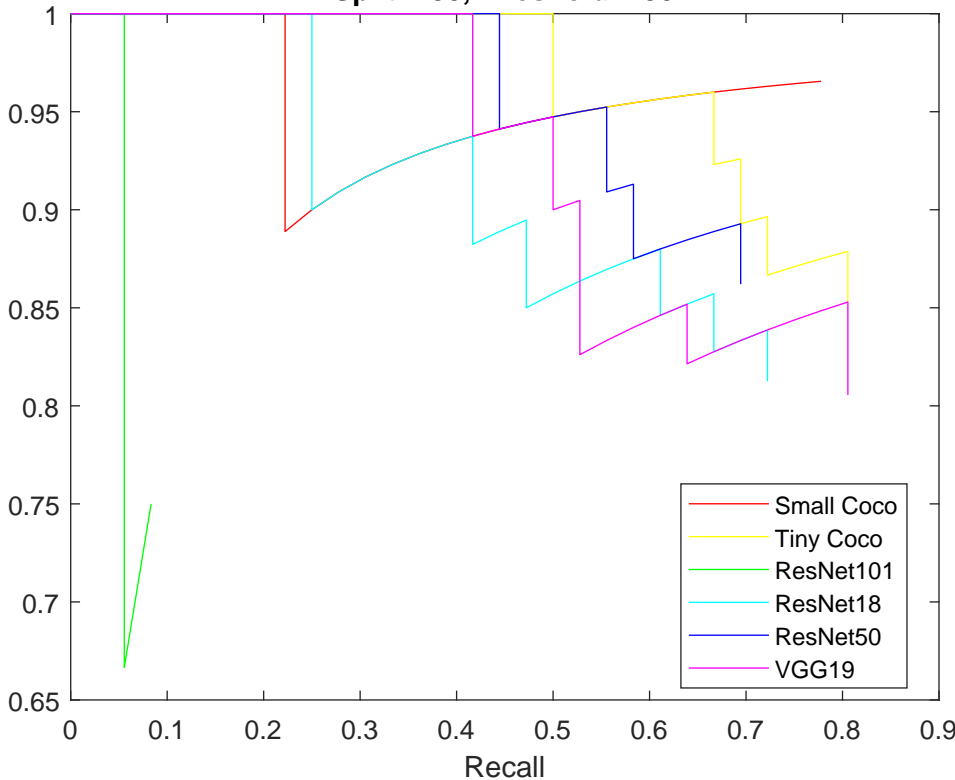

Recall

Supplement: S15 Fig — (PDF) [file pone.0312763.s018.pdf]

Split = 80, Theshold = 70

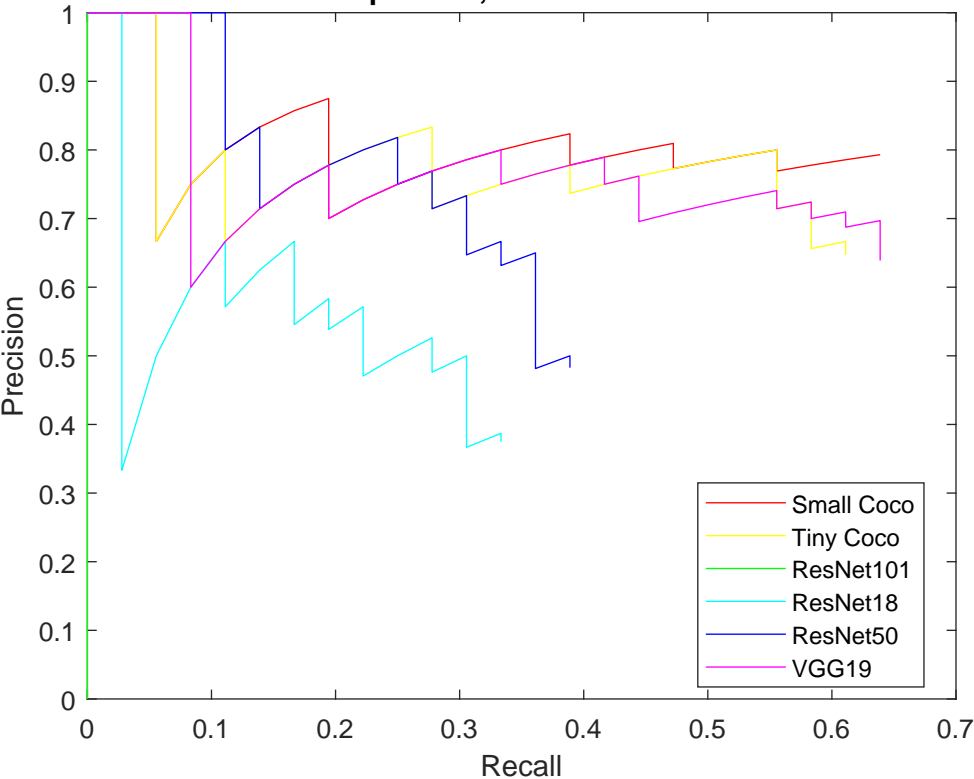

Supplement: S16 Fig — (PDF) [file pone.0312763.s019.pdf]

**Split = 50, Theshold = 50**

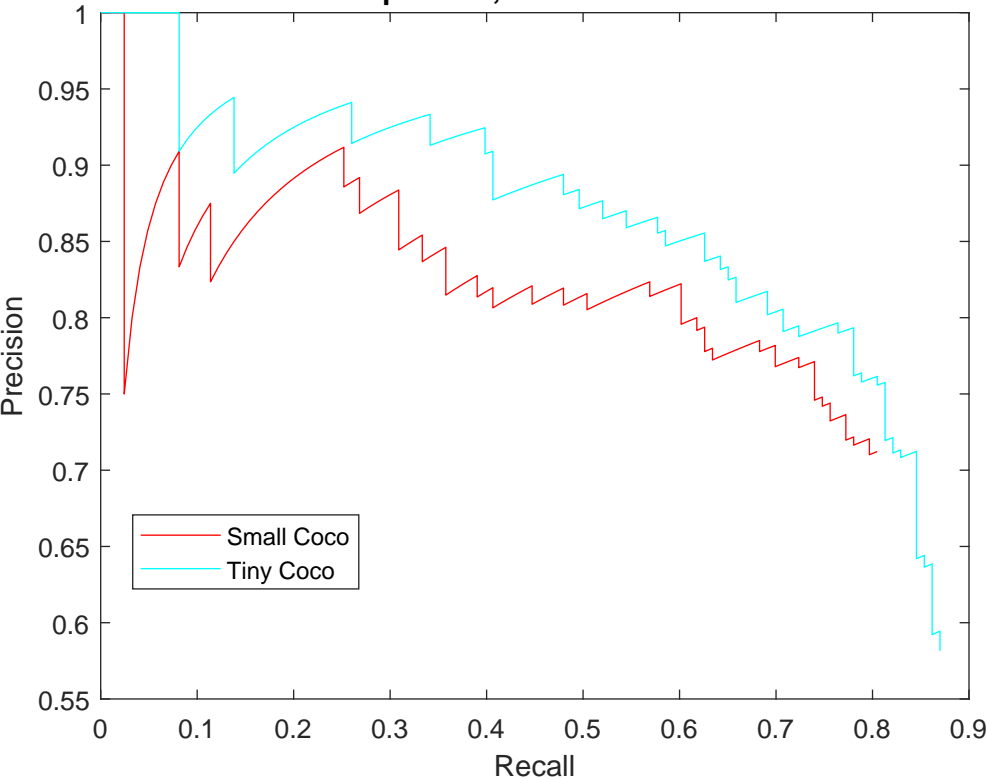

Supplement: S17 Fig — (PDF) [file pone.0312763.s020.pdf]

Split = 50, Theshold = 70

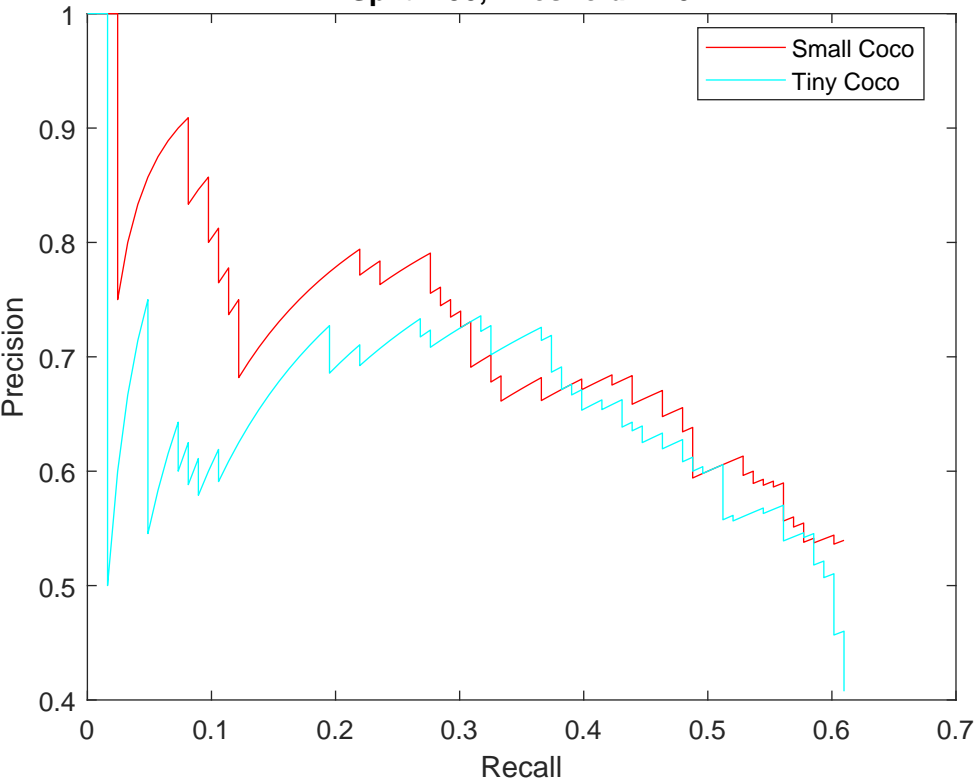

Supplement: S18 Fig — (PDF) [file pone.0312763.s021.pdf]

**Split = 60, Theshold = 50**

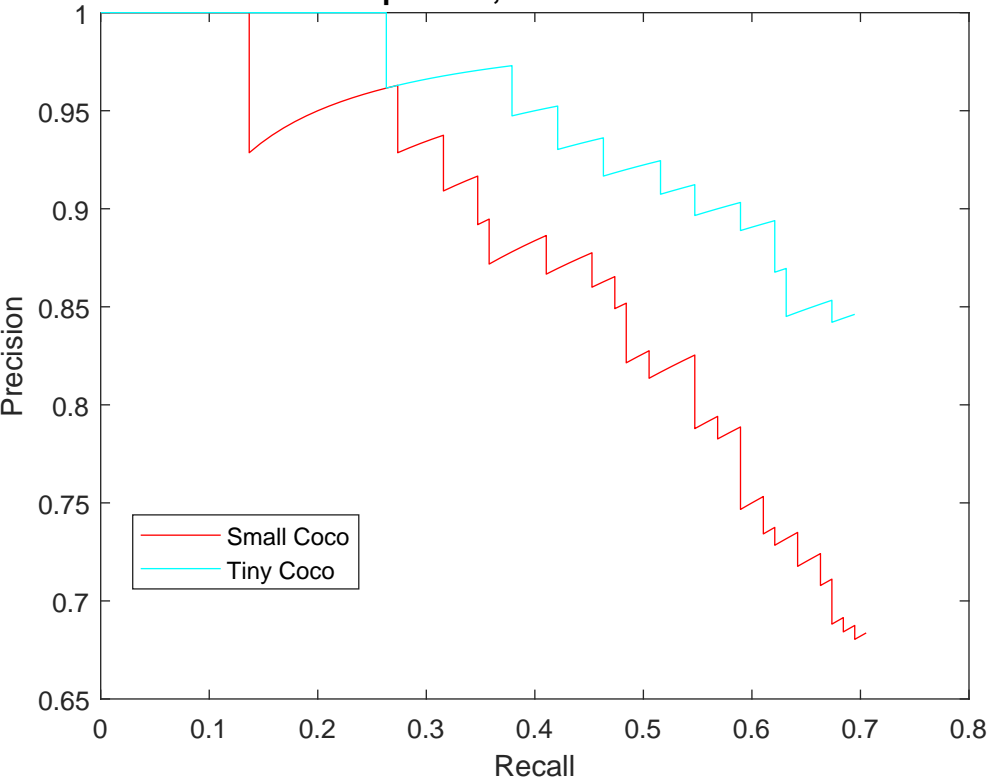

Supplement: S19 Fig — (PDF) [file pone.0312763.s022.pdf]

**Split = 60, Theshold = 70**

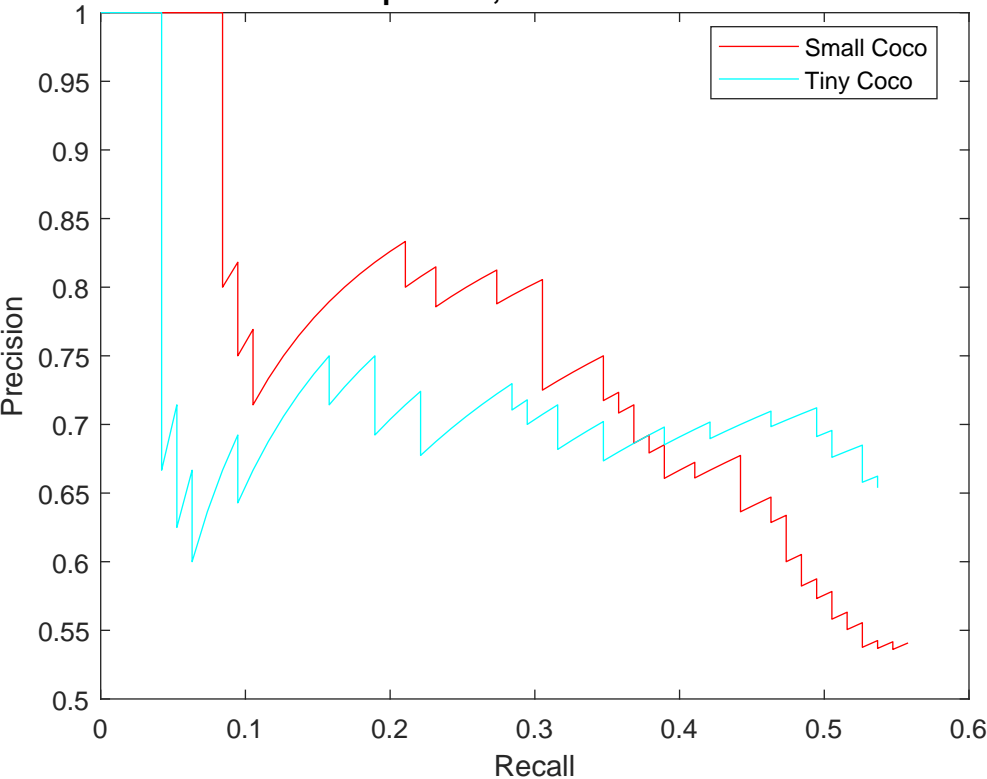

Supplement: S20 Fig — (PDF) [file pone.0312763.s023.pdf]

**Split = 70, Theshold = 50**

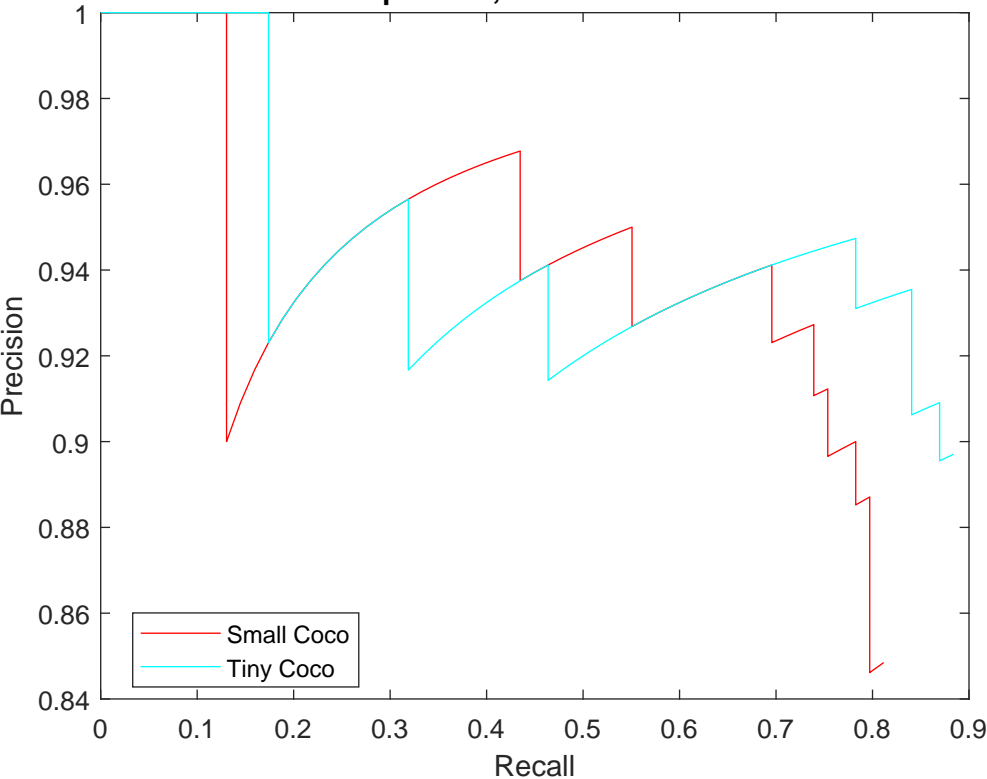

Supplement: S21 Fig — (PDF) [file pone.0312763.s024.pdf]

**Split = 70, Theshold = 70**

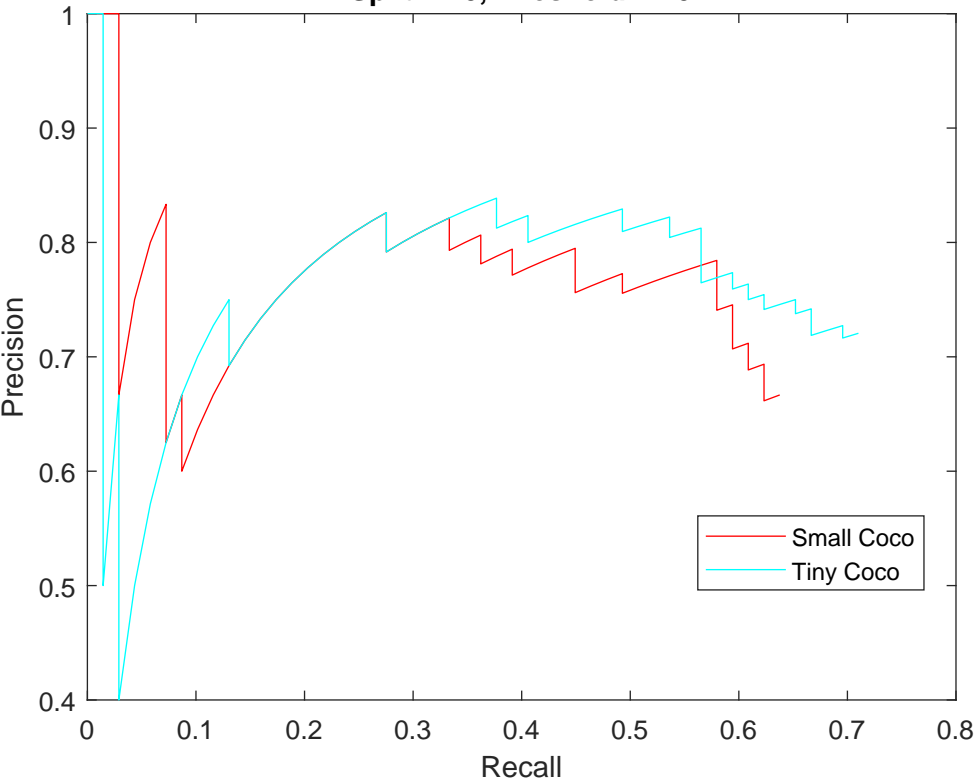

Supplement: S22 Fig — (PDF) [file pone.0312763.s025.pdf]

Split = 80, Theshold = 50

Precision

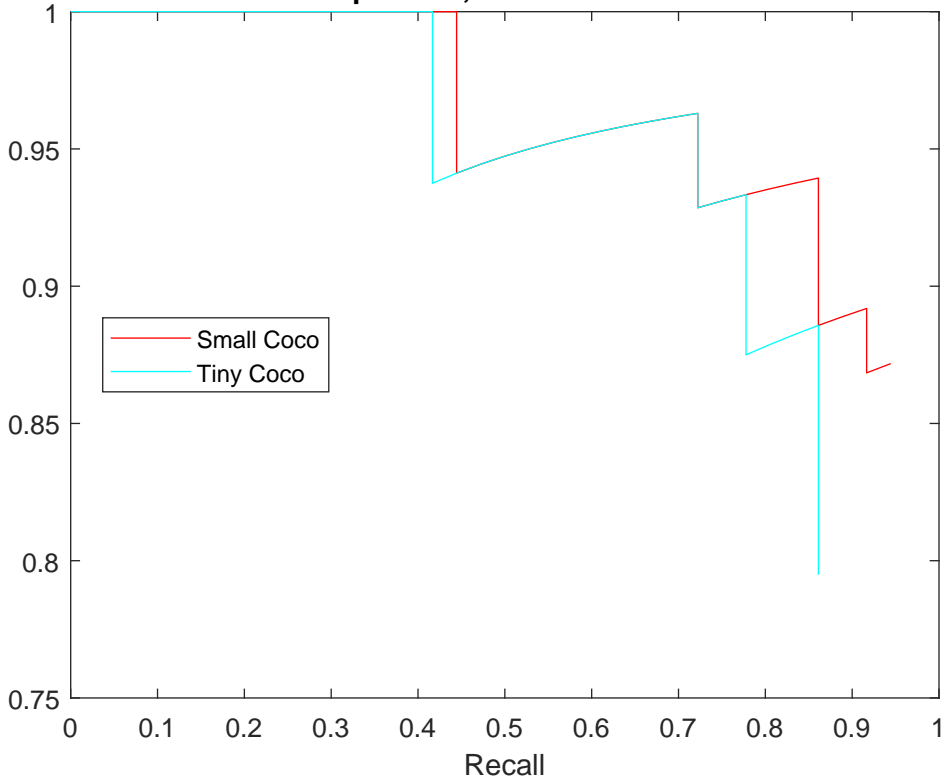

Supplement: S23 Fig — (PDF) [file pone.0312763.s026.pdf]

Split = 80, Theshold = 70

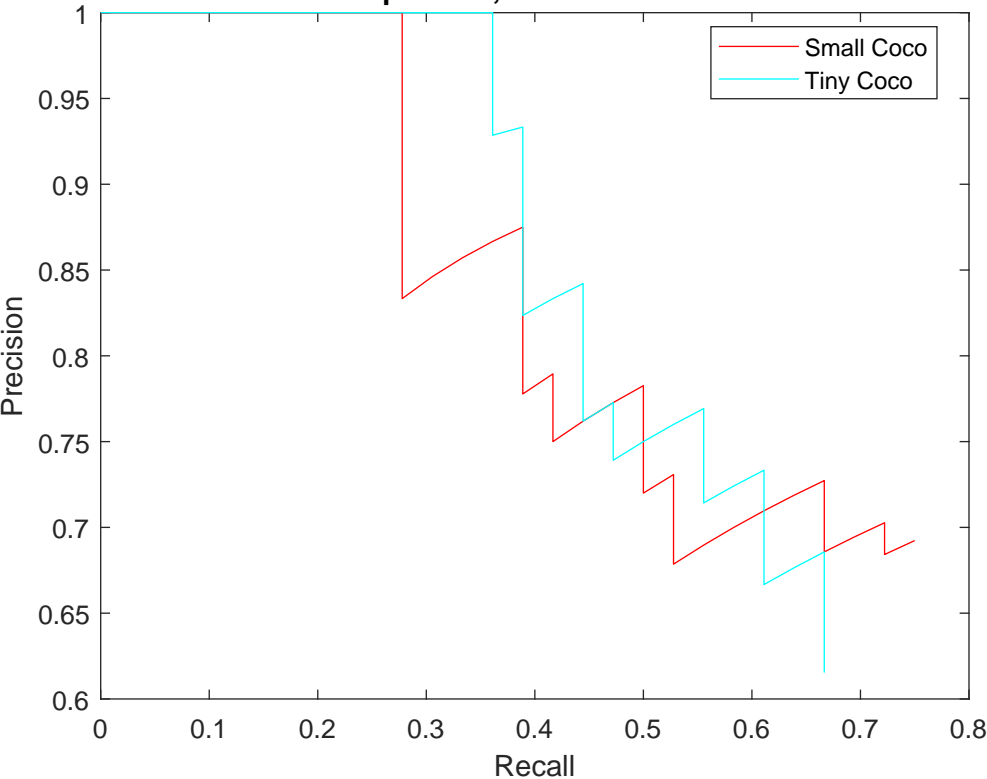

Supplement: S24 Fig — (PDF) [file pone.0312763.s027.pdf]

Split = 50, Theshold = 50

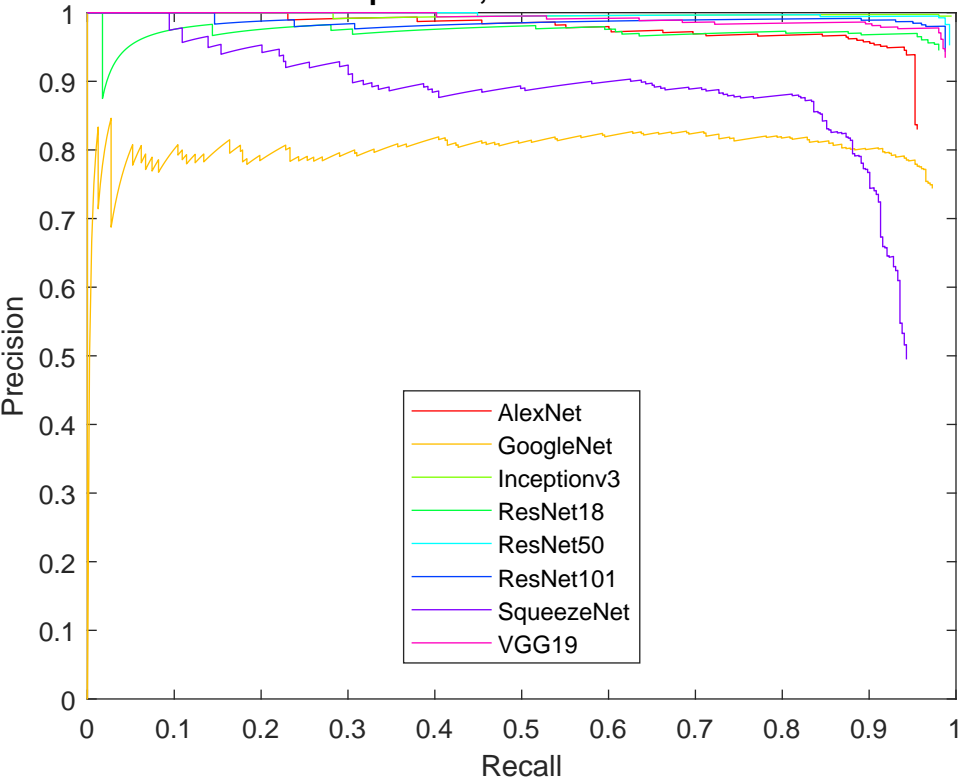

Supplement: S25 Fig — (PDF) [file pone.0312763.s028.pdf]

Split = 50, Theshold = 70

Precision

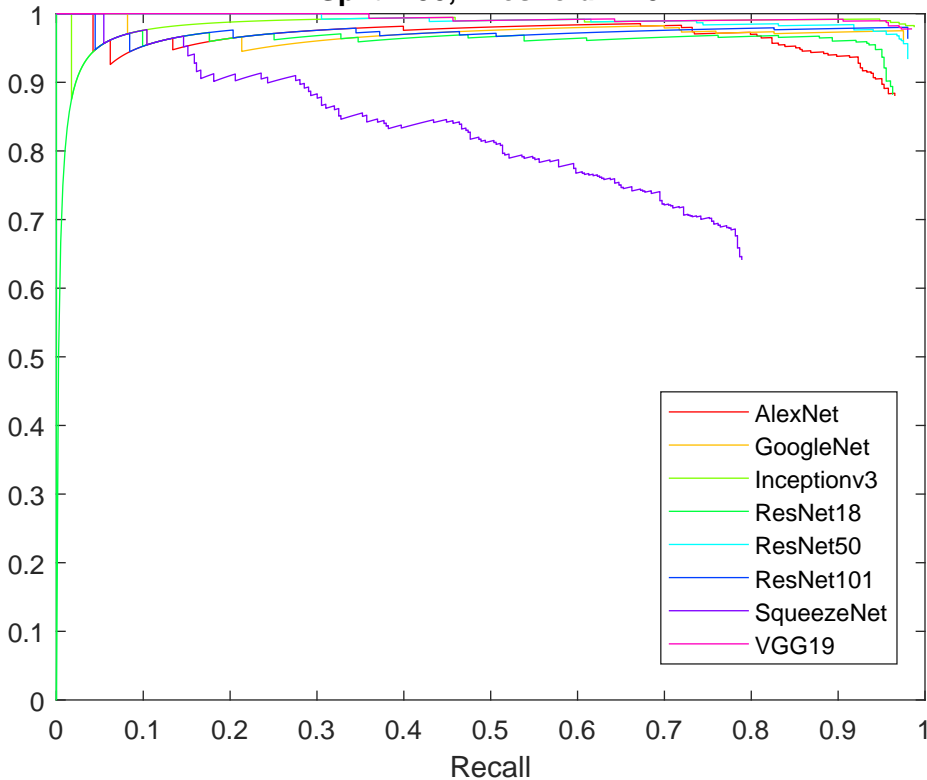

Recall

Supplement: S26 Fig — (PDF) [file pone.0312763.s029.pdf]

Split = 60, Theshold = 50

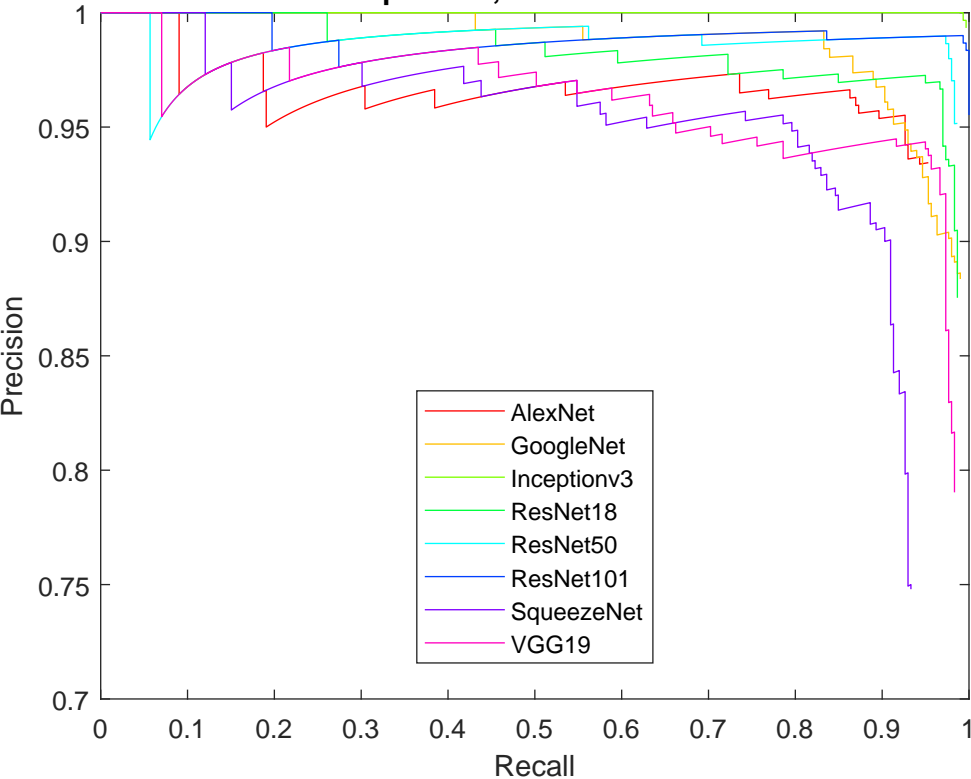

Supplement: S27 Fig — (PDF) [file pone.0312763.s030.pdf]

Split = 60, Theshold = 70

Precision

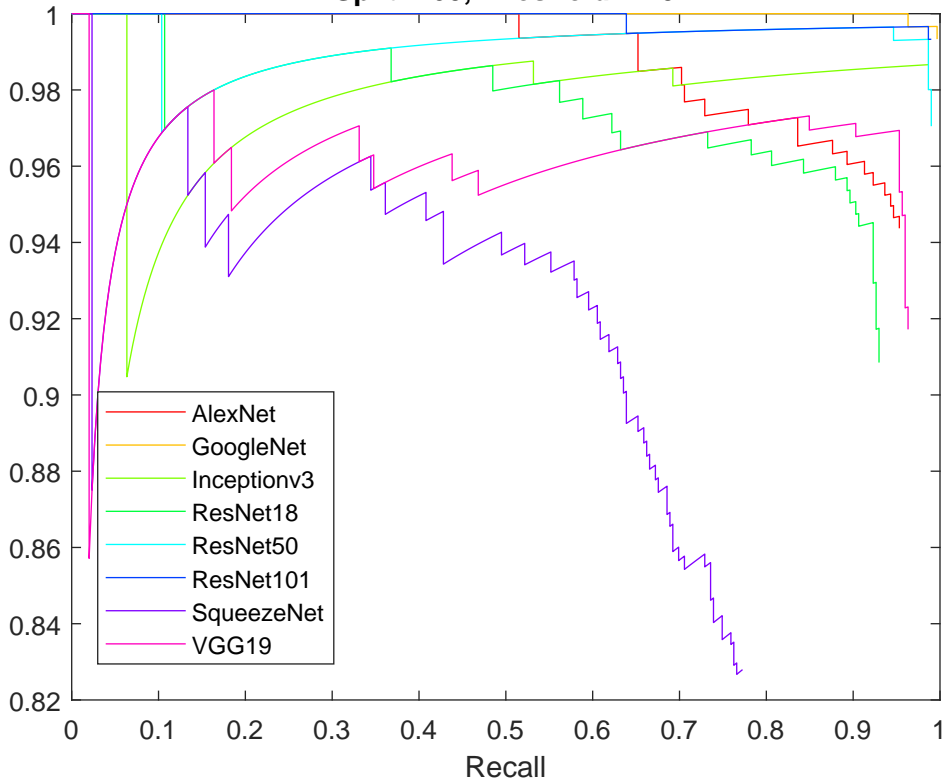

Recall

Supplement: S28 Fig — (PDF) [file pone.0312763.s031.pdf]

Split = 70, Theshold = 50

Precision

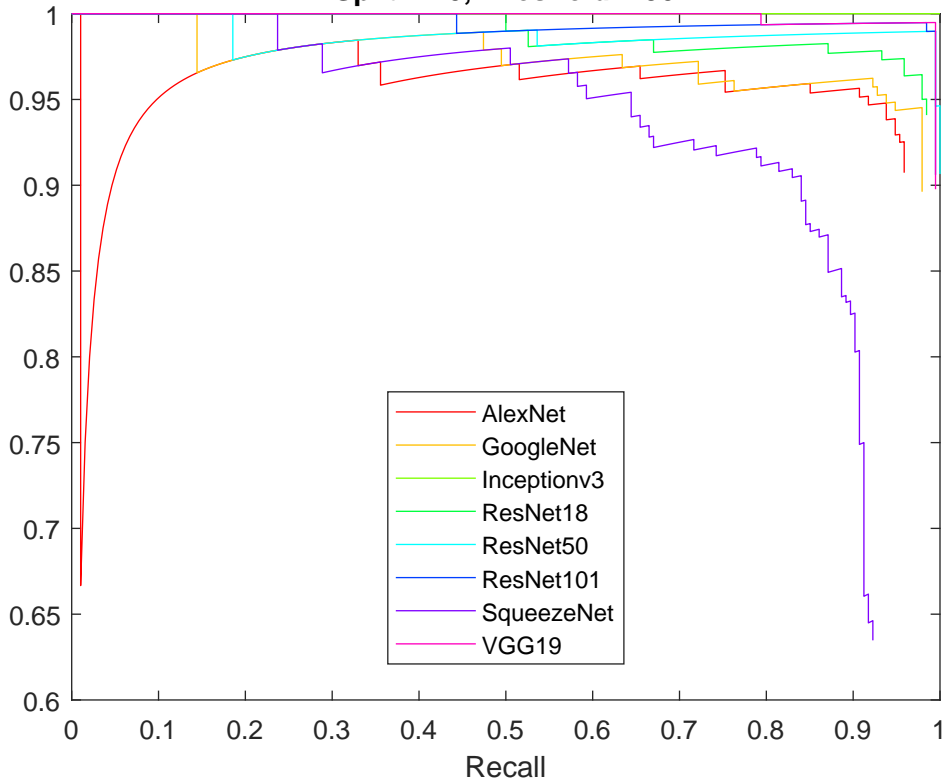

Recall

Supplement: S29 Fig — (PDF) [file pone.0312763.s032.pdf]

Split = 70, Theshold = 70

Precision

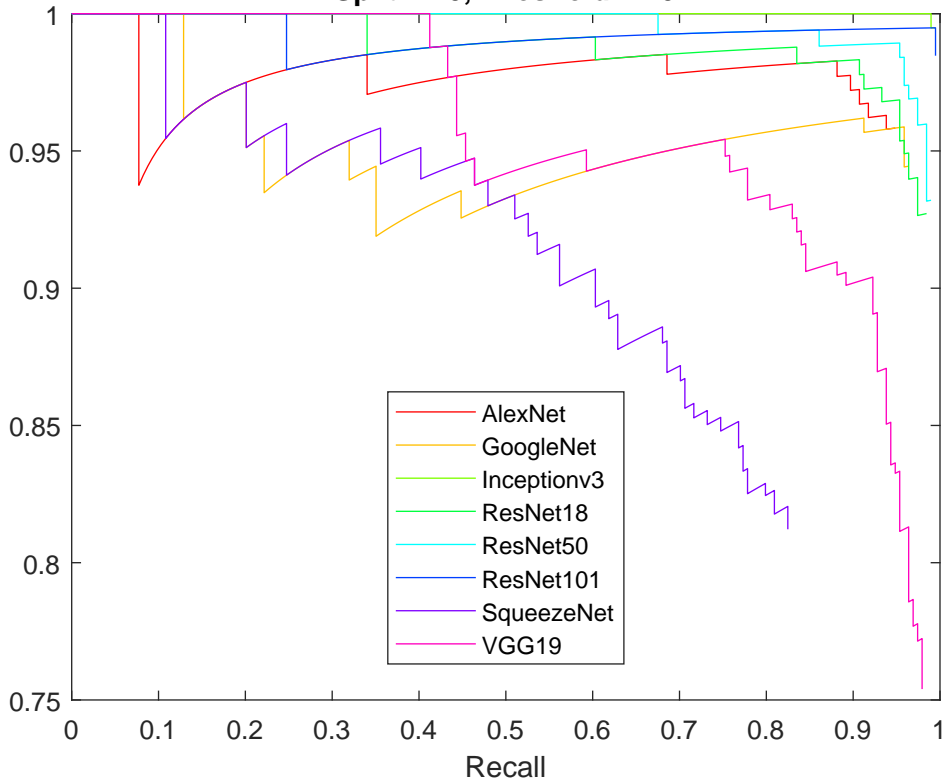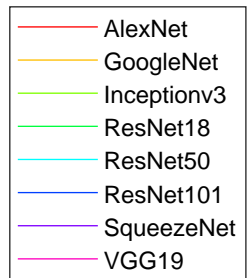

Supplement: S30 Fig — (PDF) [file pone.0312763.s033.pdf]

Split = 80, Theshold = 50

Precision

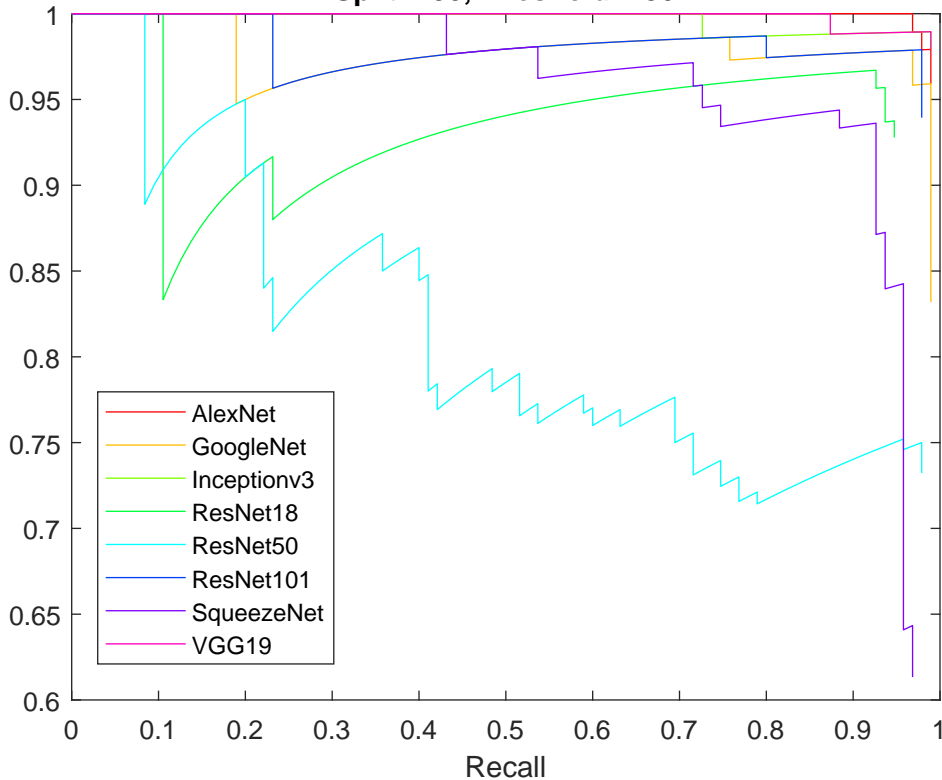

Recall

Supplement: S31 Fig — (PDF) [file pone.0312763.s034.pdf]

Split = 80, Theshold = 70

Precision

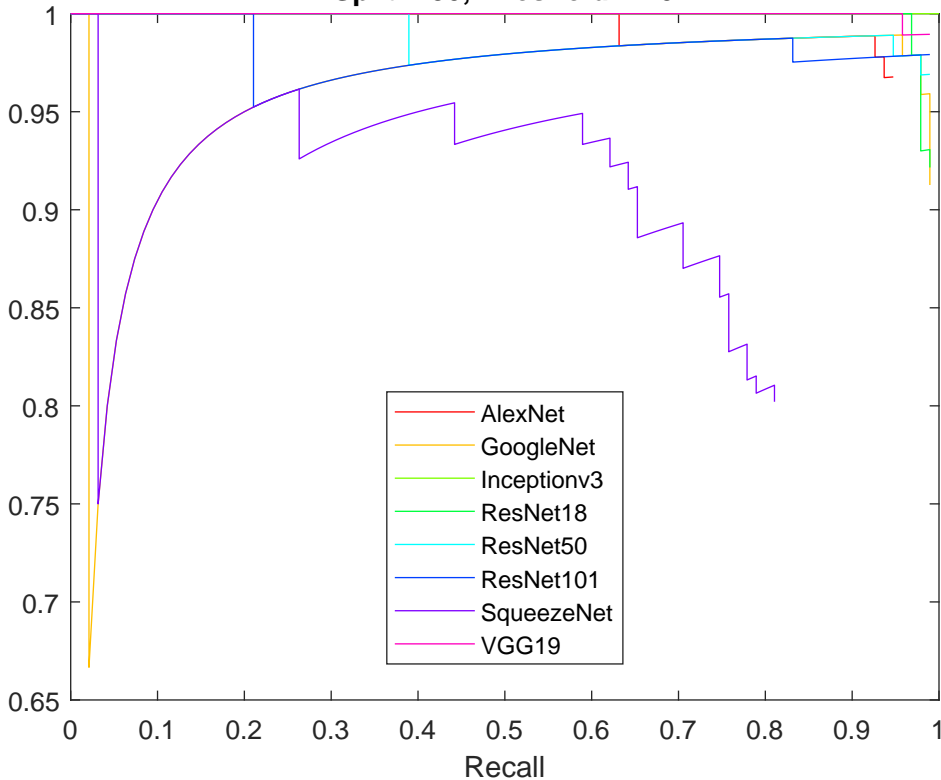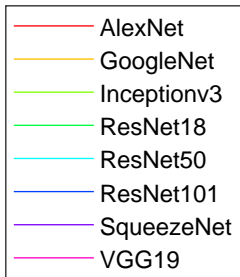

Supplement: S32 Fig — (PDF) [file pone.0312763.s035.pdf]

Split = 50, Theshold = 50

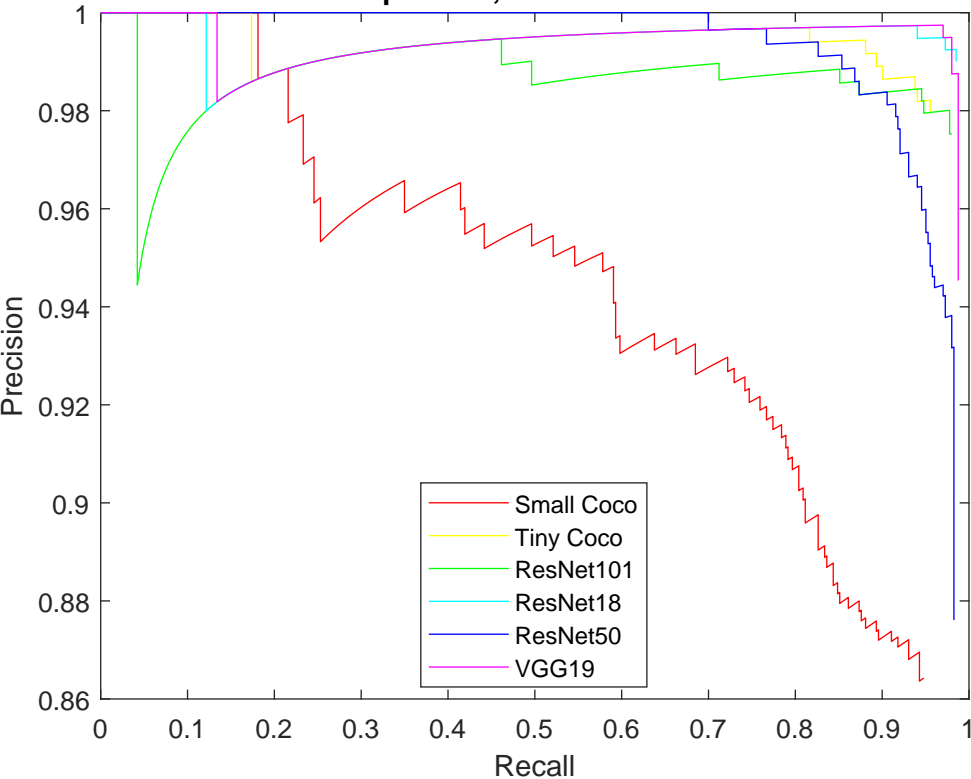

Supplement: S33 Fig — (PDF) [file pone.0312763.s036.pdf]

Split = 50, Theshold = 70

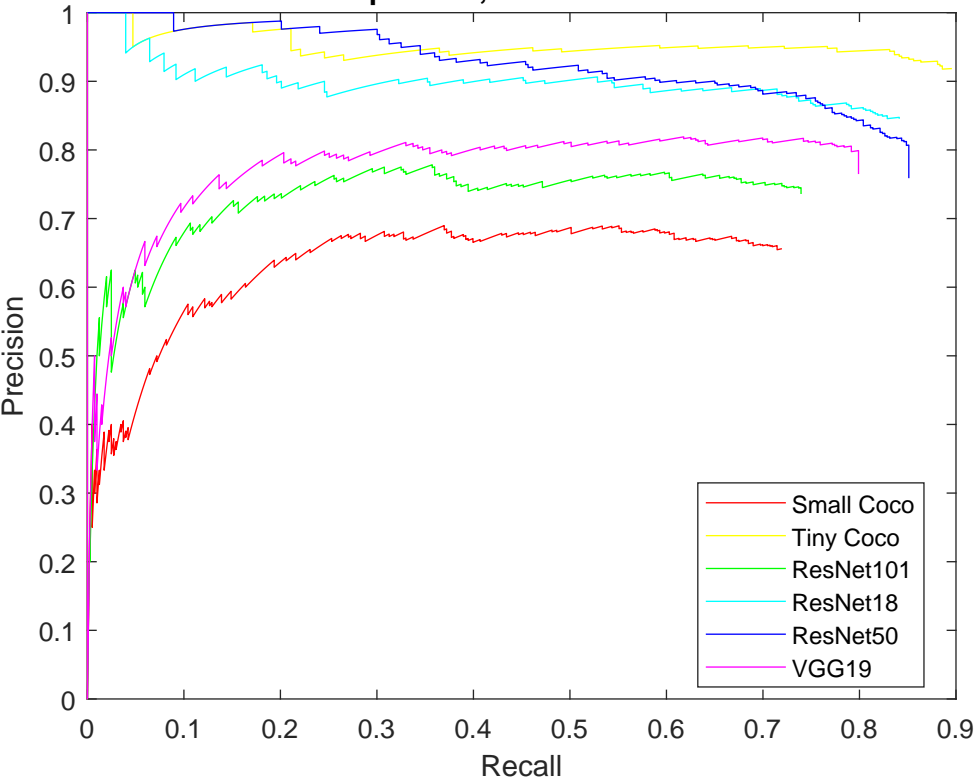

Supplement: S34 Fig — (PDF) [file pone.0312763.s037.pdf]

Split = 60, Theshold = 50

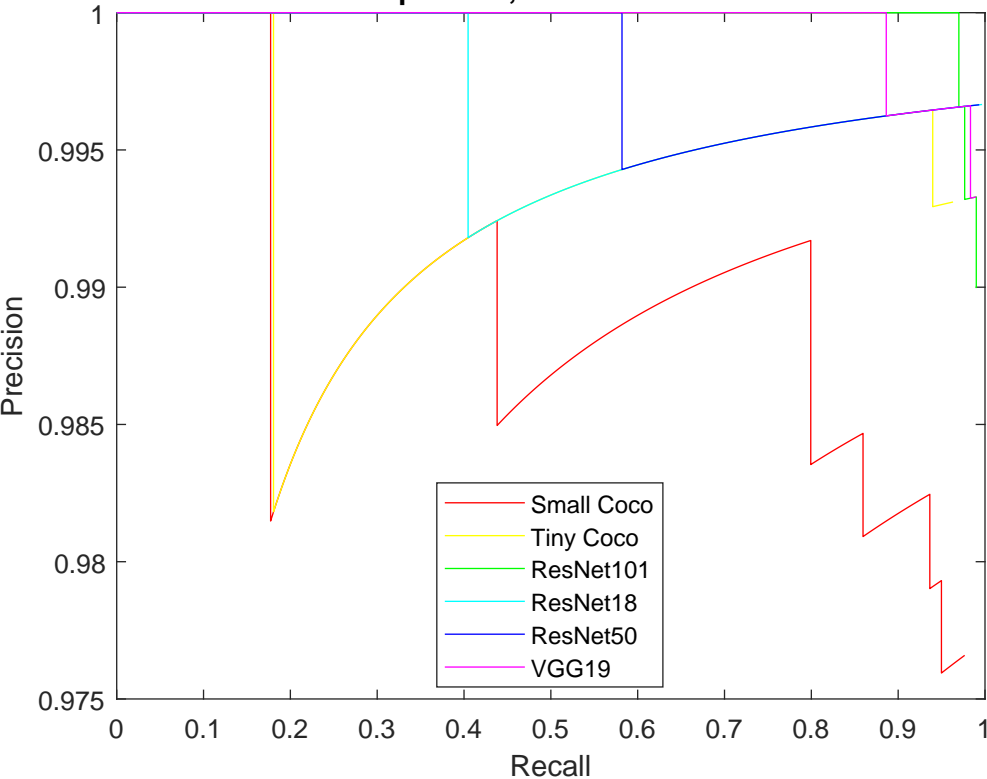

Supplement: S35 Fig — (PDF) [file pone.0312763.s038.pdf]

Split = 60, Theshold = 70

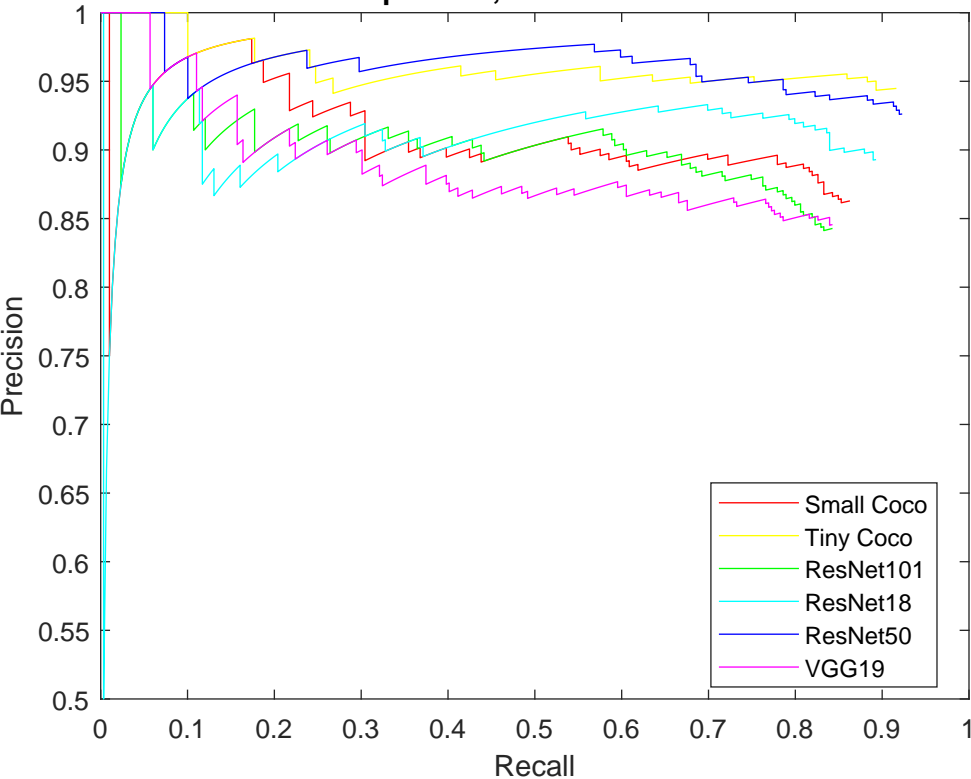

Supplement: S36 Fig — (PDF) [file pone.0312763.s039.pdf]

Split = 70, Theshold = 50

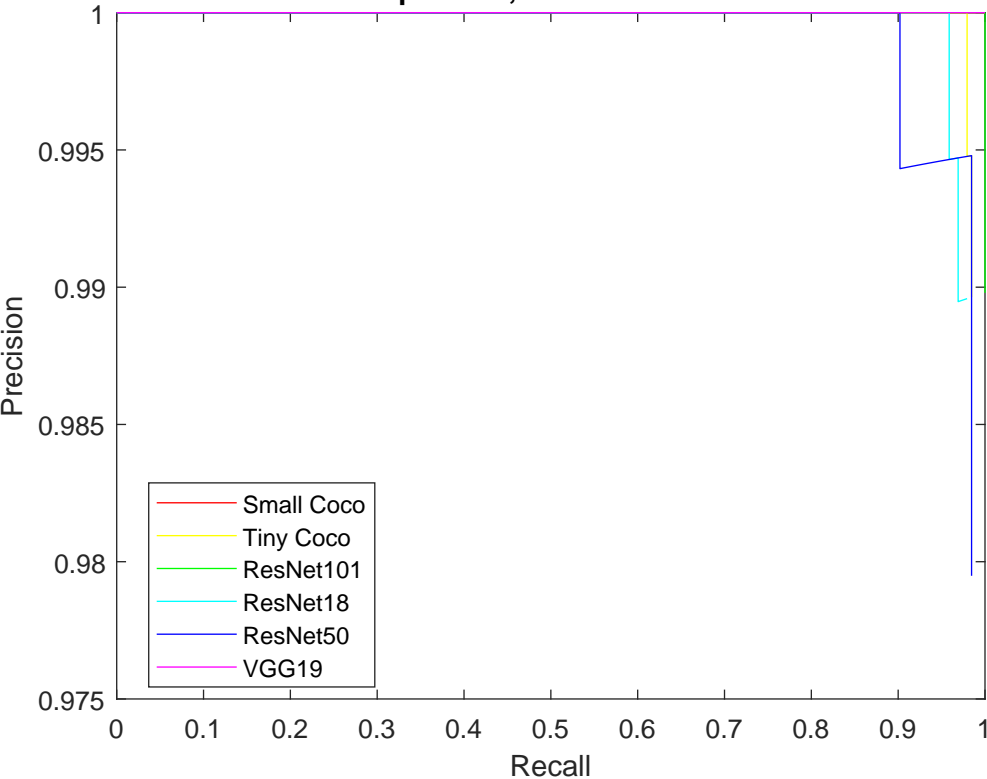

Supplement: S37 Fig — (PDF) [file pone.0312763.s040.pdf]

Split = 70, Theshold = 70

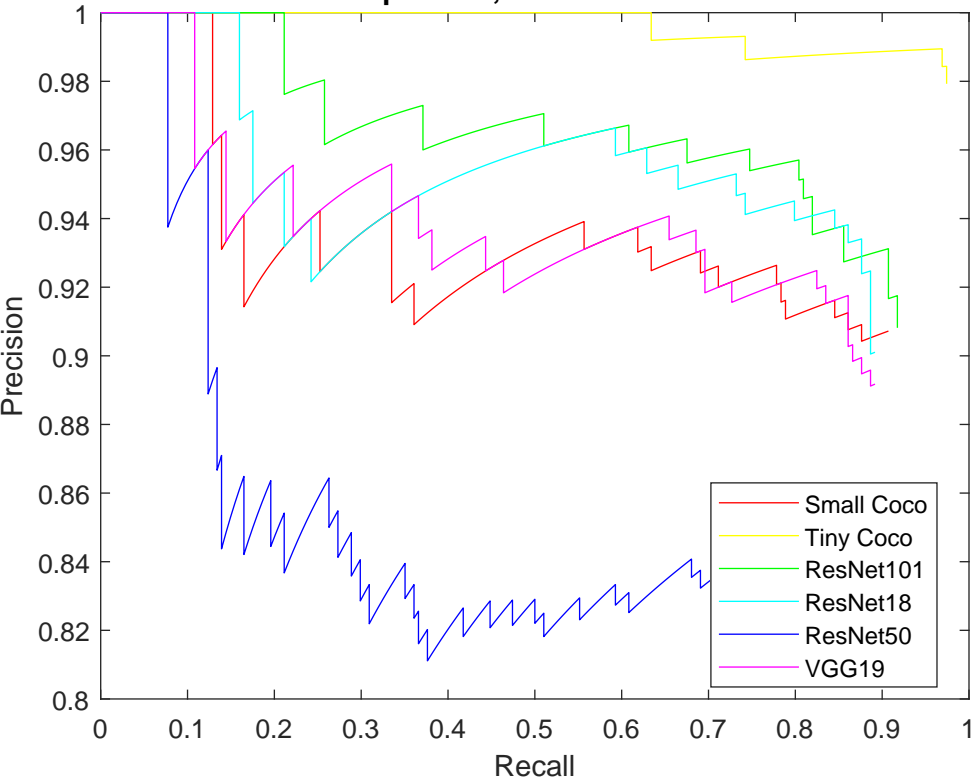

Supplement: S38 Fig — (PDF) [file pone.0312763.s041.pdf]

Split = 80, Theshold = 50

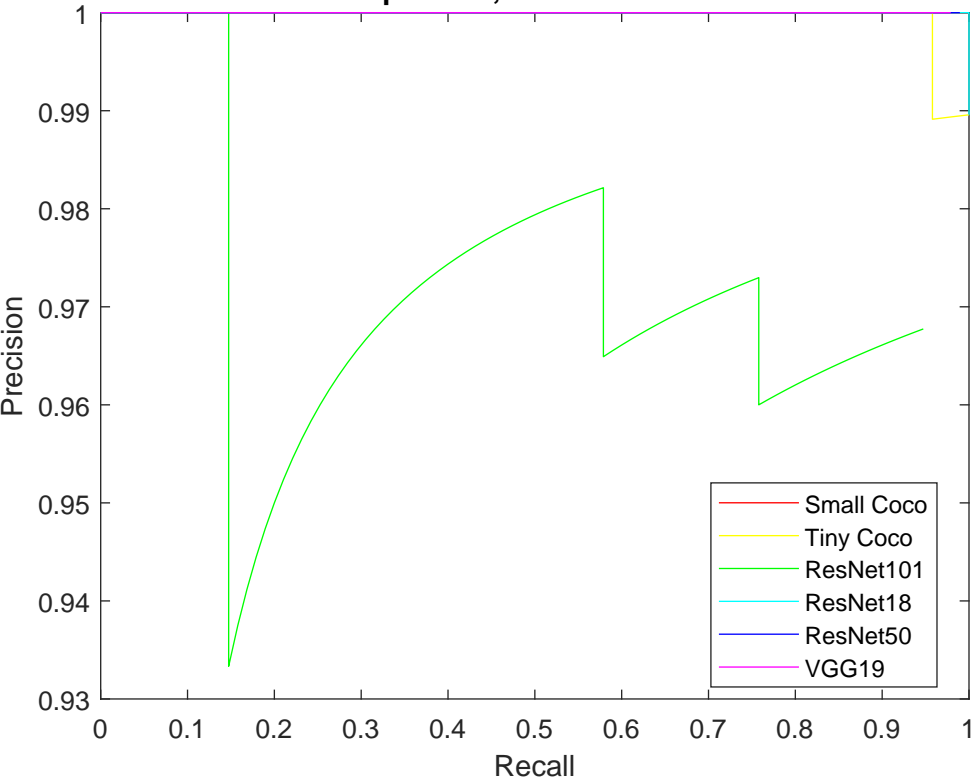

Supplement: S39 Fig — (PDF) [file pone.0312763.s042.pdf]

Split = 80, Theshold = 70

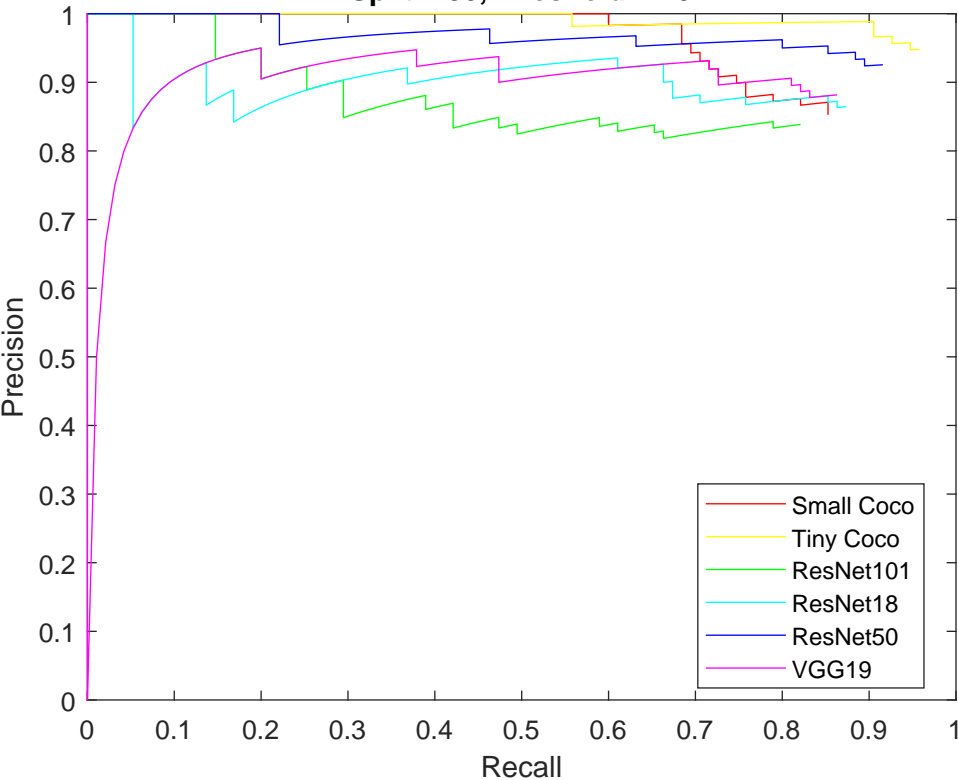

Supplement: S40 Fig — (PDF) [file pone.0312763.s043.pdf]

Split = 50, Theshold = 50

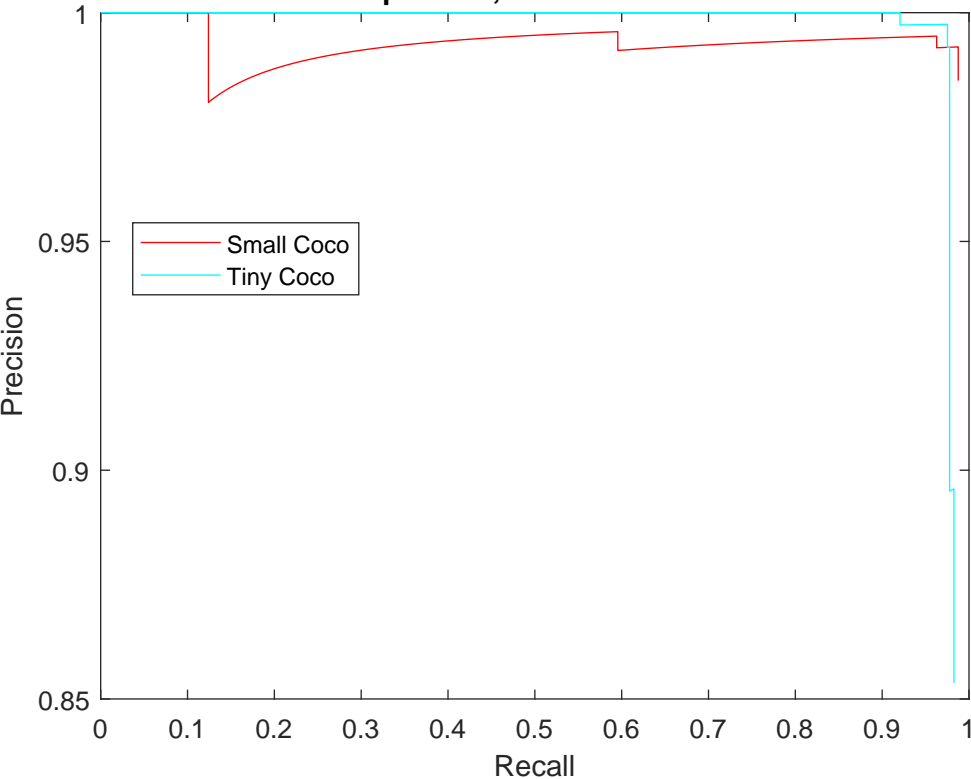

Supplement: S41 Fig — (PDF) [file pone.0312763.s044.pdf]

Split = 50, Theshold = 70

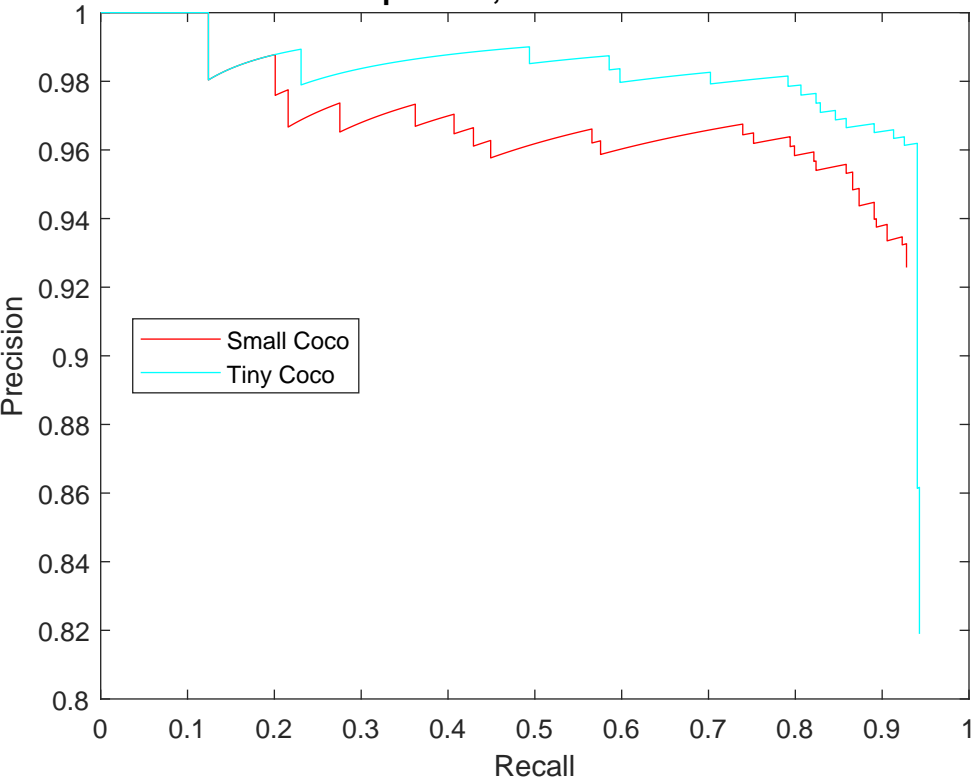

Supplement: S42 Fig — (PDF) [file pone.0312763.s045.pdf]

Split = 60, Theshold = 50

Precision

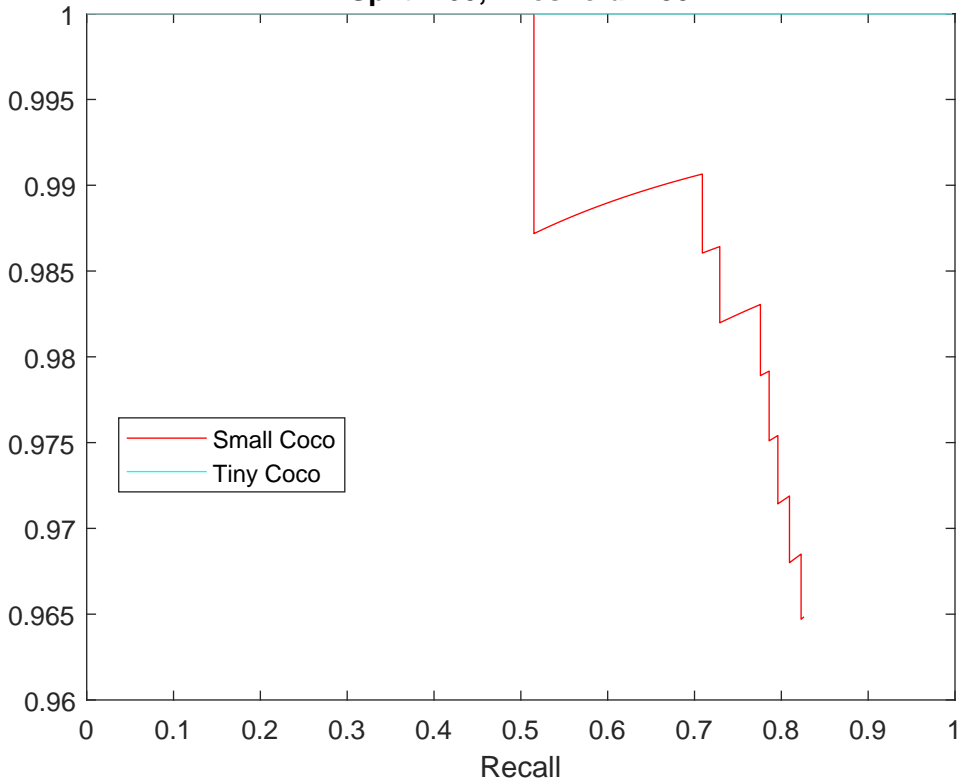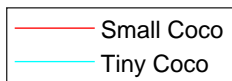

Supplement: S43 Fig — (PDF) [file pone.0312763.s046.pdf]

Split = 60, Theshold = 70

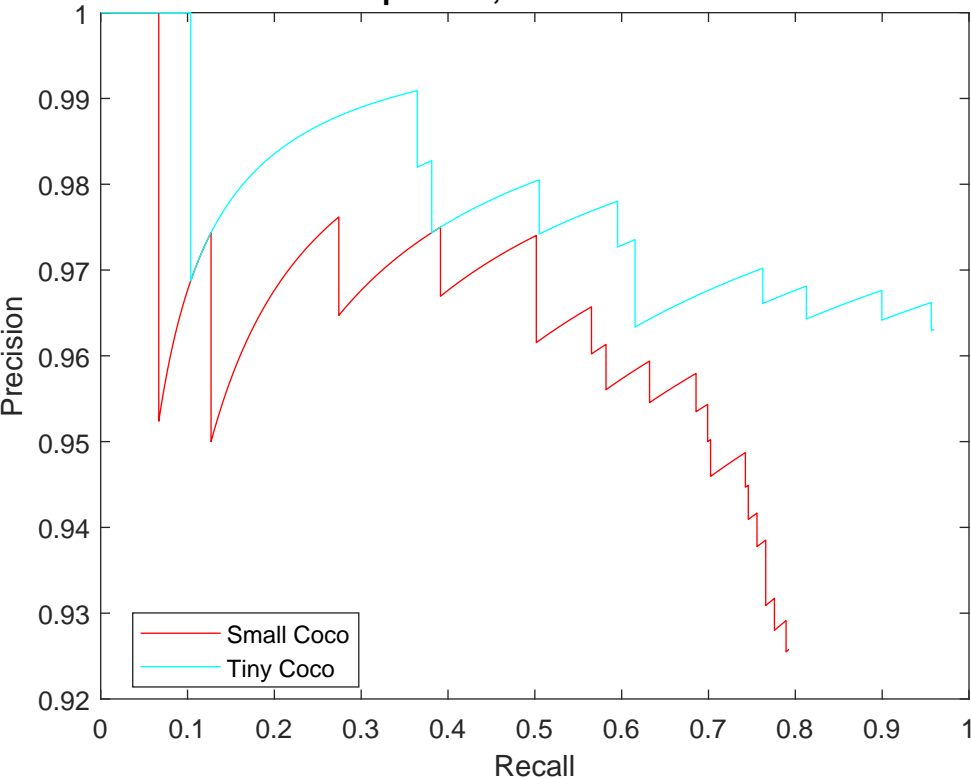

Supplement: S44 Fig — (PDF) [file pone.0312763.s047.pdf]

Split = 70, Theshold = 50

Precision

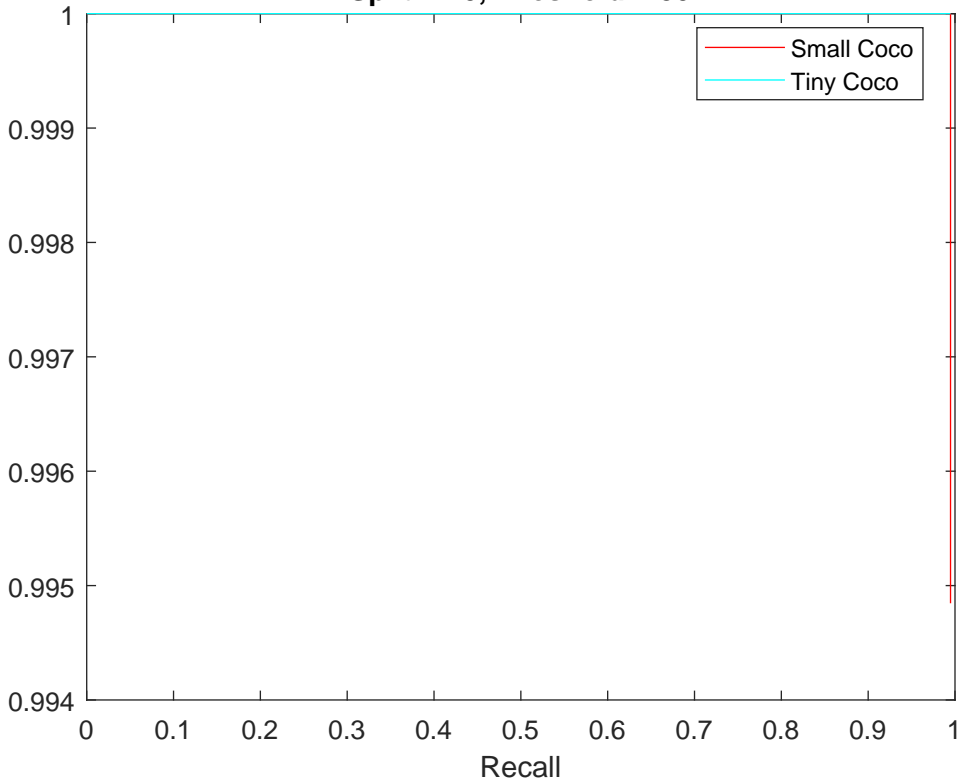

Recall

Supplement: S45 Fig — (PDF) [file pone.0312763.s048.pdf]

Split = 70, Theshold = 70

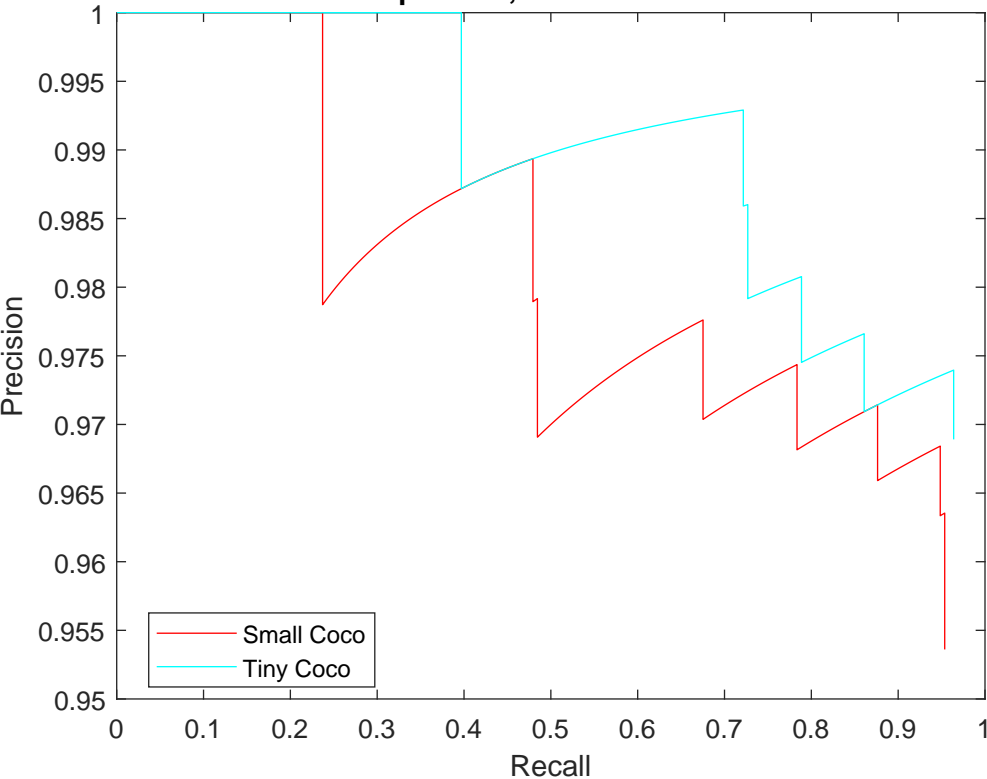

Supplement: S46 Fig — (PDF) [file pone.0312763.s049.pdf]

Split = 80, Theshold = 50

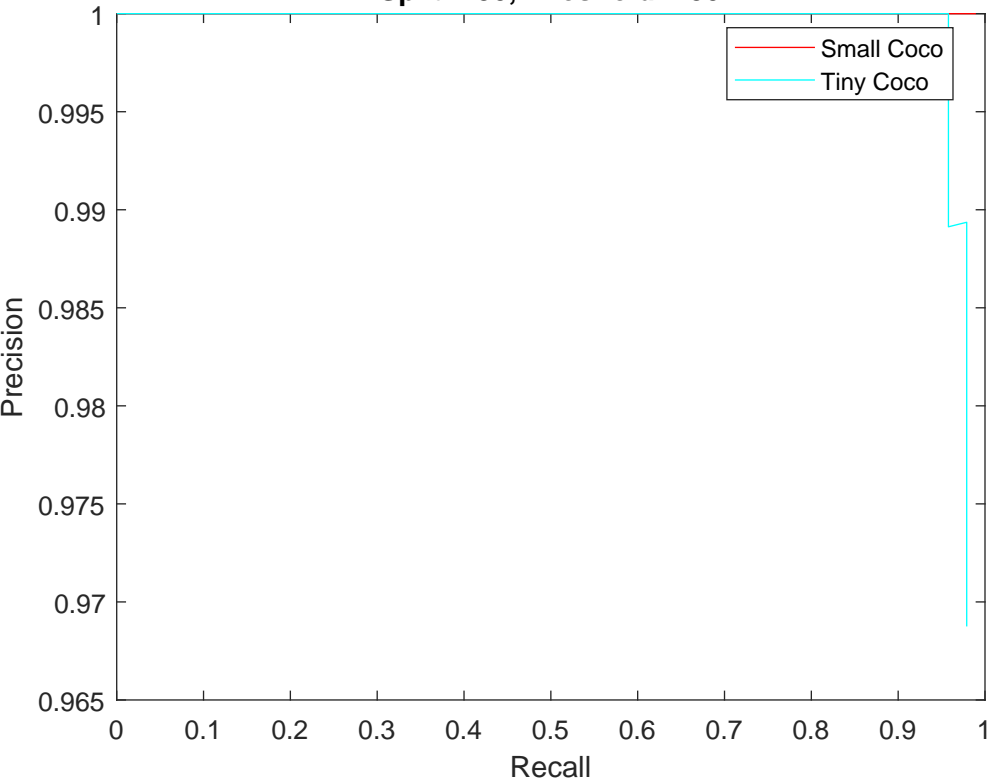

Supplement: S47 Fig — (PDF) [file pone.0312763.s050.pdf]

Split = 80, Theshold = 70

Precision

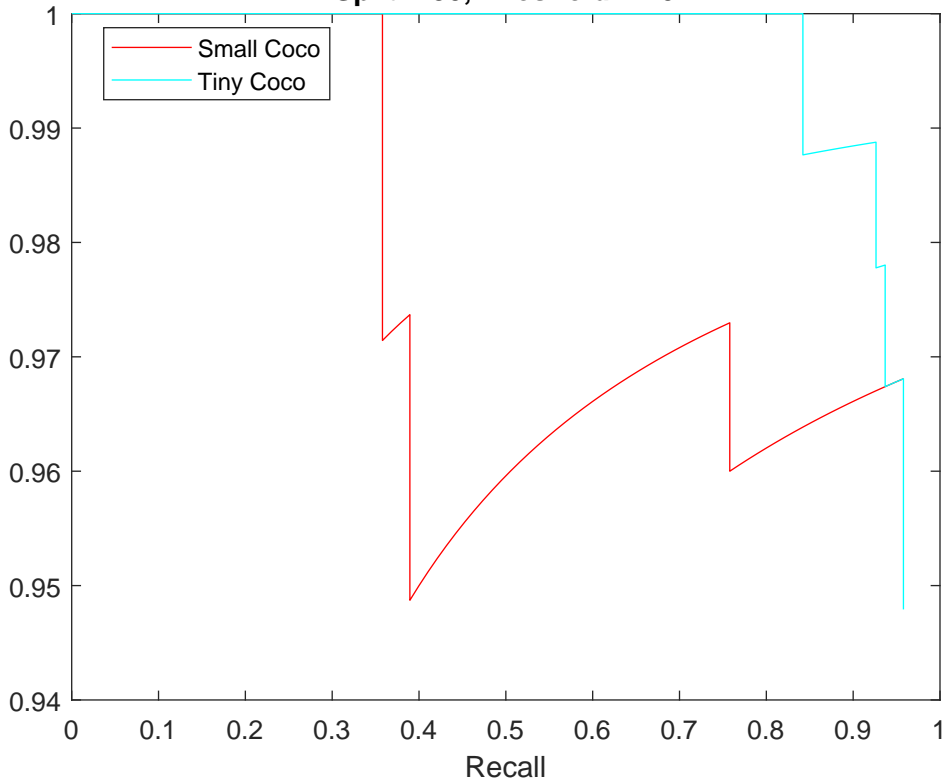

Supplement: S48 Fig — (PDF) [file pone.0312763.s051.pdf]
